# New Hippolide Derivatives with Protein Tyrosine Phosphatase 1B Inhibitory Activity from the Marine Sponge *Hippospongia lachne*

**DOI:** 10.3390/md12074096

**Published:** 2014-07-08

**Authors:** Shu-Juan Piao, Wei-Hua Jiao, Fan Yang, Yang-Hua Yi, Ying-Tong Di, Bing-Nan Han, Hou-Wen Lin

**Affiliations:** 1 Laboratory of Marine Drugs, Department of Pharmacy, Changzheng Hospital, Second Military Medical University, Shanghai 200003, China; E-Mails: piaoshujuan@163.com (S.-J.P.); yiyanghua@126.com (Y.-H.Y.); 2 Key Laboratory for Marine Drugs, Department of Pharmacy, Renji Hospital, Shanghai Jiao Tong University School of Medicine, Shanghai 200127, China; E-Mails: jiaoweihua1982@sina.com (W.-H.J.); bill1985@126.com (F.Y.); 3 Laboratory of Phytochemistry and Plant Resources in West China, Kunming Institute of Botany, Chinese Academy of Sciences, Kunming 650201, Yunnan, China; E-Mail: diyt@mail.kib.ac.cn

**Keywords:** marine sponge, *Hippospongia lachne*, sesterterpenoids, absolute configuration, PTP1B

## Abstract

Five new sesterterpenoids, compounds **1**–**5**, have been isolated from the sponge *Hippospongia lachne* off Yongxing Island in the South China Sea. The structures of compounds **1**–**5** were elucidated through extensive spectroscopic analysis, including HRMS, 1D, and 2D NMR experiments. The stereochemistry, including absolute configurations of these compounds, was determined by spectroscopic, chemical, and computational methods. Compounds **1** and **5** showed moderate protein tyrosine phosphatase 1B (PTP1B) inhibitory activities with IC_50_ values of 5.2 μM and 8.7 μM, respectively, more potent than previously reported hippolides.

## 1. Introduction

Protein tyrosine phosphatase 1B (PTP1B), as a therapeutic target for the treatment of Type-II diabetes and obesity, has been the subject of intense study over the past decade [1,2,3,4,5]. Approximately 300 new or known natural products with PTP1B inhibitory activity, have been isolated and identified from various natural resources, many of which are of marine origin [2]. Since the discovery of sulfircin, a sesterterpene sulfate as the first reported marine natural product with PTP1B inhibitory activity, isolated from a deep-water sponge *Ircinia* (unknown species), marine sponges have proven to be a valuable source of structurally diverse molecules with PTP1B inhibitory activity [4], such as polybromodiphenyl ether from *Lamellodysidea herbacea* [6], and sesquiterpenoids and sesquiterpene quinones from sponge *Dysidea villosa* [7].

**Figure 1:**
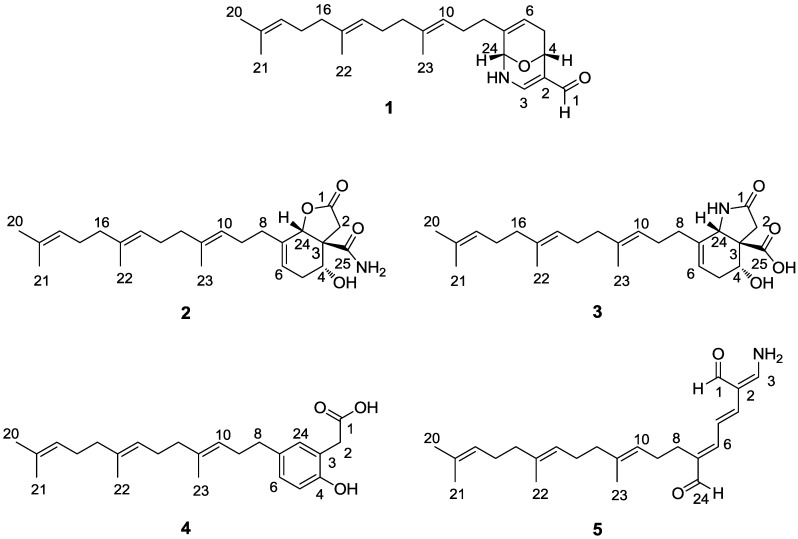
Structures of compounds **1**–**5**.

Marine sponges of the genus *Hippospongia* (family Spongiidae, order Dictyoceratida) have attracted a great deal of attention as they contain bioactive sesquiterpenes [8,9,10,11], sesterterpenes and sulfates [12,13,14], furanoterpenes [15], triterpenoic acids [16], and polyketides [17]. In our screening program to search for bioactive marine natural products from the South China Sea sponge *Hippospongia lachne*, we have previously obtained eight new cytotoxic acyclic manoalide-related sesterterpenes, hippolides A–H, with potent cytotoxicity against A549, HeLa, and HCT-116 cell lines [15]. Interestingly, further investigation of crude fractions with PTP1B inhibitory activity from the same sample led to the isolation of a series of new hippolides-related sesterterpenes, compounds **1**–**5** (Figure 1), which exhibited different levels of PTP1B inhibitory activity with an IC_50_ values of 5.2, >50, 33, 14, and 8.7 μM, respectively. Herein, we report the isolation, structure elucidation, and bioactivity of these compounds.

## 2. Results and Discussion

The sponge *H. lachne* was collected off Yongxing Island and seven connected islets in the South China Sea. The organic extract (110 g) was subjected to chromatography on a silica gel column, followed by consecutive Sephadex LH-20 and RP-HPLC chromatography to yield five new sesterterpenoids, compounds **1**–**5** (Figure 1).

**Table 1:** ^13^C NMR Data of Compounds **1**–**5** (CDCl_3_).

Carbon	1 *^a^*	2 *^a^*	3 *^b^*	4 *^b^*	5 *^b^*
1	186.5 CH	174.5 qC	178.5 qC	181.9 qC	190.2 CH
2	117.3 qC	33.9 CH_2_	30.4 CH_2_	42.0 CH_2_	108.0 CH
3	146.5 CH	53.1 qC	58.6 qC	123.3 qC	151.8 qC
4	65.6 CH	67.8 CH	67.4 CH	153.5 qC	139.7 CH
5	31.0 CH_2_	29.8 CH_2_	31.5 CH_2_	117.0 CH	114.2 CH
6	120.1 CH	122.2 CH	119.7 CH	128.2 CH	150.9 CH
7	134.8 qC	135.1 qC	138.8 qC	134.5 qC	138.2 qC
8	33.0 CH_2_	33.2 CH_2_	31.9 CH_2_	35.4 CH_2_	24.5 CH_2_
9	26.0 CH_2_	25.7 CH_2_	26.8 CH_2_	30.5 CH_2_	27.4 CH_2_
10	124.0 CH	123.1 CH	123.6 CH	123.9 CH	123.5 CH
11	135.7 qC	136.2 qC	136.0 qC	135.8 qC	136.1 qC
12	39.7 CH_2_	39.7 CH_2_	39.7 CH_2_	39.9 CH_2_	39.7 CH_2_
13	26.6 CH_2_	26.6 CH_2_	26.7 CH_2_	26.8 CH_2_	26.7 CH_2_
14	123.2 CH	124.0 CH	124.1 CH	124.4 CH	124.2 CH
15	126.2 qC	135.0 qC	135.1 qC	135.1 qC	135.0 qC
16	39.7 CH_2_	39.7 CH_2_	39.7 CH_2_	39.9 CH_2_	39.7 CH_2_
17	26.8 CH_2_	26.7 CH_2_	26.7 CH_2_	26.9 CH_2_	26.8 CH_2_
18	124.4 CH	124.3 CH	124.4 CH	124.6 CH	124.4 CH
19	131.4 qC	131.4 qC	131.3 qC	131.4 qC	131.3 qC
20	25.7 CH_3_	25.7 CH_3_	25.7 CH_3_	25.7 CH_3_	25.7 CH_3_
21	17.7 CH_3_	17.7 CH_3_	17.7 CH_3_	17.7 CH_3_	17.7 CH_3_
22	16.0 CH_3_	16.0 CH_3_	16.0 CH_3_	16.0 CH_3_	16.0 CH_3_
23	16.2 CH_3_	16.1 CH_3_	16.1 CH_3_	16.0 CH_3_	16.1 CH_3_
24	76.9 CH	79.7 CH	70.8 CH	130.9 CH	194.2 CH
25		175.2 qC	182.9 qC		

*^a^* Recorded at 100 MHz; *^b^* Recorded at 125 MHz.

**Figure 2:**
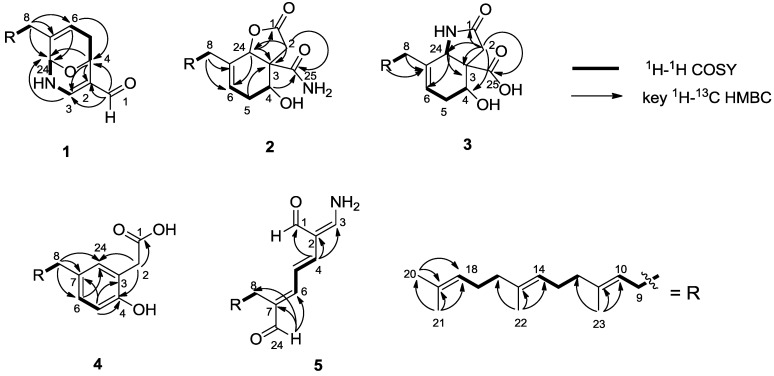
Selected ^1^H–^1^H COSY and ^1^H–^13^C HMBC correlations of **1**–**5**.

Compound **1** was obtained as colorless oil. The HR-ESI-MS (high resolution electrospray ionization mass spectrometry) data for **1** gave an adduct ion [M + Na]^+^ at 392.2567, consistent with a molecular formula of C_24_H_35_NO_2_, implying eight degrees of unsaturation. The 1D and 2D NMR spectra (Supplementary Information) clearly indicated the presence of one aldehyde group (δ_C_ 186.5; δ_H_ 8.97 1H, s); ten olefinic carbons (δ_C_ 117.3, 146.5, 120.1, 134.8, 124.0, 135.7, 123.2, 126.2, 124.4, and 131.4), correlated with five olefinic protons at δ_H_ 7.13 (1H, d, *J* = 5.6 Hz), 5.66 (1H, d, *J* = 5.0 Hz), 5.11 (1H, m), 5.10 (1H, m), and 5.08 (1H, m); two oxygen-bearing sp^3^ methines (δ_C_ 65.6, δ_H_ 5.06; δ_C_ 76.9, δ_H_ 5.03); seven methylenes, and four methyl carbons (Table 1 and Table 2). The ^1^H NMR spectrum also displayed a NH proton resonance at δ_H_ 5.78 (1H, br s) with no correlation in HSQC spectrum. The COSY correlations for H-8/H-9/H-10, H-12/H-13/H-14, and H-16/H-17/H-18, together with the HMBC correlations of four methyl groups, H_3_-20/C-18, C-19, and C-21, H_3_-21/C-18, C-19, H_3_-22/C-14, C-15, and C-16, and H_3_-23/C-10, C-11, and C-12, delineated the presence of a farnesyl moiety (Figure 2). In addition, the COSY correlations of H-4/H-5/H-6, and H-3/NH/H-24 as well as the HMBC correlations from H-6 to C-4 and C-24, H-4 to C-2, C-3 and C-24, and H-3 to C-24, revealed a 9-oxa-2-azabicyclo-[3,3,1]-nona-3,7-diene moiety. Furthermore, the HMBC correlations of H-1/C-2, C-3, and C-4 indicated that the aldehyde group was attached to the bicyclo-moiety at C-2. The aforementioned two moieties were connected at C-7, with the evidence of ^2^*J*_CH_ and ^3^*J*_CH_ correlations from H-8 to C-7 and C-6, C-24, respectively. Therefore, the eight degrees of unsaturation were accounted for by five double bonds, one aldehyde moiety, and two rings.

**Table 2:** ^1^H NMR Data of Compounds **1**–**5** (CDCl_3_, *J* in Hz).

Position	1 *^a^*	2 *^a^*	3 *^b^*	4 *^b^*	5 *^b^*
1	8.97, s				9.75, d (3.5)
2		2.93, d (18.0); 2.77, d (18.0)	2.69, s	3.53, s	
3	7.13, d (5.6)				7.70, dt (3.5, 11.5)
4	5.03, d (5.0)	4.21, dd (7.0, 1.8)	4.22, dd (9.5, 3.6)		6.64, d (14.7)
5	2.64, dd (5.0, 20.0) 2.12, m	2.46, m; 2.29, m	2.39, m; 2.06, m	6.78, d (8.0)	6.66, m
6	5.66, d (5.0)	5.74, t (3.9)	5.35, t (3.9)	6.93, d (8.0)	6.86, d (11.0)
8	1.99, m	2.19, m	2.06, m	2.51, t (8.0)	2.39, t (7.5)
9	2.13, m	2.17, m	2.06, m	2.22, m	2.12, m
10	5.11, m	5.09, m	5.10, m	5.17, t (6.8)	5.16, t (7.5)
12	1.97, m	1.97, m	2.69, m	1.98, m	1.96, m
13	2.05, m	2.07, m	2.06, m	2.06, m	2.05, m
14	5.10, m	5.09, m	5.10, m	5.11, t (6.8)	5.11, t (6.8)
16	1.97, m	1.97, m	1.98, m	1.98, m	1.95, m
17	2.05, m	2.07, m	2.06, m	2.06, m	2.05, m
18	5.08, m	5.09, m	5.10, m	5.10, t (6.3)	5.09, t (6.5)
20	1.68, s	1.68, s	1.68, s	1.68, s	1.68, s
21	1.60, s	1.60, s	1.59, s	1.60, s	1.60, s
22	1.60, s	1.60, s	1.59, s	1.60, s	1.58, s
23	1.60, s	1.60, s	1.59, s	1.57, s	1.60, s
24	5.06, d (4.1)	5.14, s	4.58, s	6.89, br s	9.37, s
25					
OH *^c^*		4.99, br s	11.04, s; 4.02, br s		
NH	5.78, br s (in CDCl_3_)	7.56 *^c^*, s; 7.26, s	5.42 *^c^*, d (7.5)		8.20 *^c^*, m

*^a^* Recorded at 400 MHz; *^b^* Recorded at 500 MHz; *^c^* Recorded at 500 MHz in DMSO.

**Figure 3:**
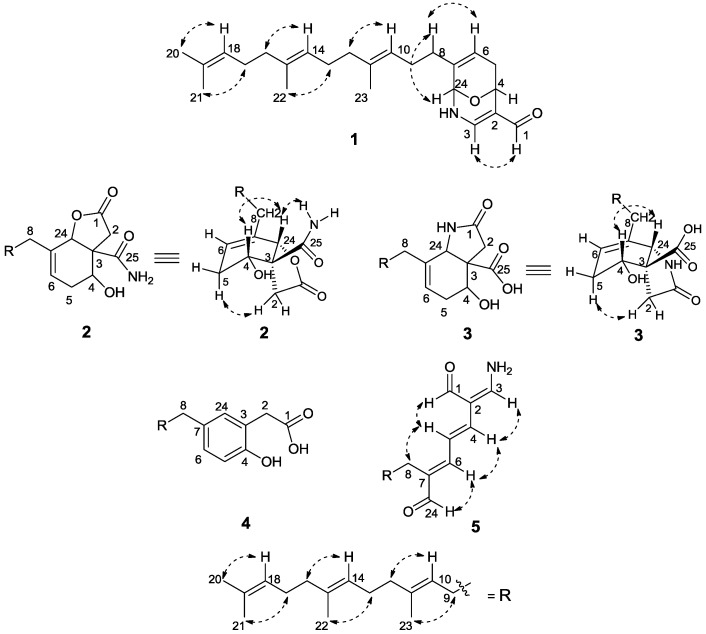
Selected NOE correlations of **1**–**5**.

**Figure 4:**
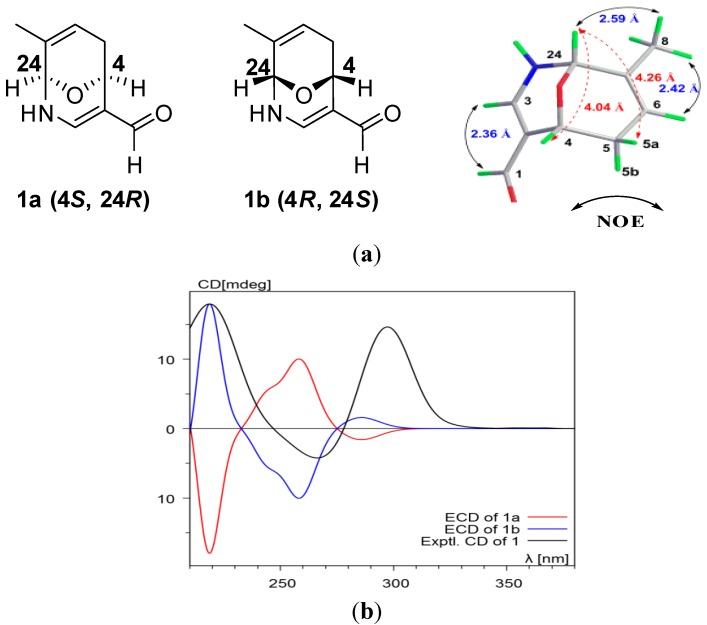
(**a**) Simplified structures (**1a**,**b**) of two possible enantiomers of **1** and computational modeling of **1b** (4*R*, 24*S*); (**b**) Calculated ECD spectra of (4*S*, 24*R*)- and (4*R*, 24*S*)- two enantiomers and experimental ECD spectrum of **1**.

The relative configuration of **1** was determined by the analysis of NOESY correlations (Figure 3) and computational method (Supplementary Information). The NOESY correlations of H-10/H-12, H-9/H_3_-23, H-14/H-16, and H-13/H_3_-22 suggested a 10*E*, 14*E* double bond geometry in the farnesyl moiety. However, the remaining stereochemistry of **1** could not be ambiguously assigned by the observed NOESY correlations as shown in Figure 3. Consequently, the computational approach was used to determine the relative structure of 9-oxa-2-azabicyclo-[3,3,1]-nona-3,7-diene moiety. To reduce computational cost, the aliphatic chain of **1** was shortened, as the long aliphatic chain may generate various conformations but has little effect on systematic analysis of all the possible stereoisomers [18,19,20]. On the basis of the relative geometry of H-4 and H-24, as well as the NOSEY correlations indicated in 1a (Figure 3), only four configurations were modeled for theoretical calculations to identify the most energetically reasonable configuration of **1** (Supplementary Information). Conformational analysis using MMFF94, followed by energy optimization at HF/6-31G(d) levels in GAUSSIAN 03 (for details see Supplementary Information), suggested that the *cis* orientation of H-4/H-24, with *syn* relationships to the oxo bridge as shown in **1A** (Supplementary Information, Energy minimization and ECD calculations) retained the most favorable configuration with respect to the energy minimization. The *trans* relationships for H-4 and H-24 were eliminated due to the severe distortions of the sp^3^ atoms with highly unfavorable energy involved in the associated rings (Supplementary Information). To establish the absolute configuration of compound **1**, its electronic circular dichroism (ECD) spectrum was experimentally recorded, which showed positive Cotton effect at 219 and 297 nm. The theoretical ECD of **1a** and its enantiomer **1b** were then calculated with a time-dependent density function theory (TD-DFT) method at the b3lyp/aug-cc-pvdz level, and the polarizable continuum model (PCM) was adopted to consider solvent effects using the dielectric constant of methanol. The calculated ECD spectra were produced by SpecDis software in Figure 4. The overall pattern of calculated spectrum of **1b** was in good agreement with the experimental one. Thus, the absolute configurations at C-4, and C-24 of **1** were determined as *R* and *S*, respectively.

**Figure 5:**
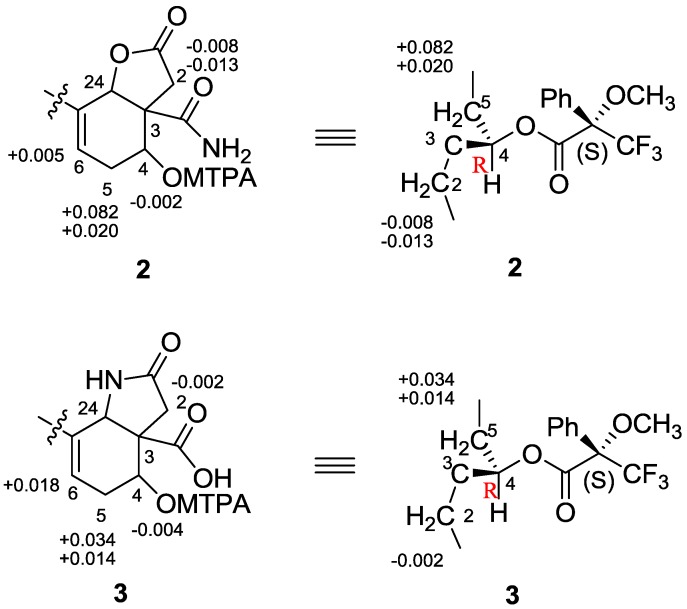
∆δ_*S–R*_ values (ppm) for the MTPA derivatives of **2** and **3** in CDCl_3_.

Compound **2**, was assigned a molecular formula of C_25_H_37_NO_4_ with 8 degrees of unsaturation, based on HR-ESI-MS data for [M + Na]^+^ *m*/*z* 438.2619. The ^13^C NMR and DEPT spectra indicated 25 resonances for four methyls, eight methylenes, six methines (two oxygen-bearing *sp*^3^ methines), and seven quaternary carbons (two carbonyl carbons) (Table 1). The ^1^H NMR spectrum displayed resonances for four olefinic protons at δ_H_ 5.74 (1H, t, *J* = 3.9 Hz ), and 5.09 (3H, m, overlapped), two oxygenated methine protons at δ_H_ 5.14 (1H, s), and 4.21 (1H, dd, *J* = 7.0, 1.8 Hz), four methyl groups at δ_H_ 1.68 (3H, s), and 1.60 (9H, s, overlapped), in addition to two NH_2_ protons at δ_H_ 7.56 (1H, s, in DMSO-*d*_6_) and 7.26 (1H, s, in DMSO-*d*_6_), and one OH proton at δ_H_ 4.99 (1H, s, in DMSO-*d*_6_). Inspection of the ^1^H and ^13^C NMR, HMBC and COSY spectra of **2** suggested that it was structurally related to compound **1**, possessing a farnesyl moiety. Six of the eight degrees of unsaturation of **2** were accounted for by four double bonds and two carbonyl carbons, implying that the structure retained two rings as well. Interpretation of the COSY correlations of H-4/H-5a, 5b/H-6, and H-4 with an exchangeable proton at δ_H_ 4.99 (4-OH in DMSO-*d*_6_, not shown), together with the HMBC correlations of H-24/C-6 and C-3, H-5a/C-3, and C-4, indicated the presence of a 4-hydroxycyclohexenyl moiety. The connection of the farnesyl group to the 4-hydroxycyclohexenyl moiety at C-7 was supported by the HMBC correlations of H-8/C-6 and C-7. The remaining ring structure was assigned to a butanolide moiety attached to the 4-hydroxycyclohexenyl moiety via an ester linkage at C-24 and a quaternary carbon at C-3, which was delineated by the HMBC correlations of H-24/C-1 and C-3, H-2a, H-2b/C-1 and C-3 and C-24. The last unassigned substructure CONH_2_ was determined as an acetamide motif based on the chemical shift of the carbonyl carbon (δ_C_ 175.2), and two NH_2_ protons at δ_H_ 7.56, 7.26 in DMSO-*d*_6_, as well as the IR absorption bands (ν_max_ 3427, 1671 cm^−1^). Furthermore, the ^3^*J*_CH_ correlations from H-2, H-4, and H-24 to C-25 indicated that the acetamide group was attached to the bicyclo-moiety at C-3. The NOESY correlations for H-10/H-12, H-9/H_3_-23, H-14/H-16, and H-13/H_3_-22 (Figure 2) suggested the 10*E*, 14*E* double bond geometry in the farnesyl moiety. The *syn*-relationship of H-4, H-24 and NH_2_ was determined on the basis of NOESY correlations of H-4/H-24/NH_2_ (Figure 3). The absolute configuration of C-4 was assigned by application of the modified Mosher method [14]. The (*S*)- and (*R*)-MTPA esters of **2** were prepared by treatment with (*R*)- and (*S*)-MTPA chloride, respectively. The ∆δ_*S–R*_ values of the Mosher ester observed for the protons near the secondary C-4 hydroxy group indicated the *R* configuration for the carbinol stereogenic center in **2** (Figure 5). On the basis of its relative configuration (Figure 3), the absolute configuration of **2** was, thus, determined as 3*S*, 4*R*, 24*S*.

**Figure 6:**
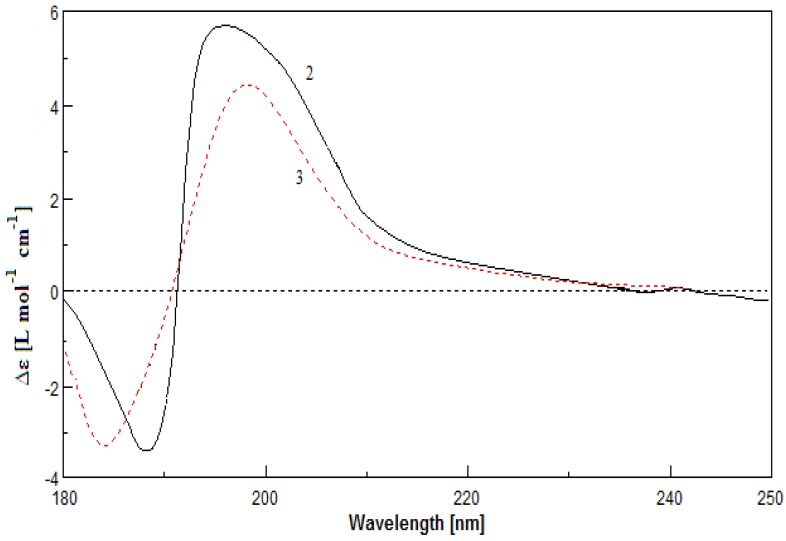
CD curves of compounds **2** and **3**.

Compound **3** exhibited the same molecular formula of C_25_H_37_NO_4_ ([M + Na]^+^ *m*/*z* 438.2622) as that of **2**. A detailed analysis of the 1D and 2D NMR spectroscopic data confirmed that **3** possessed the farnesyl and 4-hydroxycyclohexenyl moieties as well. However, careful comparison of ^1^H NMR spectra of compounds **2** and **3** revealed that they are isomers, in which the δ_H_ 5.14 of H-24 in **2** (1H, s, in CDCl_3_) changed to δ_H_ 4.58 (1H, s, in CDCl_3_) in **3**, and displayed clear correlation with NH proton at δ_H_ 5.42 (1H, d, *J* = 7.5 Hz, in DMSO-*d*_6_) in the COSY spectrum of **3**. In addition, the resonance of ^1^H NMR for **3** indicated the presence of one carboxyl proton at δ_H_ 11.04 (1H, s, in DMSO-*d*_6_). The HMBC of H-2/C-1, C-3, C-4, C-24, and C-25, and COSY correlations of NH/H-24, H-6/H-5a/H-4 (Figure 2) suggested that **3** possessed a pyrrolidin-2-one moiety and a carboxy group (C-25) at C-3 instead of butenolide moiety and carbamoyl group in **2**. The NOESY correlations for H-10/H-12, H-9/H_3_-23, H-14/H-16, and H-13/H_3_-22 (Figure 3) confirmed that the farnesyl moiety in **3** remained the same configuration as the 10*E*, 14*E* in **2**. The *syn*-relationship of H-4 and H-24 was indicated by the NOESY correlations of H-4/H-24 (Figure 3). The absolute configuration of C-4 was assigned by application of the modified Mosher method as well. The ∆δ_*S–R*_ values observed for the protons near the secondary C-4 hydroxy group for the esters indicated the *R* configuration for the carbinol stereogenic center in **3** (Figure 5). On the basis of its relative configuration, as well as the analysis of CD spectra of compounds **2** and **3** showing similar Cotton effects near 195 nm (Figure 6), the absolute configuration of compound **3** was suggested as 3*S*, 4*R*, 24*S*.

The adduct ion of compound **4** at *m*/*z* 393.2404 [M + Na]^+^ in HR-ESI-MS determined the molecular formula of **4** as C_24_H_34_O_3_, which is supported by ^13^C NMR data (Table 1). The ^1^H, ^13^C NMR spectra of **4** were indicative of the hippolide-like metabolite, containing a farnesyl group with the proton signals at δ_H_ 5.17 (1H, t, *J* = 6.8 Hz), 5.11 (1H, t, *J* = 6.8 Hz), and 5.10 (1H, t, *J* = 6.3 Hz). In addition, the analysis of HSQC and DEPT data delineated the presence of a substituted benzene ring with three signals at δ_C_ 117.0/δ_H_ 6.78, at δ_C_ 128.2/δ_H_ 6.93, and at δ_C_ 130.9/δ_H_ 6.89. The HMBC correlations of H-5/C-3, C-4, and C-7, H-6/C-4, C-5, and C-24, H-8/C-6, C-7, and C-24, as well as the COSY correlations of H-5/H-6 revealed that this benzene ring is substituted at three positions, C-3, C-4 and C-7, with the connection of the farnesyl group at C-7. The ^13^C NMR and DEPT data indicated C-4 is an oxygenated quaternary carbon, which characterized the phenolic identity of compound **4**. Furthermore, an acetic acid moiety was elucidated to be attached to the phenolic ring at C-3 using the assemblage of 1D and 2D experiments, in which methylene protons of H-2 (δ_H_ 3.53, 2H, s) have displayed HMBC correlations with C-1 (δ_C_ 181.9, a carboxylic carbon), C-3, C-4, and C-24. The NOESY correlations for H-10/H-12, H-9/H_3_-23, H-14/H-16, and H-13/H_3_-22 (Figure 3) suggested the 10*E*, 14*E*-configuration of the farnesyl group.

The molecular formula of compound **5** was established as C_24_H_35_NO_2_ on the basis of HR-ESI-MS (*m*/*z* 392.2564, [M + Na]^+^) and NMR spectral data (Table 1 and Table 2), indicative of eight degrees of unsaturation. The ^1^H NMR spectrum displayed resonances for two aldehyde protons at 9.75 (1H, d, *J* = 3.5 Hz) and 9.37 (1H, s), seven olefinic protons at δ_H_ 7.70 (1H, dt, *J* = 3.5, 11.5 Hz), 6.86 (1H, d, *J* = 11.0 Hz), 6.66 (1H, m), 6.64 (1H, d, *J* = 14.7 Hz), 5.16 (1H, t, *J* = 7.5 Hz), 5.11 (1H, t, *J* = 6.8 Hz), and 5.09 (1H, t, *J* = 6.5 Hz), and four methyl groups at δ_H_ 1.68 (3H, s), 1.60 (6H, s, overlapped), and 1.58 (3H, s). The HMBC and COSY correlations (Figure 1) suggested that **5** possessed a farnesyl group as well. A -NH_2_ motif was clarified by the presence of two exchangeable NH_2_ proton resonances at δ_H_ 8.20 (2H, m, in DMSO-*d*_6_), as well as the presence of IR band at 3379 cm^−1^. In addition, the olefinic proton at δ_H_ 7.70 (H-2) displayed the ^4^*J*_H-1/H-2_ and ^3^*J*_H-3/NH_ as doublets of triplets (1H, dt, *J* = 3.5, 11.5 Hz), revealing the NH_2_ motif to be attached at C-3 position. Furthermore, the HMBC correlations of H-1/C-2, H-4/C-1, C-2, C-3, and H-24/C-6, C-7, and C-8, together with the COSY correlations of H-1/H-3 (W effect), and H-4/H-5/H-6 confirmed the presence of an 2-(aminomethylene) hepta-3,5-dienedial moiety connected with the farnesyl group at C-7. Therefore, the eight degrees of unsaturation were accounted for by six double bonds, two aldehyde moieties. The NOESY correlations for H-3/H-4, H-5/H-8, H-6/H-24, H-10/H-12, H-9/H_3_-23, H-14/H-16, and H-13/H_3_-22 (Figure 3) suggested the geometry of double bonds *Δ*^2,3^, *Δ*^4,5^, *Δ*^6,7^, *Δ*^10,11^, * Δ*^14,15^, as 3*Z*, 4*E*, 6*E*, 10*E*, 14*E*, respectively.

To further confirm the original PTP1B activity from the crude fraction of the title sponge, compounds **1**–**5** were evaluated *in vitro* for PTP1B inhibitory activity. Compound **1** and **5** exhibited moderate PTP1B inhibitory activities with IC_50_ values of 5.2 and 8.7 μM, but compounds **3**, and **4** exhibited weak PTP1B inhibitory activities with an IC_50_ values of 33, 14 μM, respectively. The oleanolic acid was used as a positive control for the PTP1B experiment with an IC_50_ value of 2.0 µM. We have also evaluated the cytotoxicity of compounds **1**–**5** against A549, HeLa, and HCT-116 cancer cell lines, however, only compound **1** exhibited weak activity against HCT-116 cell line with an IC_50_ value of 11.6 µM and no activity was observed for other compounds.

## 3. Experimental Section

### 3.1. General Experimental Procedures

IR spectra were recorded on a Bruker vector 22 spectrometer (Bruker Optics, Inc., Billerica, MA, USA) with KBr pellets. Optical rotation data were recorded on a Perkin-Elmer model 341 polarimeter (Perkin-Elmer, Inc., Waltham, MA, USA) with 1 dm cell. The CD spectra were obtained with a JASCO J-715 spectropolarimeter (Jasco Inc., Tokyo, Japan). The NMR experiments were measured on Bruker AMX-400 MHz and AMX-500 MHz instruments (Bruker Biospin Corp., Billerica, MA, USA) in CDCl_3_ with TMS as an internal standard. ESIMS and HRESIMS spectra were recorded on a Waters Q-Tof micro YA019 mass spectrometer (Waters Corp., Milford, MA, USA). Reversed-Phase HPLC was performed on a YMC-Pack Pro C_18_ RS column (250 × 10 mm, 5 µm) using a Waters 600 HPLC instrument (Waters Corp., Milford, MA, USA) with Waters 996 UV detector. Column chromatography (CC) was performed on Sephadex LH-20 (Pharmacia Fine Chemicals, Piscataway, NJ, USA) and YMC ODS-A (50 μm) (250 × 10 mm, YMC Co., Ltd., Kyoto, Japan). Vacuum liquid chromatography (VLC) was performed on silica gel (200–300 mesh, Qingdao Ocean Chemical Co., Jinan, China); the fractions were monitored by TLC (HSGF 254, Yantai Huiyou Co., Yantai, China) and spots were visualized by heating silica gel plates sprayed with 10% H_2_SO_4_ in H_2_O.

### 3.2. Animal Material

A specimen of *H. lachne* was collected off Yongxing Island and seven connected islets in the South China Sea in June 2007, and was identified by Jin-He Li (Institute of Oceanology, Chinese Academy of Sciences, Qingdao, China). A voucher sample (No. B-2) was deposited in Laboratory of Marine Drugs, Department of Pharmacy, Changzheng Hospital, Second Military Medical University, Shanghai, China.

### 3.3. Extraction and Isolation

The sponge (3.6 kg, wet weight) was extracted with 95% aq. EtOH and combined extracts were concentrated under reduced pressure to yield the crude extract (671 g). This extract was suspended in H_2_O and extracted with EtOAc and *n*-BuOH to afford the EtOAc- and *n*-BuOH-soluble extracts (232 g and 78 g, respectively). The EtOAc-soluble extract was partitioned between MeOH–H_2_O (9:1) and petroleum ether to yield a brownish red oil (84 g). The MeOH–H_2_O (9:1) phase was diluted 3:2 with H_2_O and extracted with CH_2_Cl_2_ to afford the CH_2_Cl_2_-soluble extract (110 g). This CH_2_Cl_2_-soluble extract was subjected to VLC on silica gel using CH_2_Cl_2_/MeOH (100:1, 50:1, 30:1, 20:1, 10:1, 5:1, and 1:1) as eluent to give nine sub-fractions (A-G). Sub-fraction C with PTP1B inhibitory activity was subjected to CC on Sephadex LH-20 (CH_2_Cl_2_:MeOH = 1:1) and silica gel using Petroleum ether/Acetone (30:1, 20:1, 10:1, 5:1, 3:1, and 1:1) to give eight sub-fractions (a–i). Sub-fractions (b–d) were further purified by HPLC (YMC-Pack Pro C_18_ RS, YMC Co., Ltd., Kyoto, Japan, 5 µm, 10 × 250 mm, 2.0 mL/min, UV detection at 210 and 254 nm) eluting with MeOH/H_2_O (80:20) to yield pure compounds **1** (6.4 mg), **4** (8.9 mg), and **5** (12.0 mg) at 56, 85, and 93 min, respectively. Similarly, compounds **2** (3.9 mg), and **3** (5.1 mg) were purified from sub-fractions h and e using HPLC (YMC-Pack Pro C_18_ RS, 5 µm, 10 × 250 mm, 2.0 mL/min, UV detection at 210 and 254 nm) in MeOH/H_2_O (83:17) at 28 and 23 min, respectively.

**Compound 1**: colorless oil; 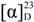 +67° (*c* 0.120, MeOH); CD (MeOH) λ_max_ (∆ε) 219 (3.40), 266 (−0.80), 297 (2.77) nm; UV (MeOH) λ_max_ (log ε) 210 (1.68), 233 (1.64), 282 (1.56) nm; IR (KBr) ν_max_ 3369, 2926, 1596, 1448, 1212, 1067, 840, 673 cm^−1^; ^1^H and ^13^C NMR data, see Table 1 and Table 2; HRESIMS *m*/*z* 392.2567 [M + Na]^+^ (calcd for C_24_H_35_NO_2_Na^+^, 392.2560).

**Compound 2**: colorless oil; 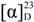 −15° (*c* 0.10, MeOH); CD (MeOH) λ_max_ (∆ε) 189 (−3.48), 196 (5.71) nm; UV (MeOH) λ_max_ (log ε) 190 (3.30) nm; IR (KBr) ν_max_ 3427, 2924, 1773, 1671, 1377, 1189, 1097, 981 cm^−1^; ^1^H and ^13^C NMR data, see Table 1 and Table 2; ^1^H NMR data (in DMSO): δ_H_ 7.56 (1H, s, NH), 7.26 (1H, s, NH), 5.54 (1H, br s, H-24), 4.99 (1H, br s, OH), 5.14 (1H, m, H-6), 4.10 (1H, m, H-4); HRESIMS *m*/*z* 438.2619 [M + Na]^+^ (calcd for C_25_H_37_NO_4_Na^+^, 438.2615).

**Compound 3**: colorless oil; 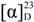 −10° (*c* 0.12, MeOH); CD (MeOH) λ_max_ (∆ε) 184 (−3.30), 198 (4.39) nm; UV (MeOH) λ_max_ (log ε) 195 (3.84), 223 (1.48) nm; IR (KBr) ν_max_ 3434, 2923, 1712, 1374, 1199, 1038, 742 cm^−1^; ^1^H and ^13^C NMR data, see Table 1 and Table 2; ^1^H NMR data (in DMSO): δ_H_ 11.04 (1H, s, OH), 5.42 (1H, d, *J* = 7.5 Hz, NH), 5.22 (1H, m, H-6), 4.30 (1H, br s, H-24), 4.02 (1H, br s, OH), 3.86 (1H, m, H-4); HRESIMS *m*/*z* 438.2622 [M + Na]^+^ (calcd for C_25_H_37_NO_4_Na^+^, 438.2615).

**Compound 4**: yellow oil; UV (MeOH) λ_max_ (log ε) 228 (3.20), 282 (3.16) nm; IR (KBr) ν_max_ 3415, 2923, 1566, 1439, 1377, 1248, 1015, 823, 651 cm^−1^; ^1^H and ^13^C NMR data, see Table 1 and Table 2; HRESIMS *m*/*z* 393.2404 [M + Na]^+^ (calcd for C_24_H_34_O_3_Na^+^, 393.2400).

**Compound 5**: yellow oil; UV (MeOH) λ_max_ (log ε) 365 (2.56) nm; IR (KBr) ν_max_ 3379, 2925, 1582, 1441, 1380, 1234, 1188, 1126 cm^−1^; ^1^H and ^13^C NMR data, see Table 1 and Table 2; ^1^H NMR data (in DMSO): δ_H_ 9.33 (1H, s, H-1), 9.04 (1H, s, H-24), 8.20 (2H, m, NH2), 7.70 (1H, m, H-5), 7.29 (1H, m, H-3), 6.97 (1H, d, *J* = 14.5 Hz, H-4), 6.92 (1H, d, *J* = 12.0 Hz, H-6); HRESIMS *m*/*z* 392.2564 [M + Na]^+^ (calcd for C_24_H_35_NO_2_Na^+^, 392.2560).

### 3.4. PTP1B Inhibitory Assay

PTP1B inhibitory activity was determined using a PTP1B inhibitory assay as described in a previous report [21]. The enzymatic activities of the PTP1B catalytic domain were determined at 30 °C by monitoring the hydrolysis of *p*NPP. Dephosphorylation of *p*NPP generates product *p*NP, which was monitored at an absorbance of 405 nm. In a typical 100 μL assay mixture containing 50 mmol/L 3-[*N*-morpholino] propanesulfonic acid (MOPs), pH 6.5, 2 mmol/L *p*NPP, and 30 nmol/L recombinant PTP1B, activities were continuously monitored and the initial rate of the hydrolysis was determined using the early linear region of the enzymatic reaction kinetic curve.

### 3.5. Computational Details of Calculated ECD

All quantum-chemical calculations were performed by the Gaussian 03 program. The TD calculations were calculated by b3lyp/aug-cc-pvdz method under Self-Consistent Reaction Field model of solvent (MeOH). Details of the DFT calculation see Supplementary Information.

### 3.6. Preparation of MTPA Esters

A previously described modified Mosher’s method was used [15]. The (*S*)- and (*R*)-MTPA esters of **2** (3*S*, 3*R*) and **3** (4*S*, 4*R*) were obtained by treatment of **2** (0.5 mg and 0.6 mg, respectively) and **3** (0.7 mg and 0.7 mg, respectively) with (*R*)- and (*S*)-MTPA chlorides (10 μL) in dry pyridine (0.5 mL), and stirred at room temperature overnight. The MTPA esters were purified by mini-column chromatography on silica gel (200 mesh, CH_2_Cl_2_/MeOH, 13:1 of **2** and **3**).

## 4. Conclusions

Protein tyrosine phosphatase 1B (PTP1B), one of the protein tyrosine phosphatases (PTPases), is known to be a negative regulator of insulin signal transduction by dephosphorylating the insulin receptor as well as its substrate, insulin receptor substrates [2]. The PTP1B inhibitors are recognized as potential therapeutic agents for the treatment of type ΙΙ diabetes and obesity [3]. Interestingly, in comparison of our previously discovered hippolides A–H [14], in which only hippolides A and B displayed weak PTP1B inhibitory activity (23.8 and 39.7 μM), Compound **1** and **5**, with IC_50_ values of 5.2 μM and 8.7 μM, are the most potent compounds isolated from marine sponges of the genus *Hippospongia* relevant to PTP1B inhibitory activity. Biogenetically, all the hippolides discovered so far are sesterterpenoid derivatives. The sesterterpenoids are a group of pentaprenyl terpenoids whose structures are derivable from geranylfarnesyl diphosphate [22]. Hypothetically, compounds **1**–**5** could be biosynthetically formed via multiple reactions involving oxidations, decarboxylations, aminations, dehydrations and double bond formations and shifts, *etc.* with the same precursor, acyclic carboxylic sesterterpenoid.

## Supplementary Files

Supplementary File 1 Supplementary Information (PDF, 3204 KB)
