# Supplementary material for: New Hippolide Derivatives with Protein Tyrosine Phosphatase 1B Inhibitory Activity from the Marine Sponge Hippospongia lachne
Source: Mar Drugs. 2014 Jul 8;12(7):4096–109. doi: 10.3390/md12074096 (PMC4113817; doi:10.3390/md12074096)
Supplement: Supplementary File 1 — Supplementary Information (PDF, 3204 KB) [file marinedrugs-12-04096-s001.pdf]

Supplementary Information

S1. Energy Minimization and ECD Calculations.
Figure S1. HRESIMS spectrum of compound **1**.
Figure S2. IR spectrum of compound **1**.
Figure S3. ^{1}H NMR spectrum of compound **1** in $CDCl_3$.
Figure S4. ^{13}C NMR spectrum of compound **1** in $CDCl_3$.
Figure S5. DEPT spectrum of compound **1** in $CDCl_3$.
Figure S6. HSQC spectrum of compound **1** in $CDCl_3$.
Figure S7. ^{1}H-^{1}H COSY spectrum of compound **1** in $CDCl_3$.
Figure S8. HMBC spectrum of compound **1** in $CDCl_3$.
Figure S9. NOESY spectrum of compound **1** in $CDCl_3$.
Figure S10. HRESIMS spectrum of compound **2**.
Figure S11. IR spectrum of compound **2**.
Figure S12. ^{1}H NMR spectrum of compound **2** in $CDCl_3$.
Figure S13. ^{1}H NMR spectrum of compound **2** in DMSO-d_6.
Figure S14. ^{13}C NMR and DEPT spectrum of compound **2** in $CDCl_3$.
Figure S15. HSQC spectrum of compound **2** in $CDCl_3$.
Figure S16. ^{1}H-^{1}H COSY spectrum of compound **2** in $CDCl_3$.
Figure S17. HMBC spectrum of compound **2** in $CDCl_3$.
Figure S18. NOESY spectrum of compound **2** in $CDCl_3$.
Figure S19. Partial NOESY spectrum of compound **2** in $CDCl_3$.
Figure S20. HRESIMS spectrum of compound **3**.
Figure S21. IR spectrum of compound **3**.
Figure S22. ^{1}H NMR spectrum of compound **3** in $CDCl_3$.
Figure S23. ^{1}H NMR spectrum of compound **3** in DMSO-d_6.
Figure S24. ^{13}C NMR and DEPT spectrum of compound **3** in $CDCl_3$.
Figure S25. DEPT spectrum of compound **3** in $CDCl_3$.
Figure S26. HSQC spectrum of compound **3** in $CDCl_3$.
Figure S27. ^{1}H-^{1}H COSY spectrum of compound **3** in $CDCl_3$.
Figure S28. HMBC spectrum of compound **3** in $CDCl_3$.
Figure S29. NOESY spectrum of compound **3** in $CDCl_3$.
Figure S30. HRESIMS spectrum of compound **4**.
Figure S31. IR spectrum of compound **4**.
Figure S32. ^{1}H NMR spectrum of compound **4** in $CDCl_3$.
Figure S33. ^{13}C NMR and DEPT spectrum of compound **4** in $CDCl_3$.
Figure S34. DEPT spectrum of compound **4** in $CDCl_3$.
Figure S35. HSQC spectrum of compound **4** in $CDCl_3$.
Figure S36. ^{1}H-^{1}H COSY spectrum of compound **4** in $CDCl_3$.
Figure S37. HMBC spectrum of compound **4** in $CDCl_3$.
Figure S38. NOESY spectrum of compound **4** in $CDCl_3$.
Figure S39. HRESIMS spectrum of compound **5**.
Figure S40. IR spectrum of compound **5**.

Figure S41. ^{1}H NMR spectrum of compound **5** in $CDCl_3$.
Figure S42. ^{1}H NMR spectrum of compound **5** in DMSO-d_6.
Figure S43. ^{13}C NMR spectrum of compound **5** in $CDCl_3$.
Figure S44. DEPT spectrum of compound **5** in $CDCl_3$.
Figure S45. HSQC spectrum of compound **5** in $CDCl_3$.
Figure S46. ^{1}H-^{1}H COSY spectrum of compound **5** in $CDCl_3$.
Figure S47. HMBC spectrum of compound **5** in $CDCl_3$.
Figure S48. NOESY spectrum of compound **5** in $CDCl_3$.
Figure S49. ^{1}H NMR data of MTPA esters (2*S*, 2*R*, 3*S*, 3*R*).

S1. Energy Minimization and ECD Calculations

The initial conformational distribution search was performed using MMFF94 method overlaid with key correlations observed in the NOESY spectra of **1**. The corresponding minimum geometries were preoptimized at HF/6-31G level in Gaussian 03 program package [1,2], which was further checked by frequency calculation and resulted in no imaginary frequencies. And their minimum geometries were further optimized by DFT calculation B3LYP at 6-31 + g(d) level in the gas phase. The stable conformers obtained were submitted to ECD calculation by TDDFT [b3lyp/aug-cc-pvdz] method under Self-Consistent Reaction Field model of solvent (MeOH). The overall predicted ECD spectra of **1** were subsequently compared with the experimental one.

*S1.1. Energy Minimization of Four Possible Relative Structures of **1** (**1A**–**1D**)*

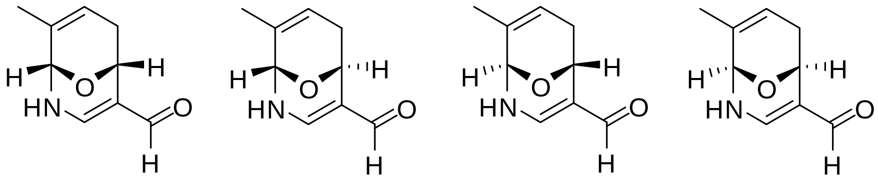

1A **1B** **1C** **1D**

Formula: $\Delta E = (E - E_{1A}) \times 627.51$ kcal/mol [2]

1A

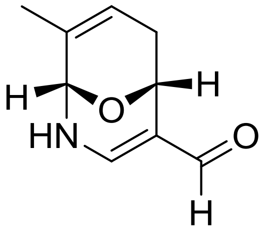

E(RHF/6-31G(d)) = −551.3563 a.u.

ΔE = 0 kcal/mol

Standard orientation:

Center Number	Atomic Number	Atomic Type	Coordinates (Angstroms) X	Y	Z
1	6	0	-3.102171	-0.099256	-0.930399
2	6	0	-1.798273	-0.426787	-0.255432
3	6	0	-1.277377	0.601481	0.733570
4	8	0	-0.319261	0.057862	1.582865
5	6	0	0.741647	-0.526112	0.850402
6	6	0	0.227165	-1.795318	0.176444
7	6	0	-1.099086	-1.523185	-0.483627
8	7	0	-0.733857	1.744730	0.013558
9	6	0	1.291673	0.513891	-0.102040
10	6	0	2.637915	0.374606	-0.644413
11	6	0	0.573753	1.606699	-0.376782
12	8	0	3.356972	-0.558984	-0.440264
13	1	0	2.976650	1.204636	-1.275062
14	1	0	1.504967	-0.769485	1.574830
15	1	0	-2.083543	0.956218	1.360713
16	1	0	-3.029737	0.834941	-1.479812
17	1	0	-3.903737	0.013643	-0.203414
18	1	0	-3.388959	-0.878972	-1.625918
19	1	0	0.953011	-2.147325	-0.548555
20	1	0	0.116056	-2.581577	0.920072
21	1	0	-1.473031	-2.264134	-1.171138
22	1	0	-0.988967	2.647504	0.353125
23	1	0	0.981117	2.424296	-0.946894

1B

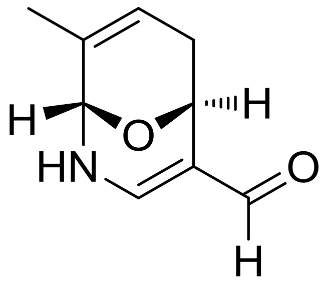

E(RHF/6-31G(d)) = −551.2413 a.u.
ΔE = 72.2 kcal/mol
Standard orientation:

Center	Atomic	Atomic	Coordinates (Angstroms)		
Number	Number	Type	X	Y	Z
1	6	0	3.473705	−0.218837	−0.438751
2	6	0	2.084722	0.233903	−0.080757
3	6	0	1.135059	−0.792785	0.600811
4	8	0	0.189380	−0.016980	1.182020
5	6	0	−0.450188	0.544816	0.013426
6	6	0	0.173358	1.929571	−0.012140
7	6	0	1.657201	1.497217	−0.198855
8	7	0	0.327975	−1.765557	−0.230878
9	6	0	−1.625824	−0.380148	−0.157152
10	6	0	−3.057186	−0.151050	−0.195651
11	6	0	−1.054412	−1.604168	−0.268576
12	8	0	−3.583063	0.918178	−0.104642
13	1	0	−3.660977	−1.056224	−0.332393
14	1	0	0.066627	0.187292	−0.854753
15	1	0	1.679276	−1.364427	1.344142
16	1	0	3.440492	−0.992517	−1.202957
17	1	0	3.988639	−0.640250	0.421801
18	1	0	4.073142	0.598762	−0.820566
19	1	0	−0.134072	2.527461	−0.863597
20	1	0	0.018954	2.501589	0.894354
21	1	0	2.345761	2.252628	−0.539983
22	1	0	0.641932	−2.710380	−0.215747
23	1	0	−1.624747	−2.505729	−0.407308

1C

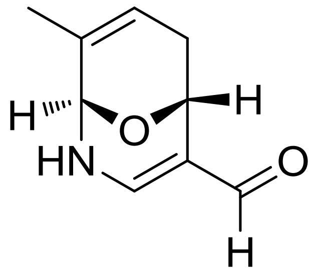

E(RHF/6-31G(d)) = −551.2345 a.u.

ΔE = 70.9 kcal/mol

Standard orientation:

Center Number	Atomic Number	Atomic Type	Coordinates (Angstroms)		
			X	Y	Z
1	6	0	−3.589795	0.245618	−0.204881
2	6	0	−2.165260	−0.210188	−0.202053
3	6	0	−0.907013	0.619393	−0.007103
4	8	0	−0.348234	0.090703	1.187403
5	6	0	0.651525	−0.691763	0.626756
6	6	0	−0.164112	−1.771791	−0.219065
7	6	0	−1.690721	−1.453934	−0.301720
8	7	0	−0.351136	1.936015	−0.158793
9	6	0	1.537007	0.401717	−0.038906
10	6	0	2.956043	0.153922	−0.299352
11	6	0	1.039860	1.650345	−0.195034
12	8	0	3.478952	−0.912519	−0.172552
13	1	0	3.537994	1.020699	−0.630466
14	1	0	1.226537	−1.174355	1.404237
15	1	0	−0.397099	0.208240	−0.848281
16	1	0	−3.764042	0.978754	−0.987595
17	1	0	−3.846337	0.715106	0.741247
18	1	0	−4.263334	−0.588229	−0.364557
19	1	0	0.245380	−1.878972	−1.220162
20	1	0	−0.051057	−2.739291	0.257694
21	1	0	−2.352507	−2.299270	−0.385857
22	1	0	−0.605149	2.636377	0.511627
23	1	0	1.676621	2.483443	−0.436992

1D

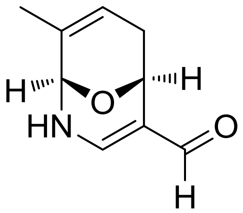

Energetically could not exist

S1.2. Standard Orientation of Two Enantiomers of ***1A*** *for ECD Calculation*

Optimized structure of **1a**

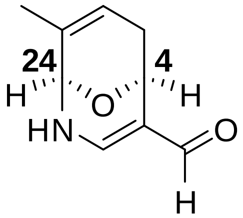

1a (4*S*, 24*R*)

Cartesian coordinate of **1a** optimized:
Standard orientation:

Center Number	Atomic Number	Atomic Type	Coordinates (Angstroms)		
			X	Y	Z
1	6	0	3.149957	−0.092595	0.900662
2	6	0	1.831941	−0.418435	0.251470
3	6	0	1.289853	0.607391	−0.737823
4	8	0	0.318826	0.053978	−1.602059
5	6	0	−0.750079	−0.544368	−0.830420
6	6	0	−0.213168	−1.805750	−0.140902
7	6	0	1.123753	−1.528448	0.495959
8	7	0	0.709516	1.752775	−0.012310
9	6	0	−1.317427	0.503220	0.107874
10	6	0	−2.661483	0.379048	0.629509
11	6	0	−0.589665	1.626778	0.376800
12	8	0	−3.413888	−0.569962	0.421567
13	1	0	−3.002564	1.229117	1.262961
14	1	0	−1.514927	−0.811558	−1.563754
15	1	0	2.091837	0.989067	−1.377028
16	1	0	3.092828	0.845144	1.470528
17	1	0	3.946859	0.032966	0.153304
18	1	0	3.457226	−0.885290	1.590639
19	1	0	−0.938695	−2.149436	0.606426
20	1	0	−0.117369	−2.616906	−0.878655
21	1	0	1.520930	−2.277754	1.180550
22	1	0	1.090493	2.678056	−0.160992
23	1	0	−1.014826	2.463982	0.927344

Optimized structure of the enantiomer **1b**

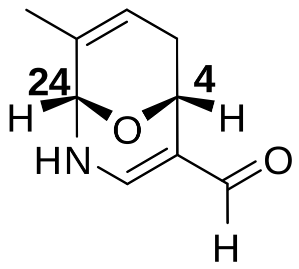

1b (4*R*, 24*S*)

Cartesian coordinate of the enantiomer **1b** optimized:
Standard orientation:

Center Number	Atomic Number	Atomic Type	Coordinates (Angstroms)		
			X	Y	Z
1	6	0	−3.149957	−0.092595	−0.900662
2	6	0	−1.831941	−0.418435	−0.251470
3	6	0	−1.289853	0.607391	0.737823
4	8	0	−0.318826	0.053978	1.602059
5	6	0	0.750079	−0.544368	0.830420
6	6	0	0.21316	−1.805750	0.140902
7	6	0	−1.123753	−1.528448	−0.495959
8	7	0	−0.709516	1.752775	0.012310
9	6	0	1.317427	0.503220	−0.107874
10	6	0	2.661483	0.379048	−0.629509
11	6	0	0.589665	1.626778	−0.376800
12	8	0	3.413888	−0.569962	−0.421567
13	1	0	3.002564	1.229117	−1.262961
14	1	0	1.514927	−0.811558	1.563754
15	1	0	−2.091837	0.989067	1.377028
16	1	0	−3.092828	0.845144	−1.470528
17	1	0	−3.946859	0.032966	−0.153304
18	1	0	−3.457226	−0.885290	−1.590639
19	1	0	0.938695	−2.149436	−0.606426
20	1	0	0.117369	−2.616906	0.878655
21	1	0	−1.520930	−2.277754	−1.180550
22	1	0	−1.090493	2.678056	0.160992
23	1	0	1.014826	2.463982	−0.927344

Figure S1. HRESIMS spectrum of compound **1**.

Elemental Composition Report

Page 1

Tolerance = 10.0 PPM / DBE: min = -1.5, max = 50.0
Selected filters: None

Monoisotopic Mass, Even Electron Ions
50 formula(e) evaluated with 1 results within limits (up to 50 closest results for each mass)
Elements Used:
C: 5-50 H: 5-100 N: 1-1 O: 1-18 Na: 1-1

SIPI
PSJ-B6 M.W=369
WQ10180H3 25 (0.880) AM (Med,6, Ar,5000.0,403.13,0.70); Sm (SG, 2x3.00); Cm (23:64)

Q-Tof micro
YA019

12-May-2010,14:30:05
0.00000000
TOF MS ES+
7.70e4

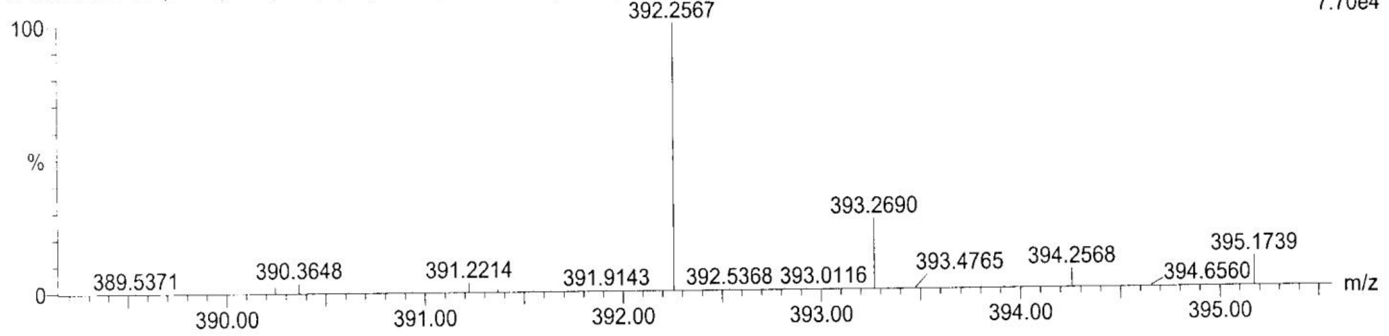

Minimum:	30.00				-1.5		
Maximum:	100.00		5.0	10.0	50.0		
Mass	RA	Calc. Mass	mDa	PPM	DBE	i-FIT	Formula
392.2567	100.00	392.2565	0.2	0.5	7.5	451.4	C24 H35 N O2 Na

Figure S2. IR spectrum of compound **1**.

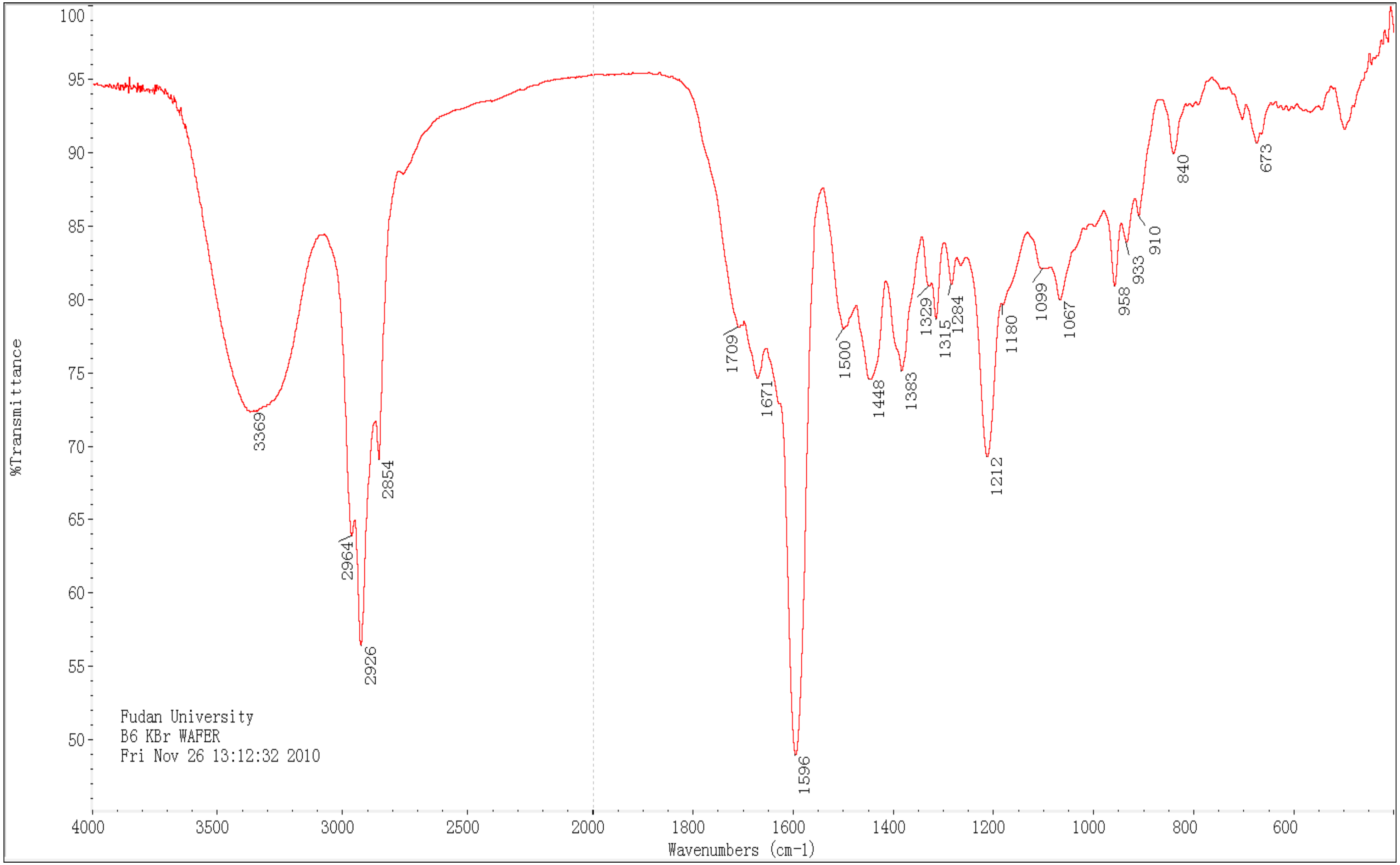

Figure S3. ^{1}H NMR spectrum of compound **1** in $CDCl_3$.

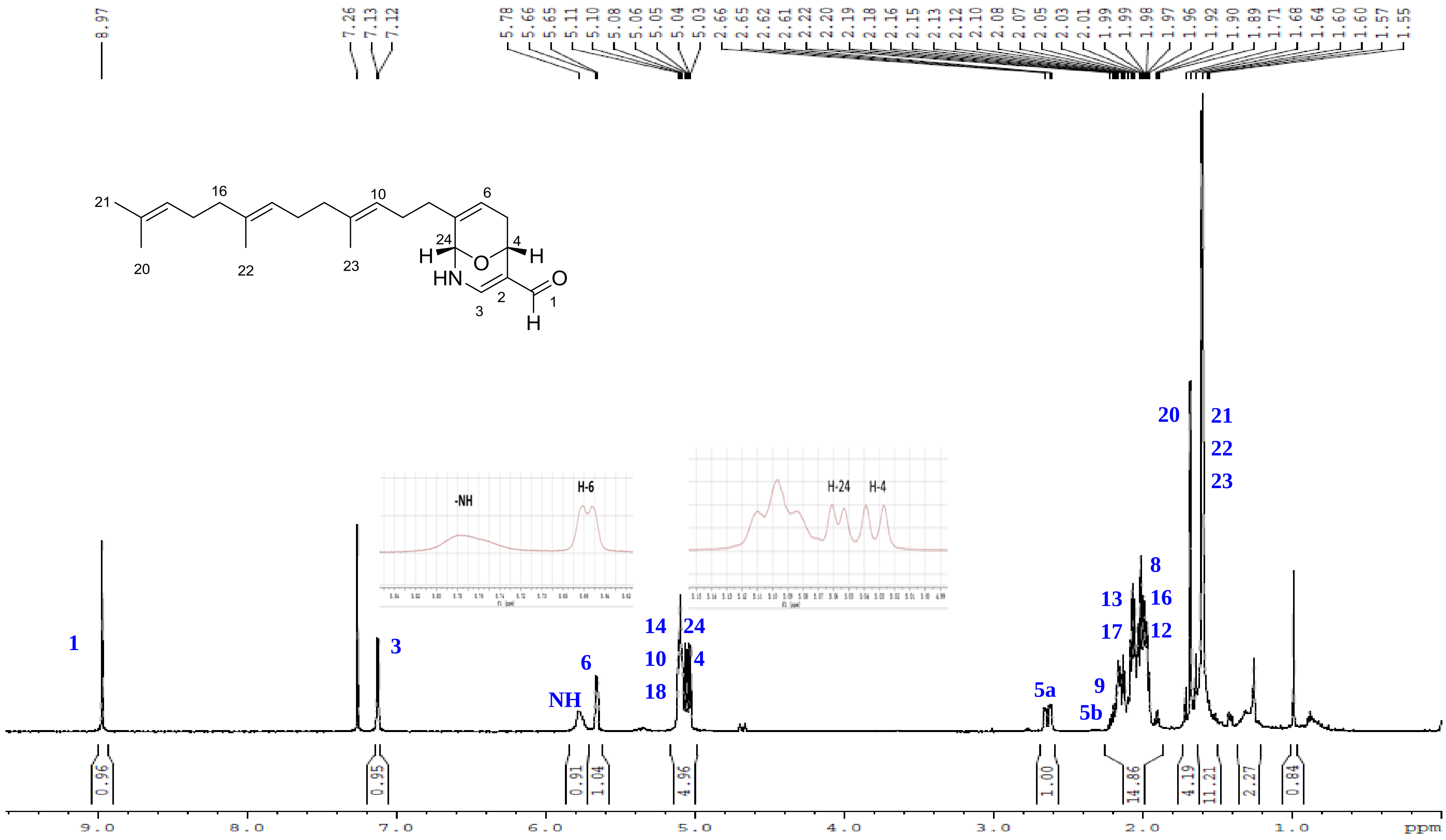

Figure S4. ^{13}C NMR spectrum of compound **1** in $CDCl_3$.

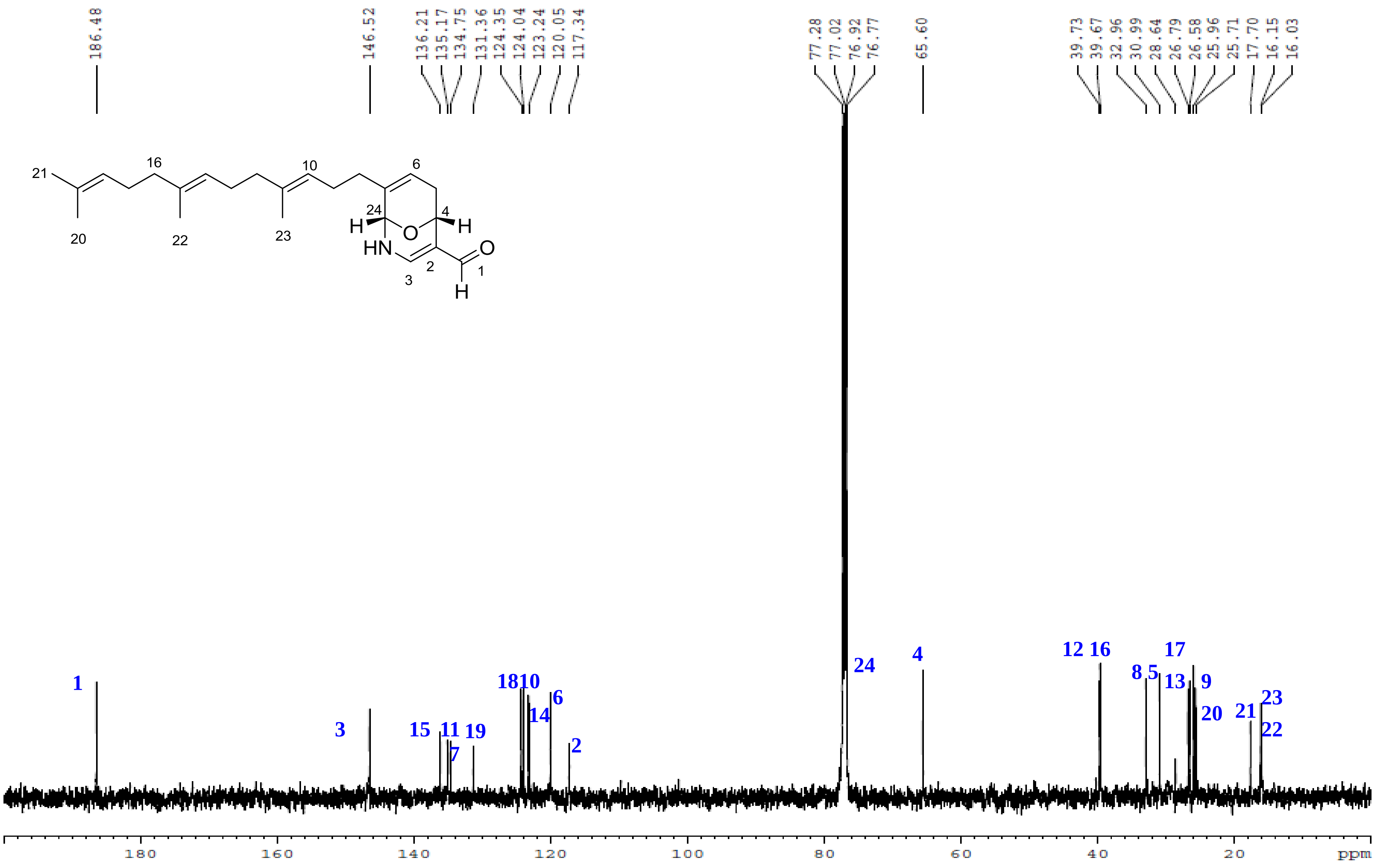

Figure S5. DEPT spectrum of compound **1** in $CDCl_3$.

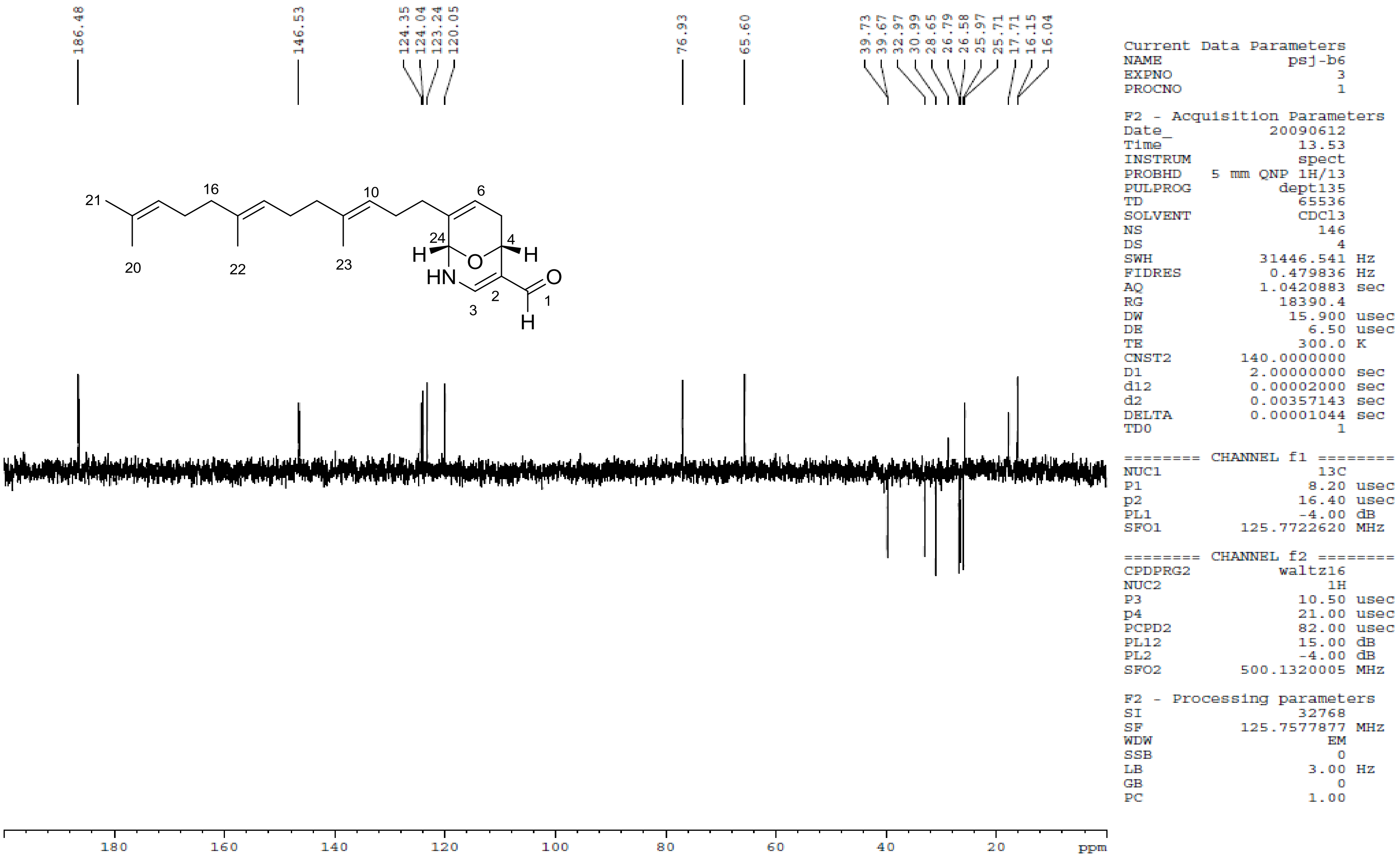

Figure S6. HSQC spectrum of compound **1** in $CDCl_3$.

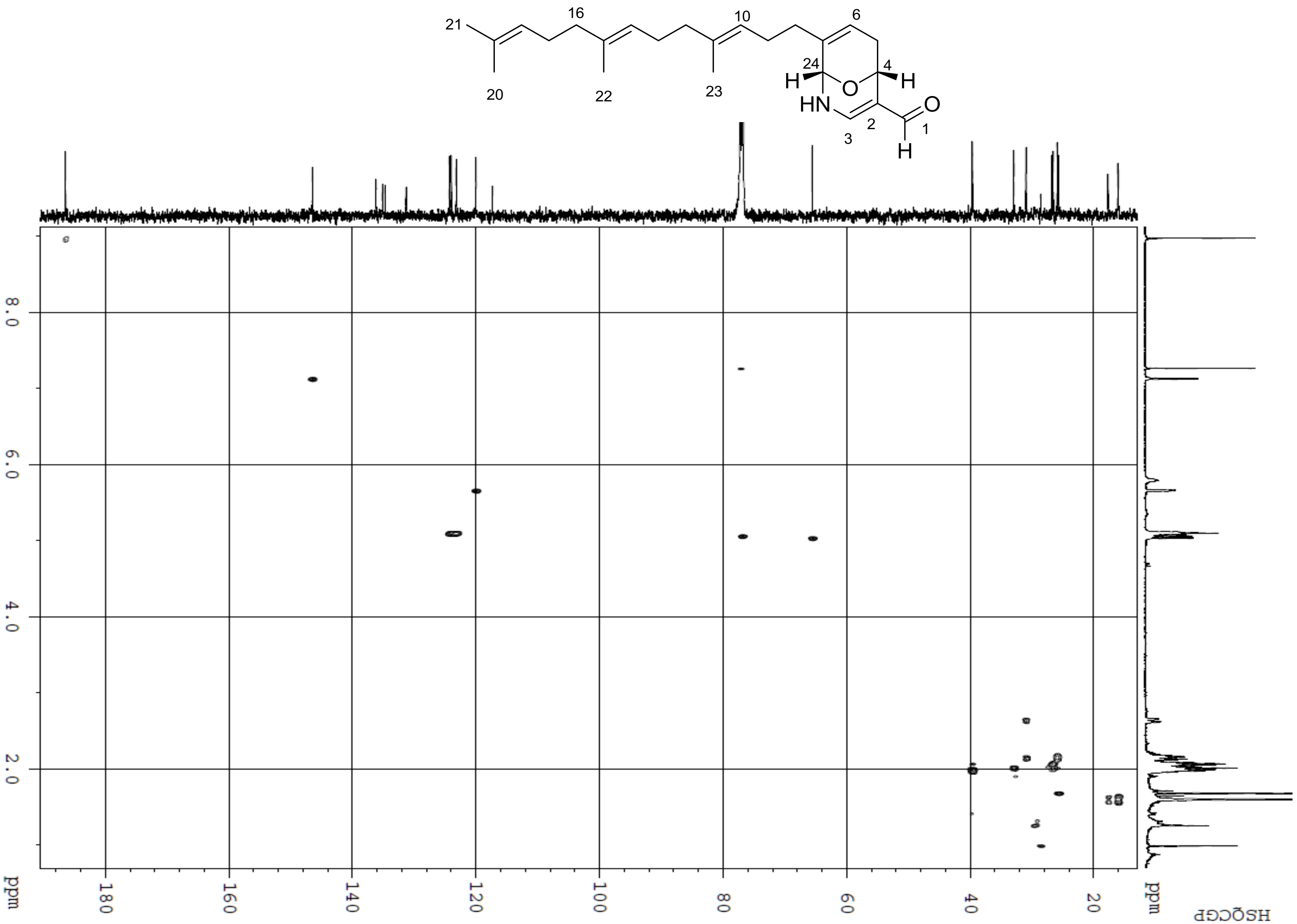

Figure S7. ^{1}H–^{1}H COSY spectrum of compound **1** in $CDCl_3$.

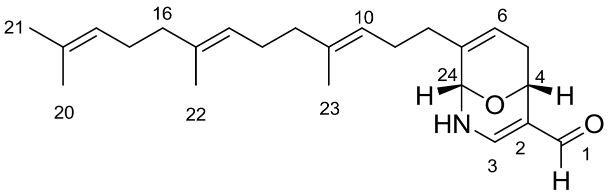

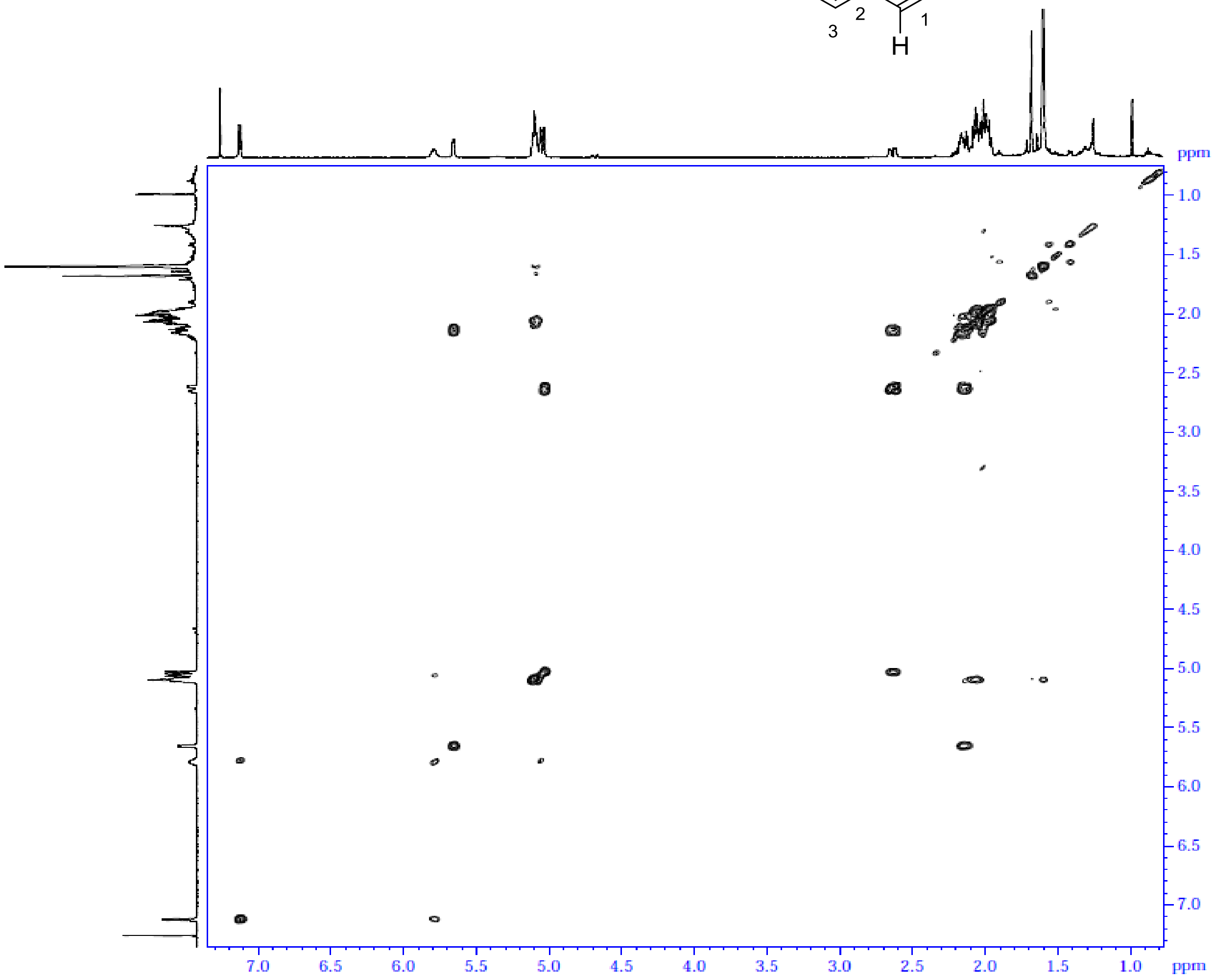

Figure S8. HMBC spectrum of compound **1** in $CDCl_3$.

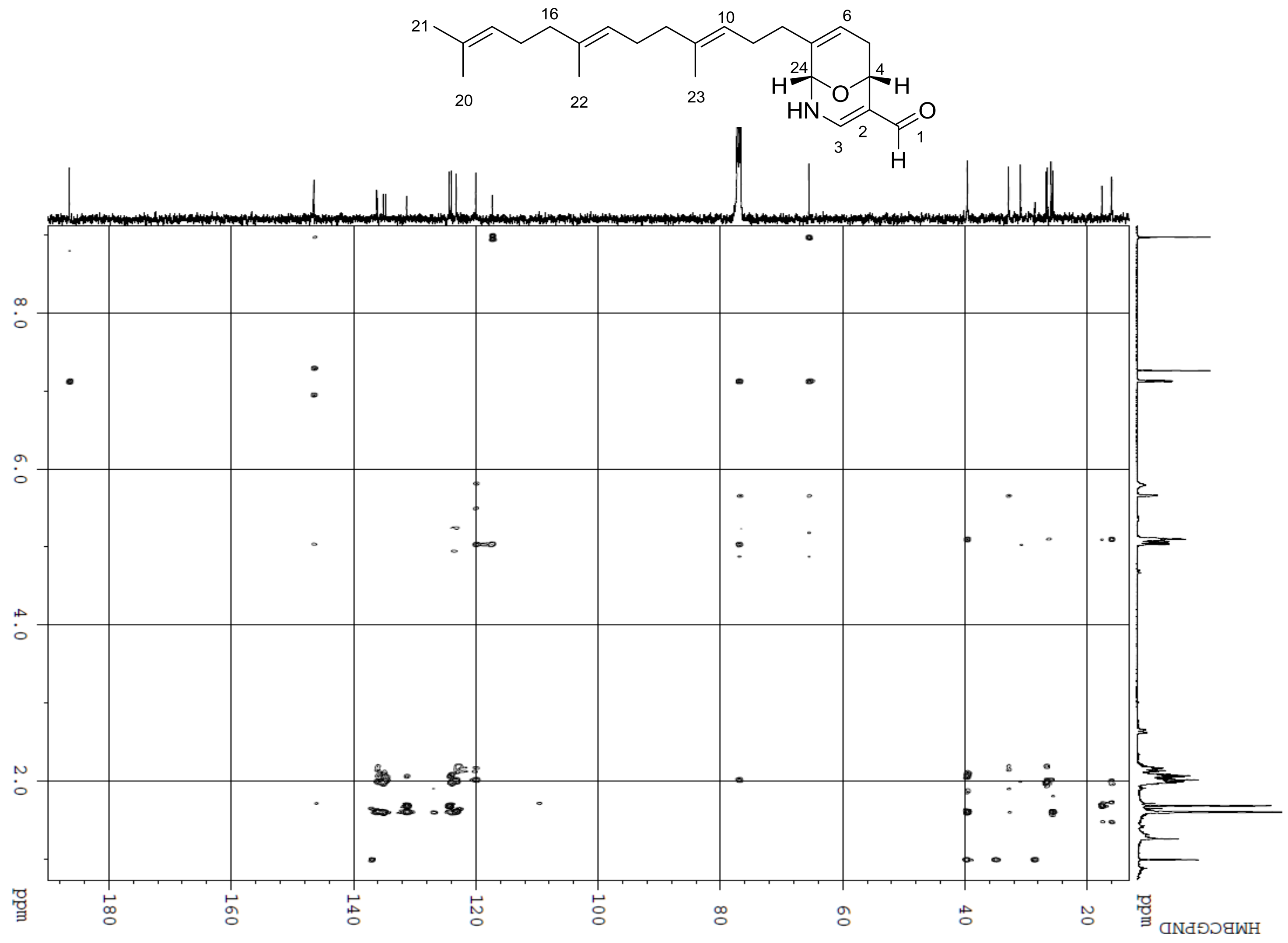

Figure S9. NOESY spectrum of compound **1** in $CDCl_3$.

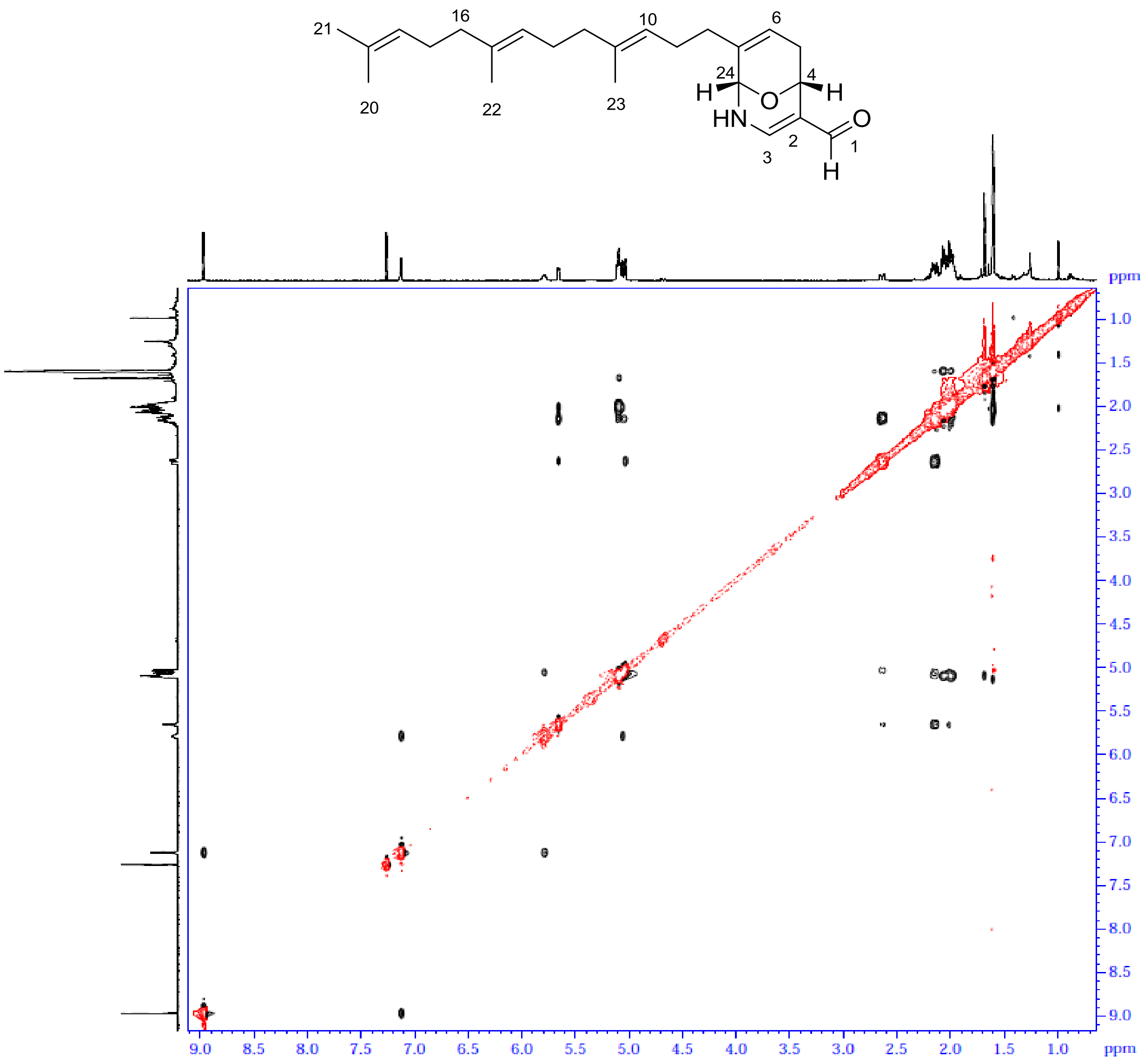

Figure S10. HRESIMS spectrum of compound **2**.

Elemental Composition Report **Page 1**

Tolerance = 10.0 PPM / DBE: min = -1.5, max = 50.0
Selected filters: None

Monoisotopic Mass, Even Electron Ions
34 formula(e) evaluated with 1 results within limits (up to 50 closest results for each mass)
Elements Used:
C: 5-30 H: 5-42 N: 0-1 O: 1-6 Na: 1-1

SIPI Q-Tof micro 10-May-2010,15:32:08
PSJ-B84 M.W=415 YA019 0.00000000
WQ10172H 30 (1.057) AM (Cen,6, 80.00, Ar,5000.0,447.03,0.70); Sm (SG, 2x3.00); Cm (25:42) TOF MS ES+
9.11e4

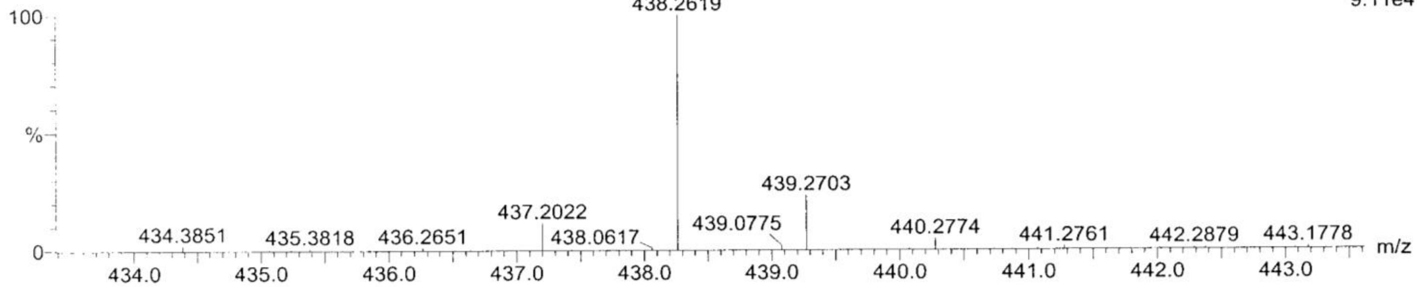

Minimum:	25.00				-1.5		
Maximum:	100.00		5.0	10.0	50.0		
Mass	RA	Calc. Mass	mDa	PPM	DBE	i-FIT	Formula
438.2619	100.00	438.2620	-0.1	-0.2	7.5	487.0	C25 H37 N O4 Na

Figure S11. IR spectrum of compound **2**.

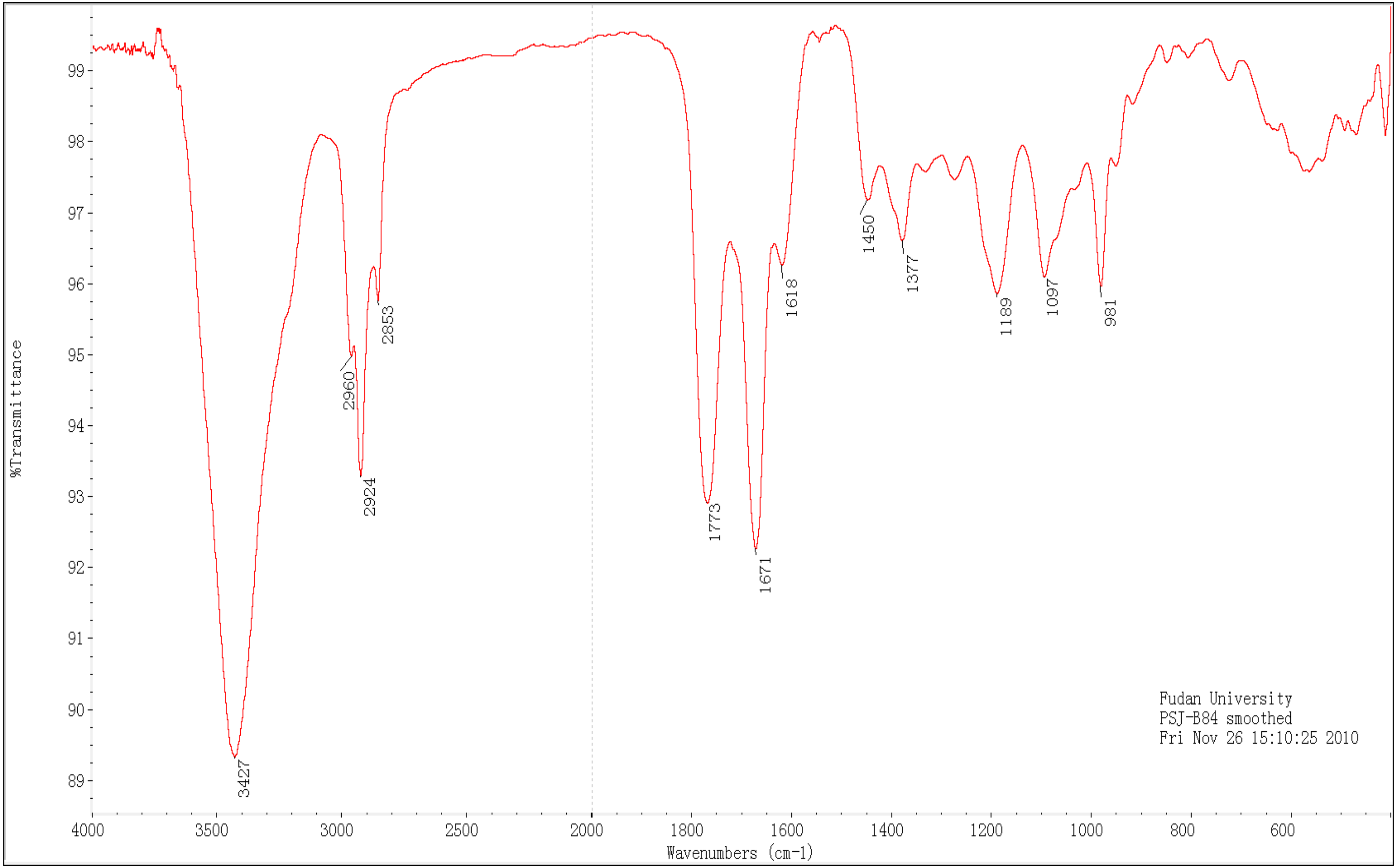

Figure S12. ^{1}H NMR spectrum of compound **2** in $CDCl_3$.

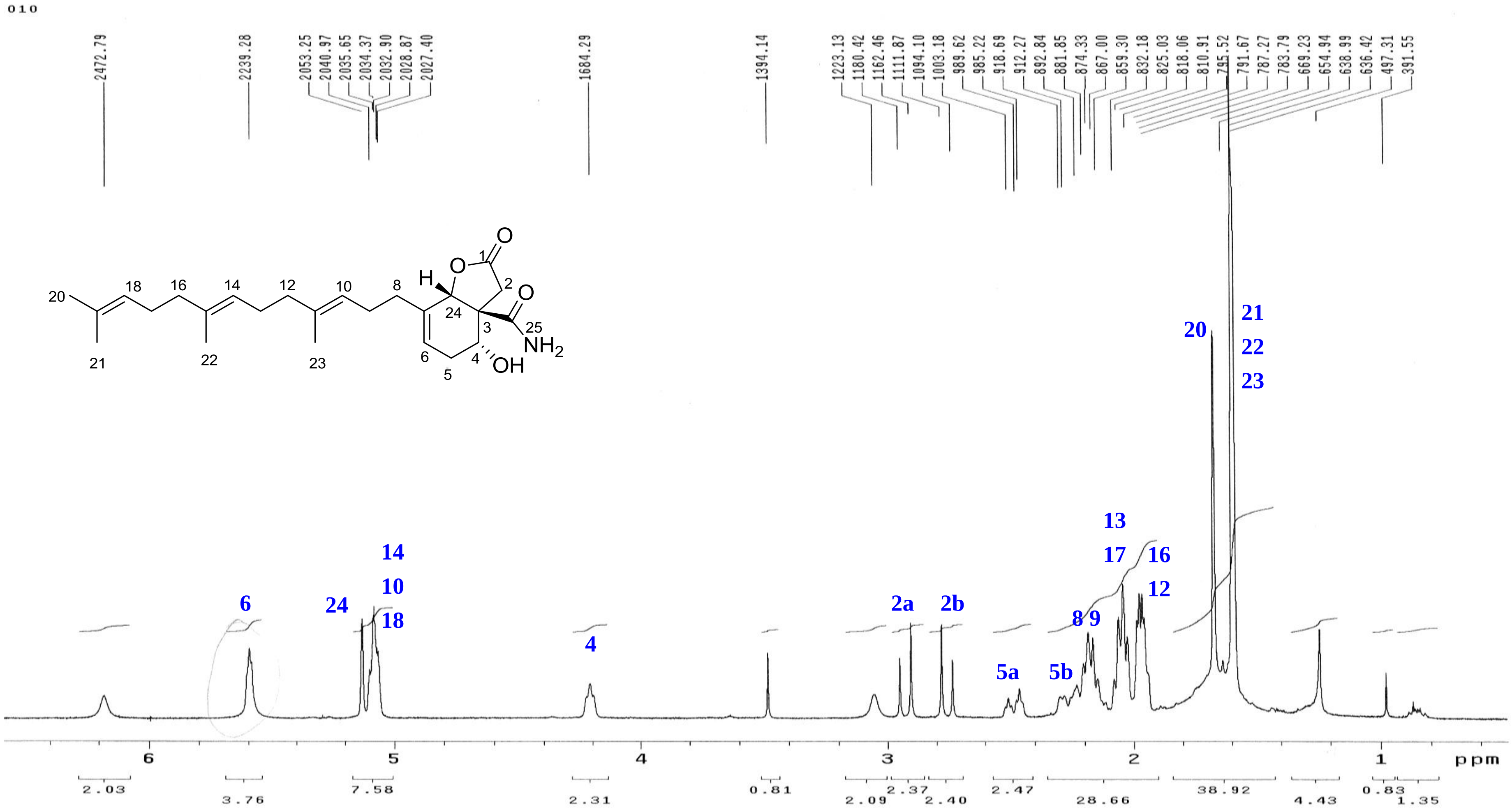

Figure S13. ^{1}H NMR spectrum of compound **2** in DMSO.

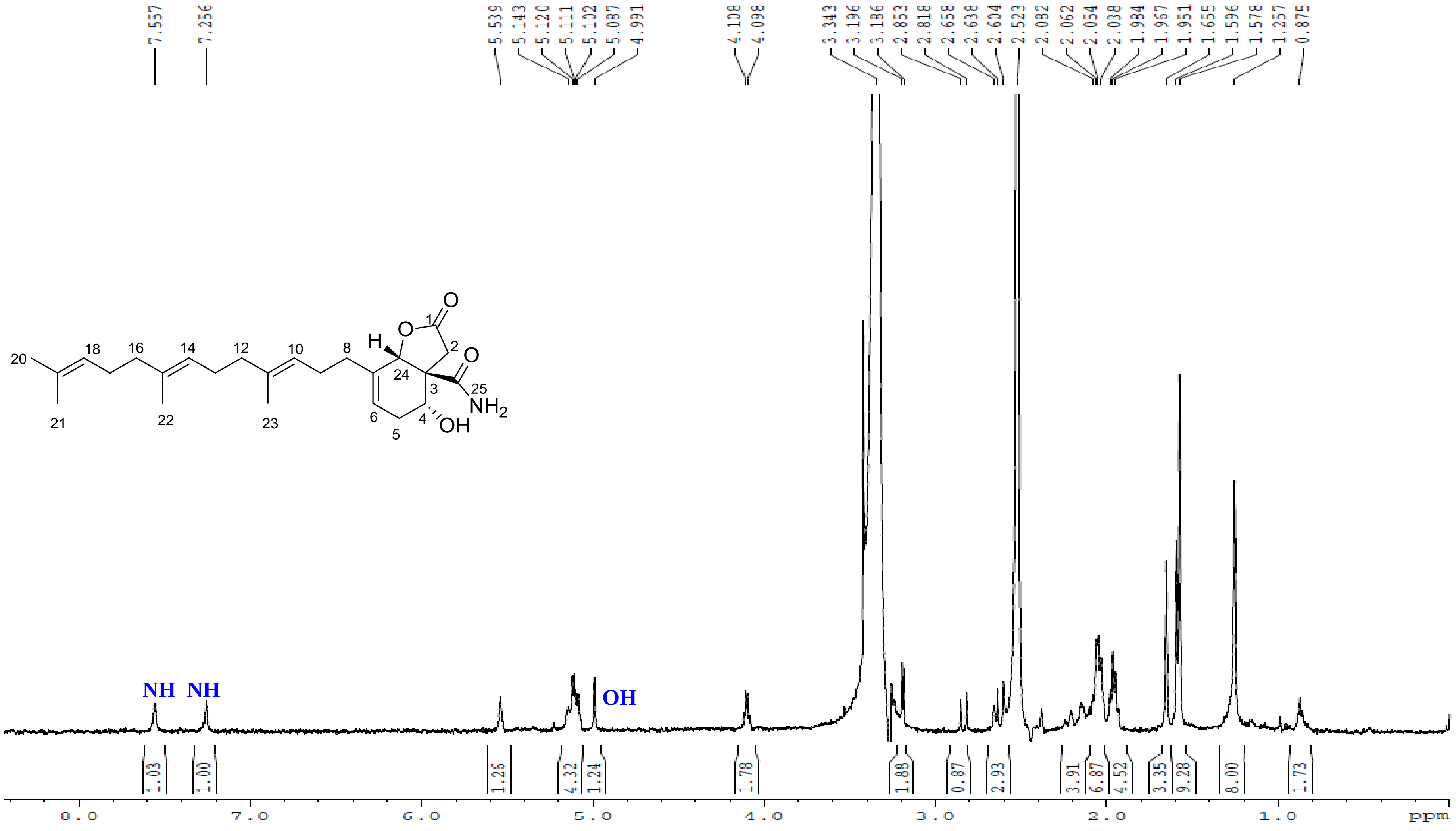

Figure S14. ^{13}C NMR and DEPT spectrum of compound **2** in $CDCl_3$.

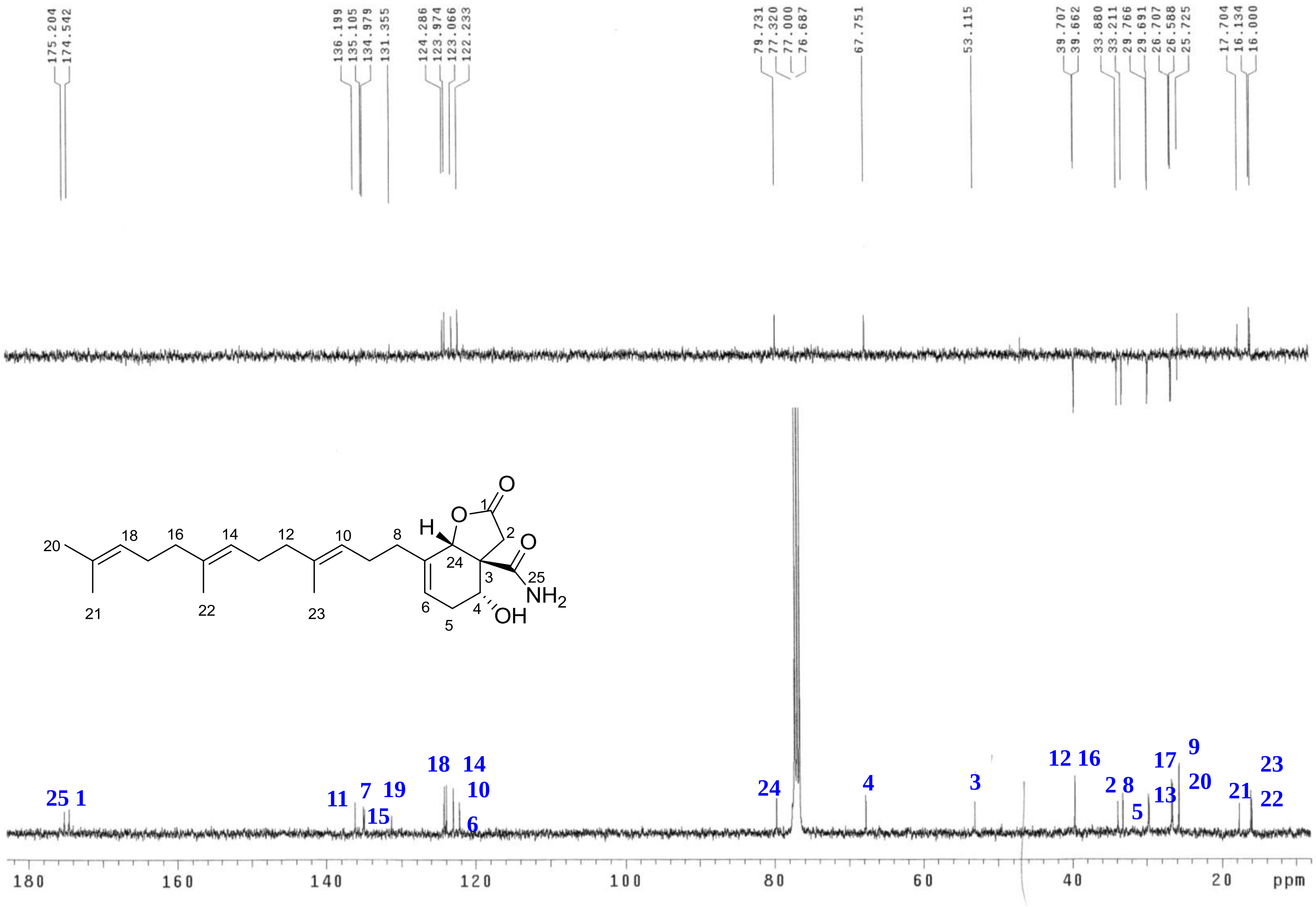

Figure S15. HSQC spectrum of compound **2** in $CDCl_3$.

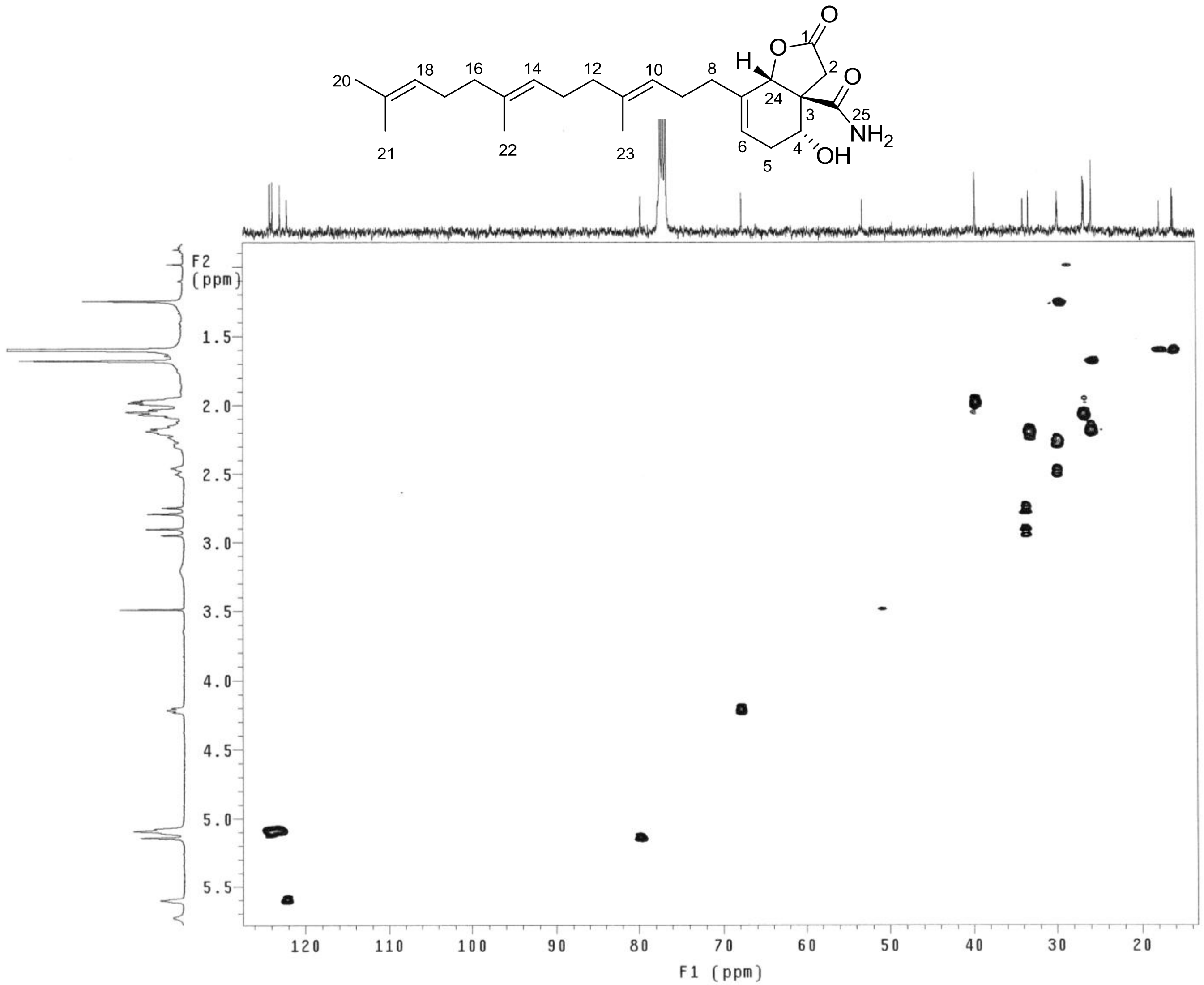

Figure S16. ^{1}H–^{1}H COSY spectrum of compound **2** in $CDCl_3$.

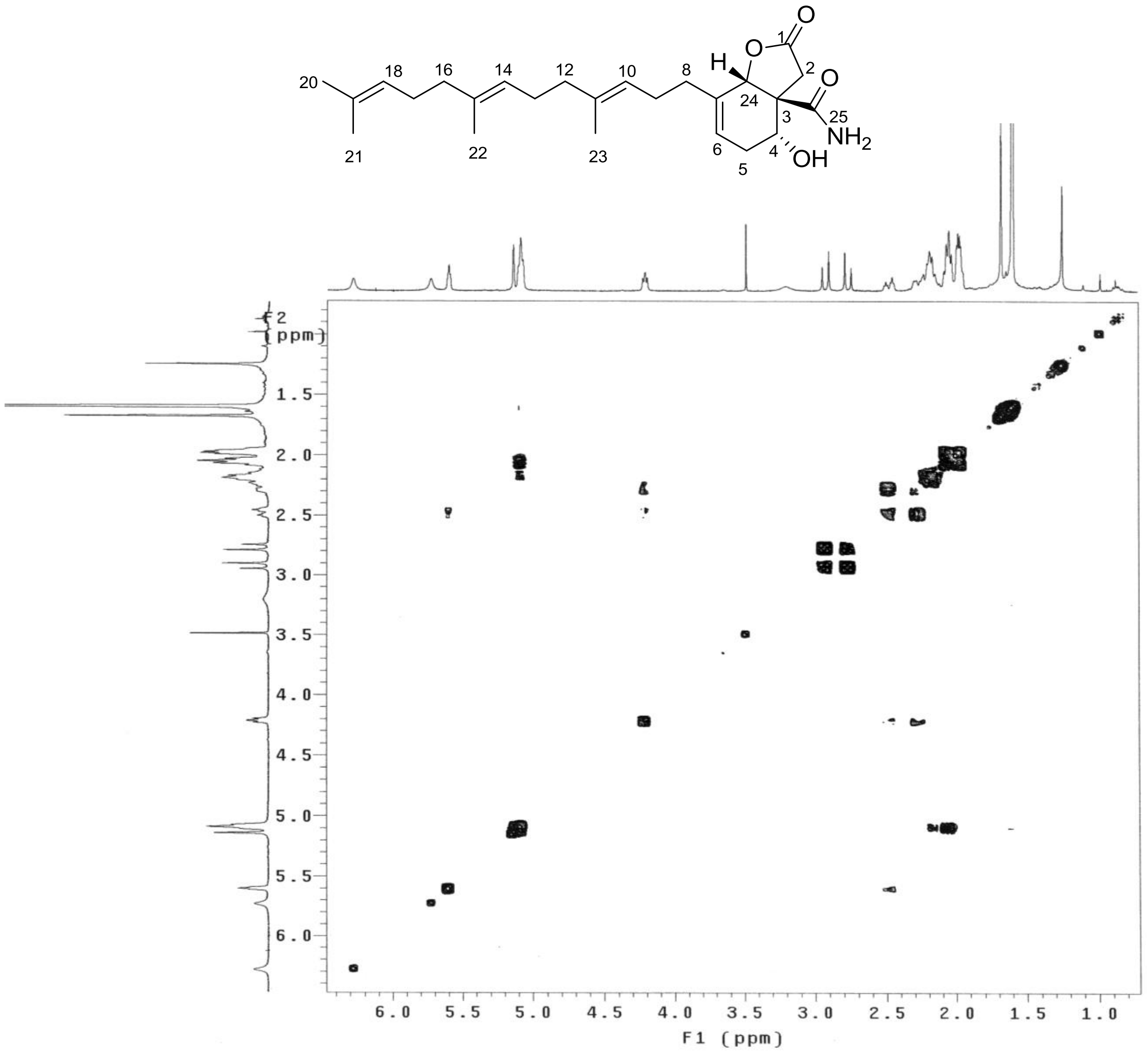

Figure S17. HMBC spectrum of compound **2** in $CDCl_3$.

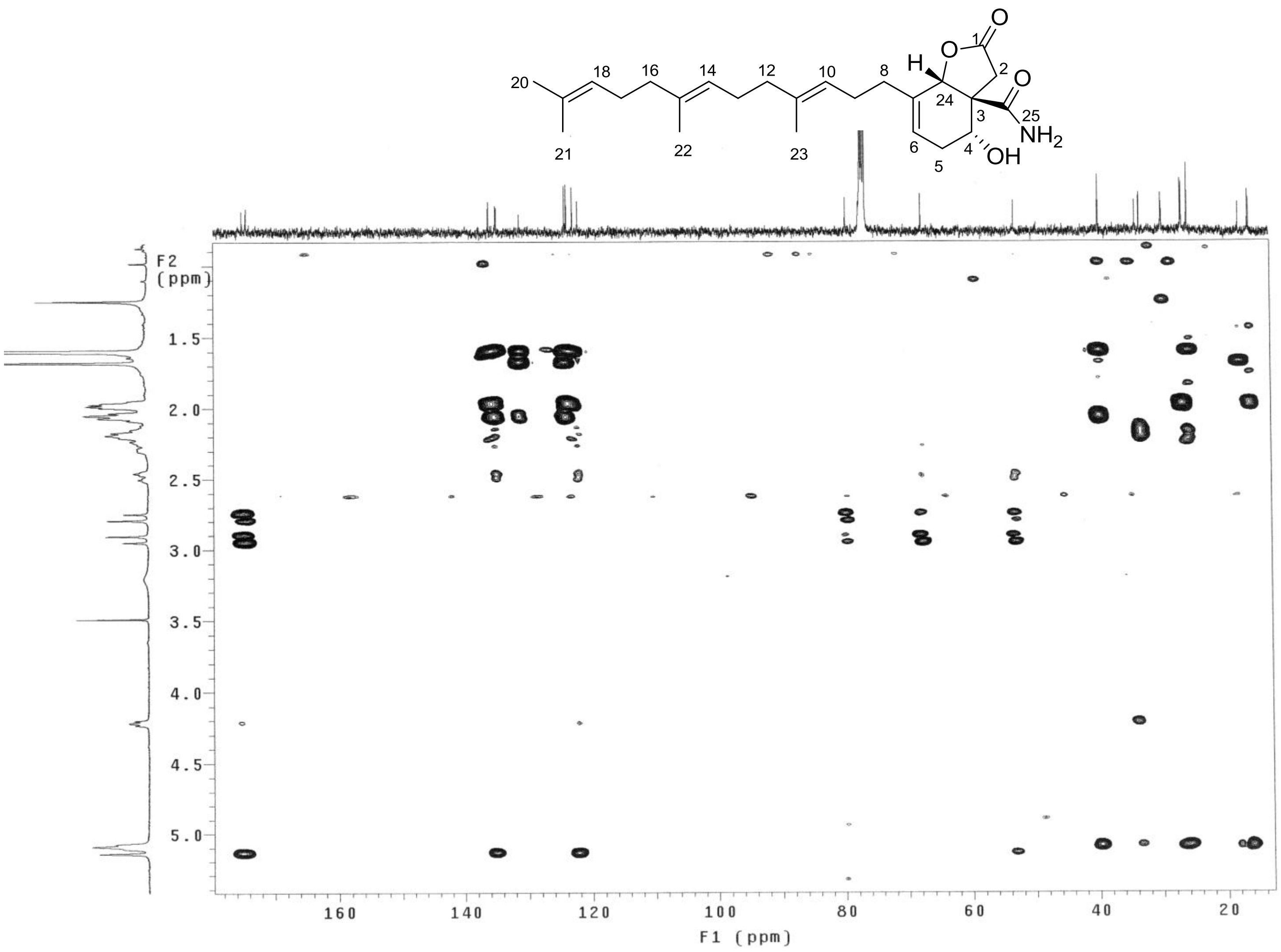

Figure S18. NOESY spectrum of compound **2** in $CDCl_3$.

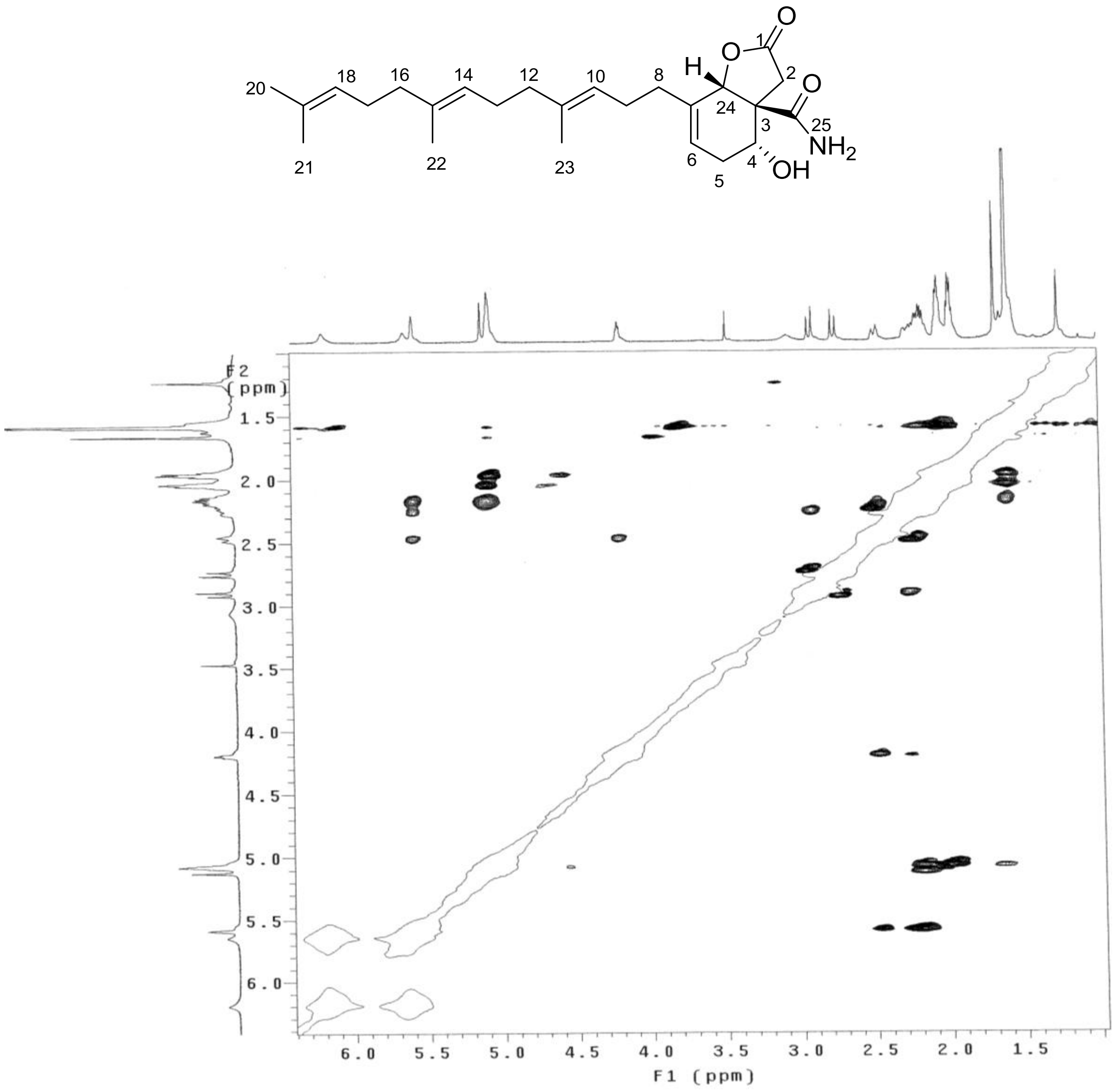

Figure S19. Partial NOESY spectrum of compound **2** in $CDCl_3$.

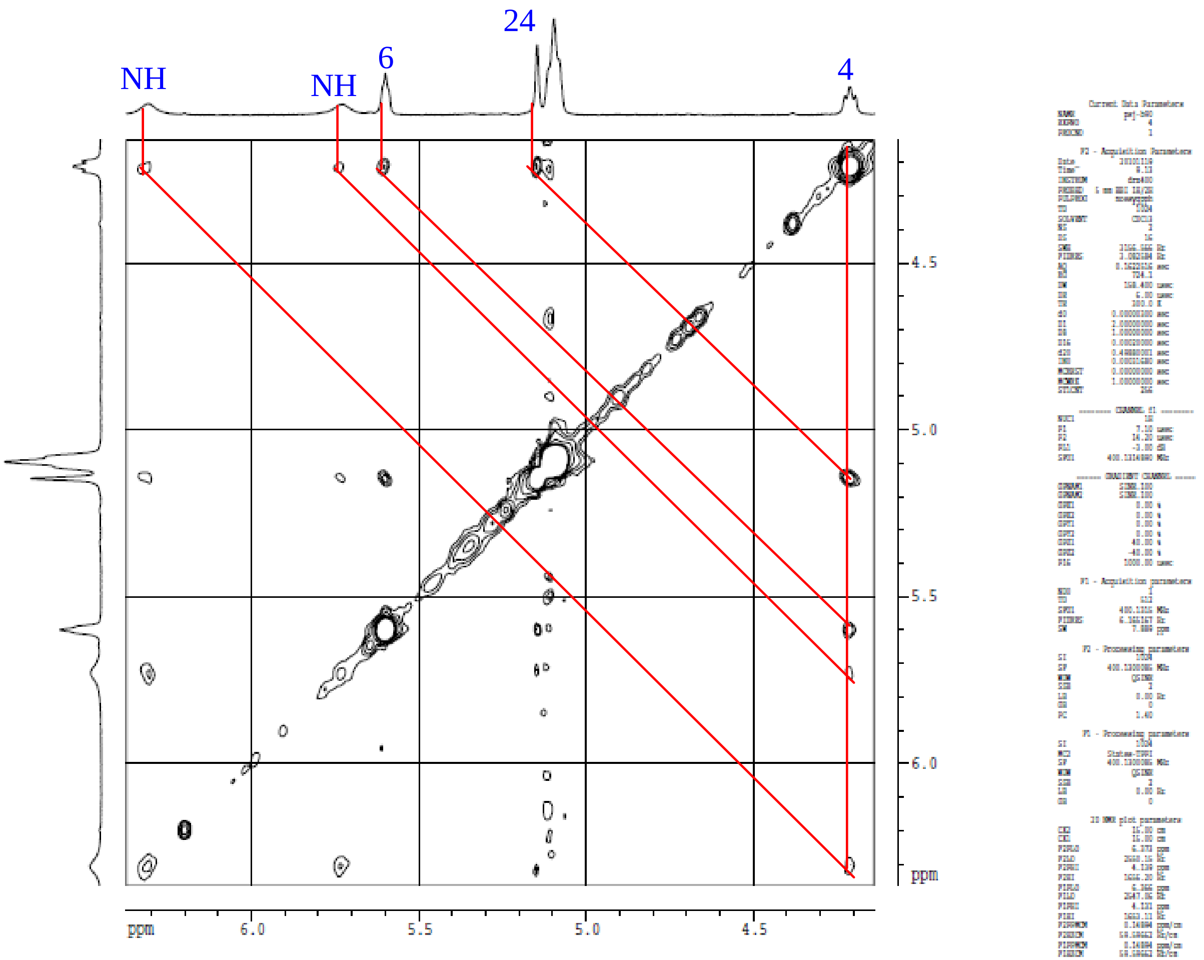

Figure S20. HRESIMS spectrum of compound **3**.

Elemental Composition Report **Page 1**

Tolerance = 20.0 PPM / DBE: min = -1.5, max = 50.0
Selected filters: None

Monoisotopic Mass, Even Electron Ions
20 formula(e) evaluated with 1 results within limits (up to 50 closest results for each mass)
Elements Used:
C: 5-27 H: 10-50 N: 1-3 O: 3-4 Na: 1-1

SIPI Q-Tof micro 04-Oct-2010,11:47:44
PSJ-B10 M.W=415 YA019 0.00000000
WQ10348H1 4 (0.138) AM (Cen,6, 80.00, Ar,5000.0,447.03,1.00); Sm (SG, 2x3.00); Cm (1:22) TOF MS ES+
1.63e4

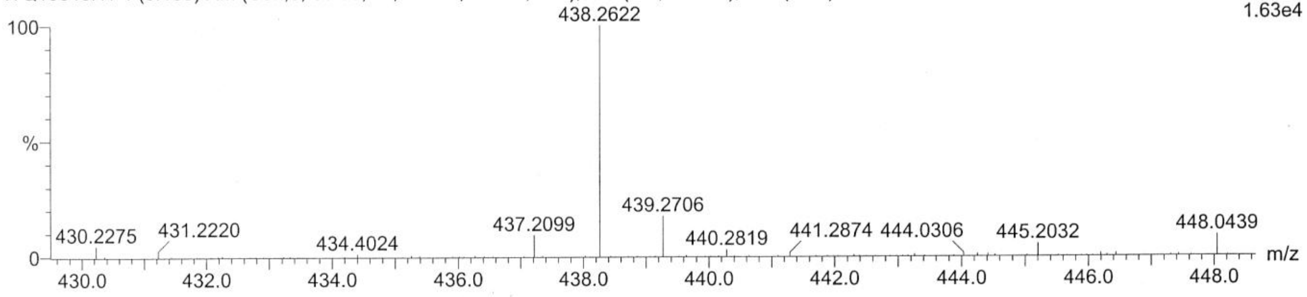

Minimum:	50.00				-1.5		
Maximum:	100.00		5.0	20.0	50.0		
Mass	RA	Calc. Mass	mDa	PPM	DBE	i-FIT	Formula
438.2622	100.00	438.2620	0.2	0.5	7.5	464.9	C25 H37 N O4 Na

Figure S21. IR spectrum of compound **3**.

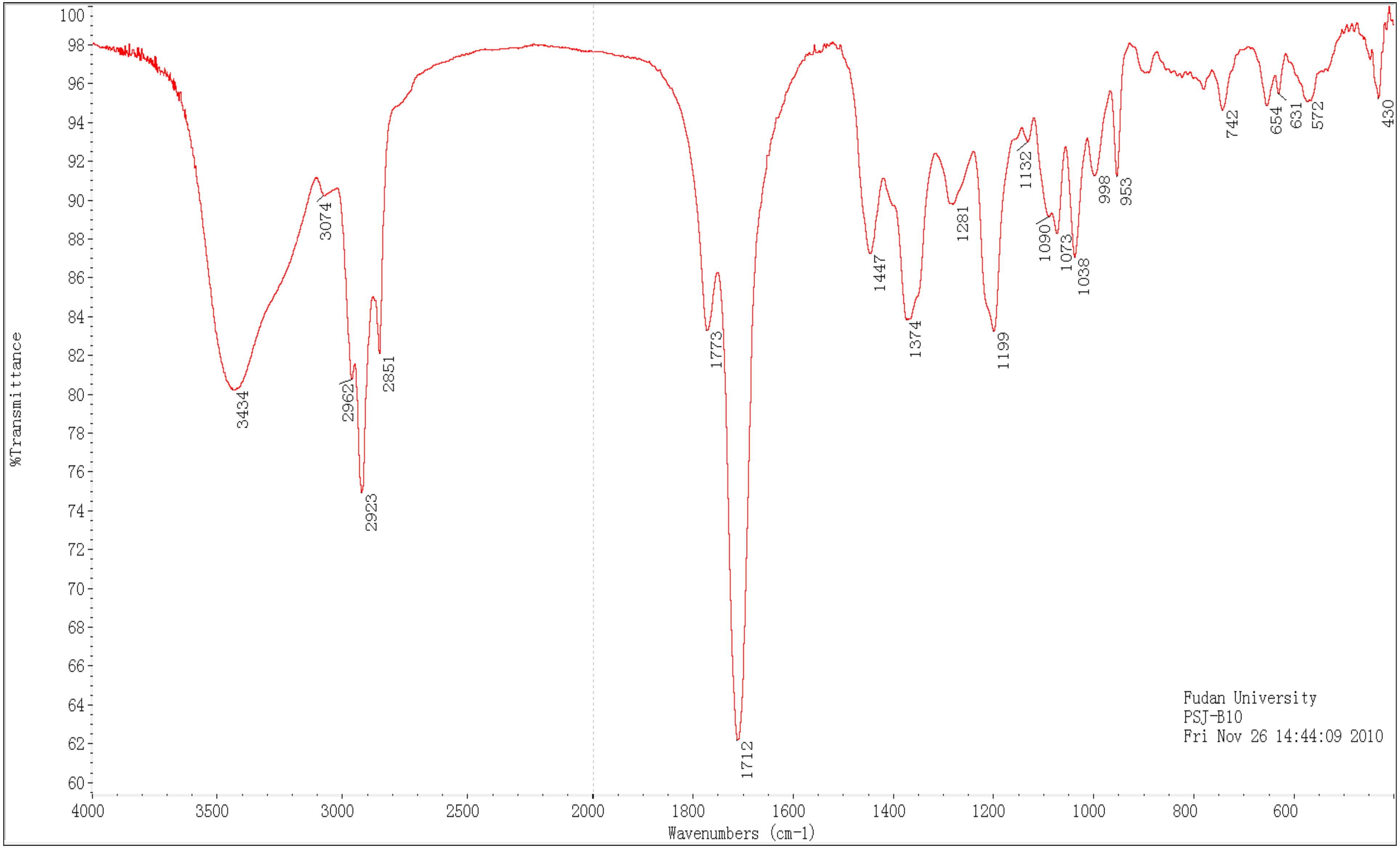

Figure S22. ^{1}H NMR spectrum of compound **3** in $CDCl_3$.

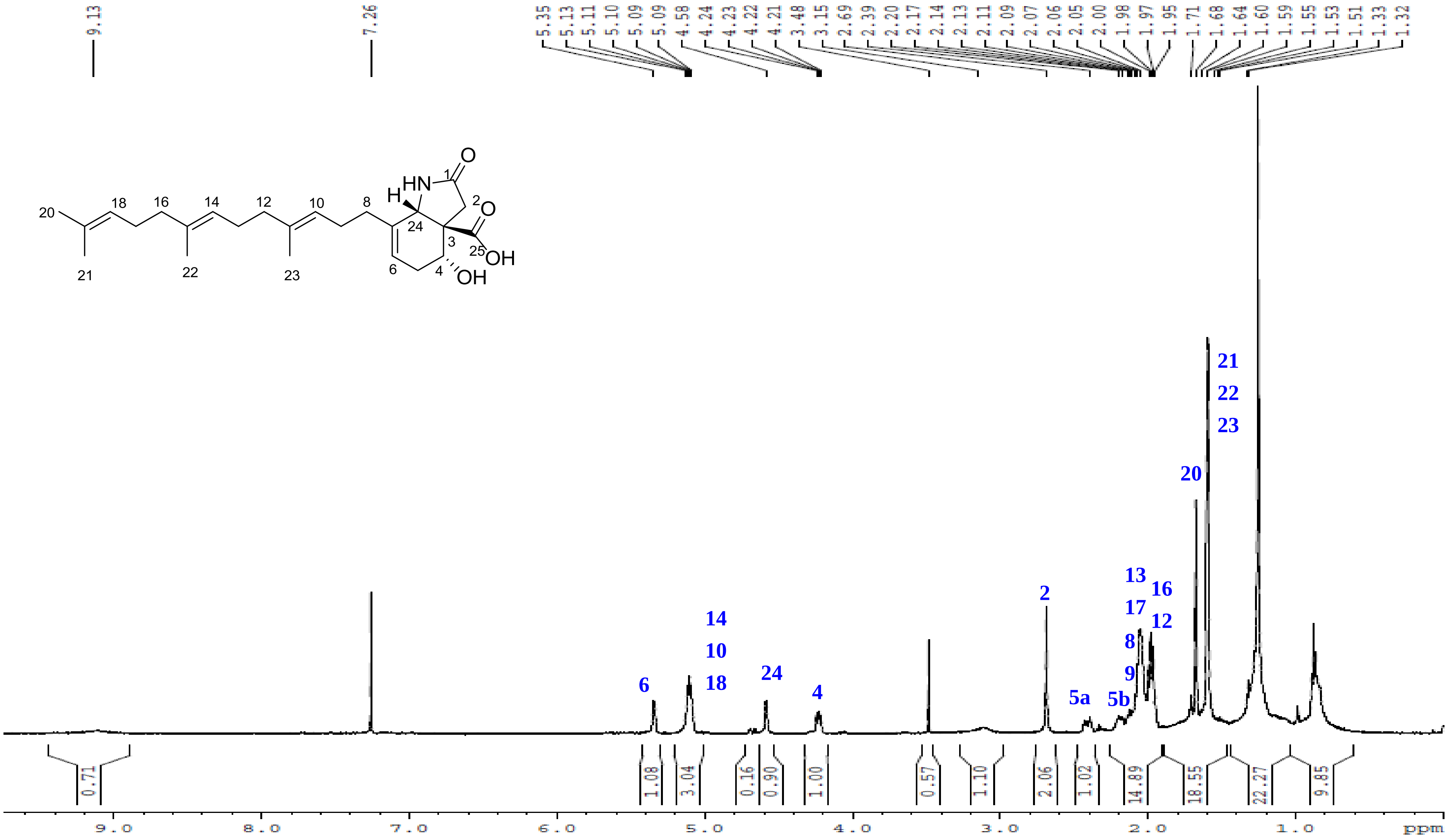

Figure S23. ^{1}H NMR spectrum of compound **3** in DMSO.

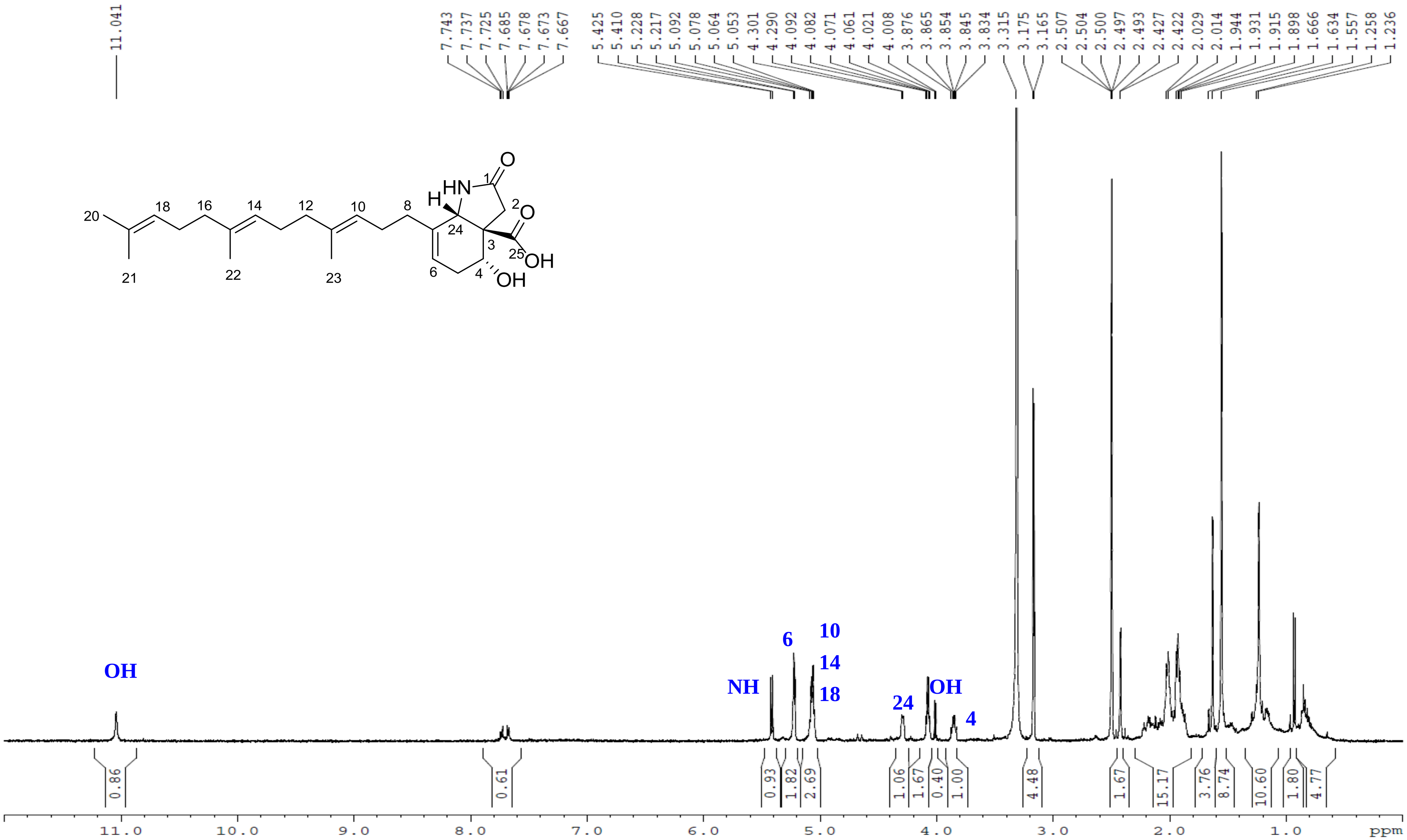

Figure S24. ^{13}C NMR spectrum of compound **3** in $CDCl_3$.

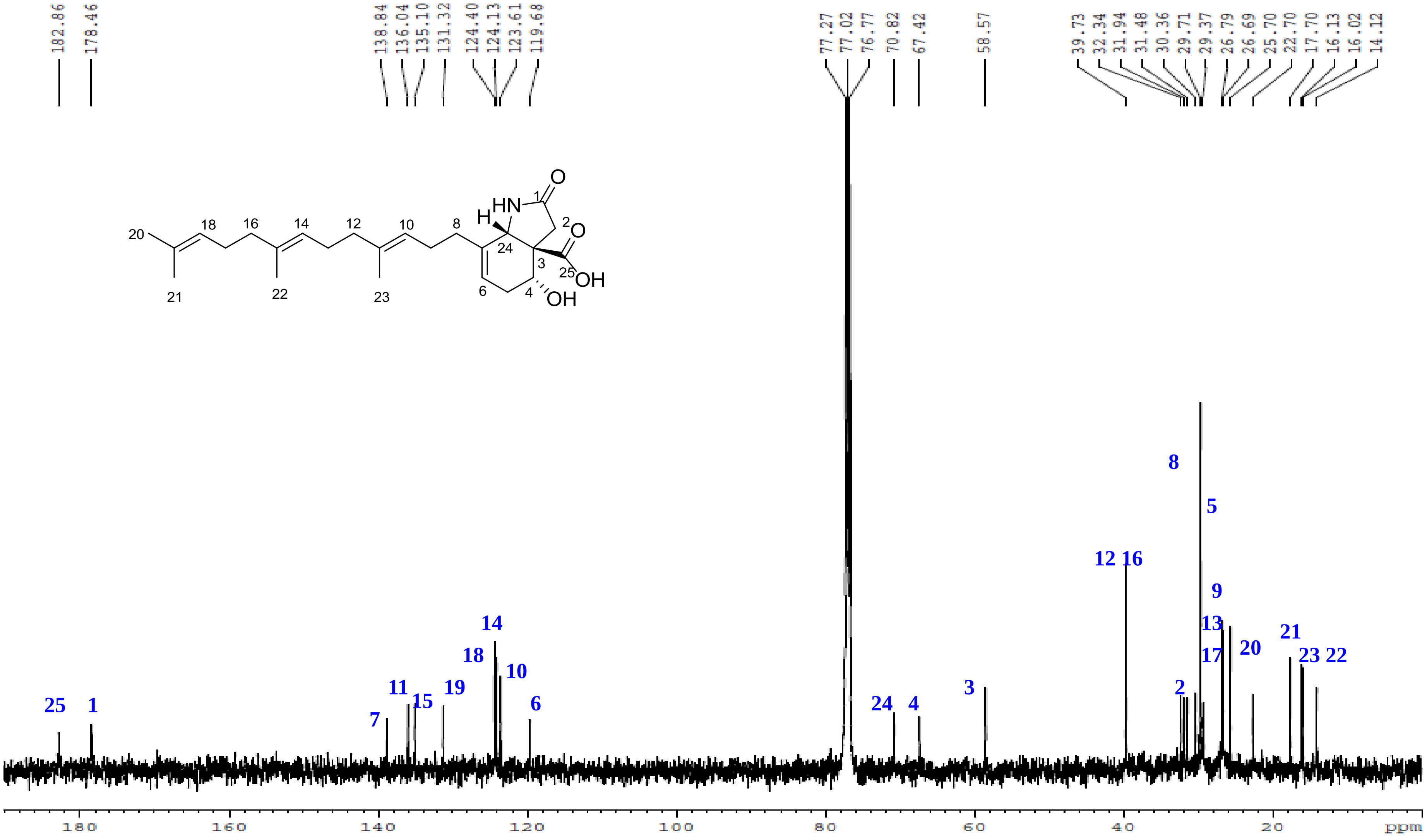

Figure S25. DEPT spectrum of compound **3** in $CDCl_3$.

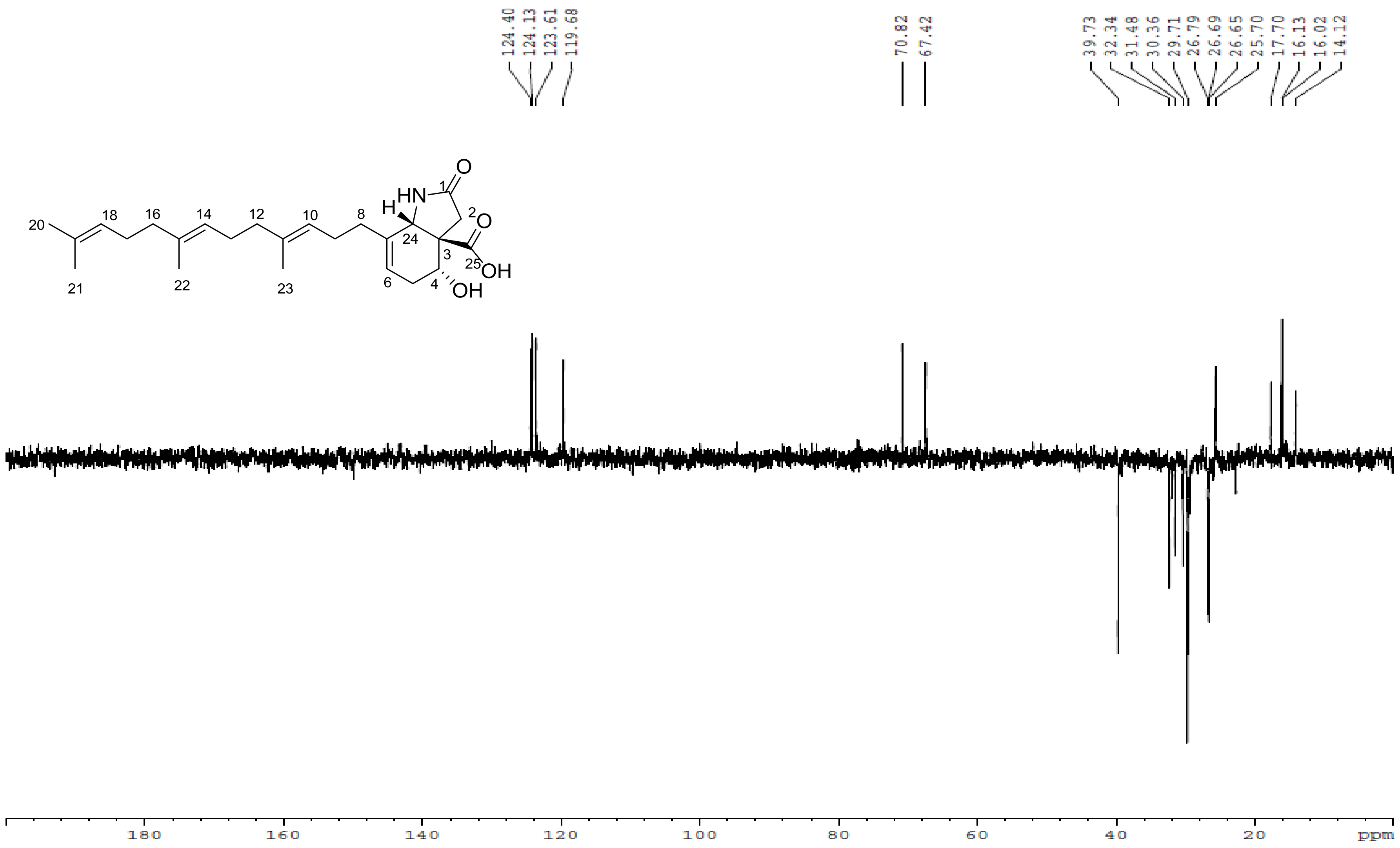

Figure S26. HSQC spectrum of compound **3** in $CDCl_3$.

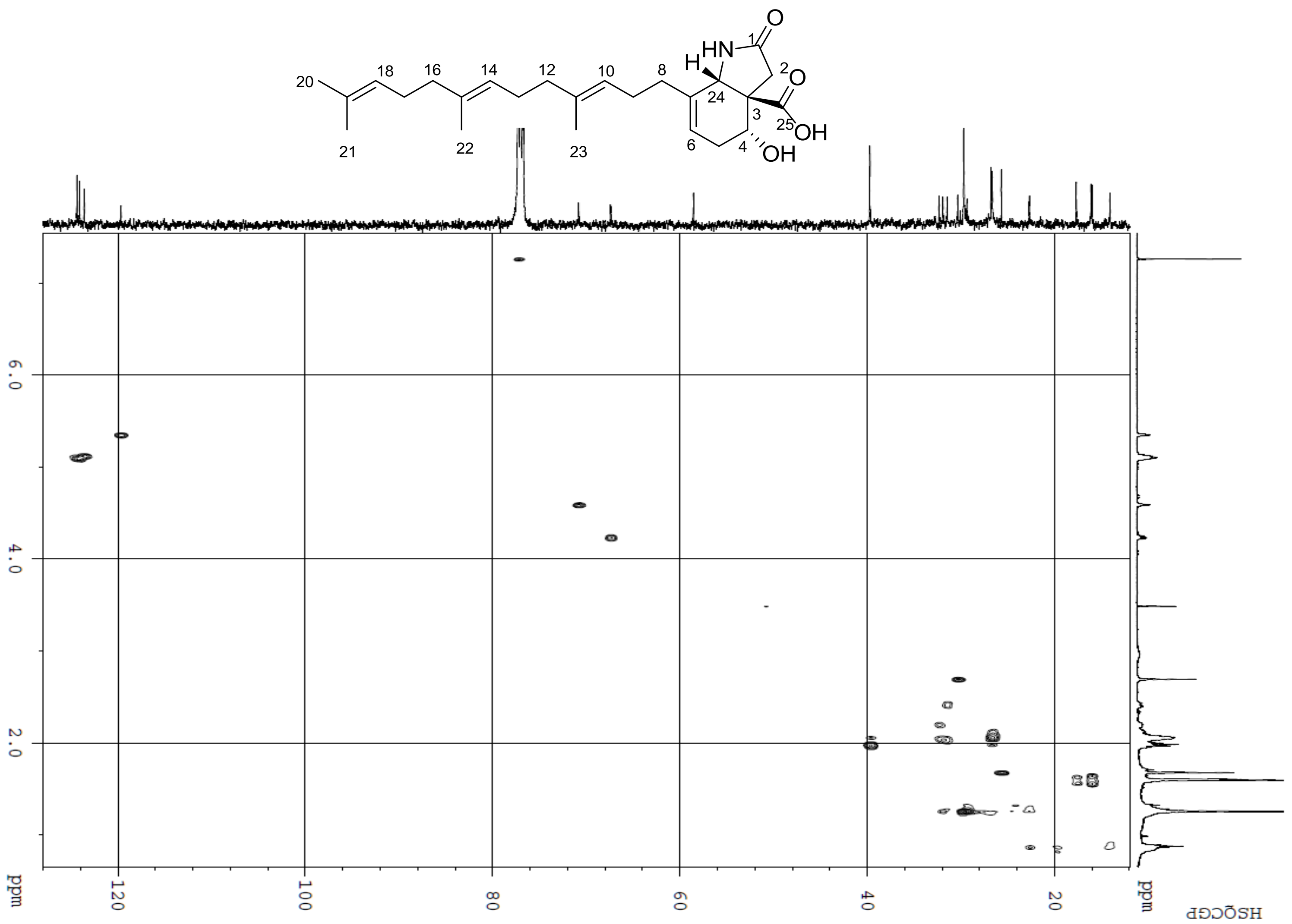

Figure S27. ^{1}H–^{1}H COSY spectrum of compound **3** in $CDCl_3$.

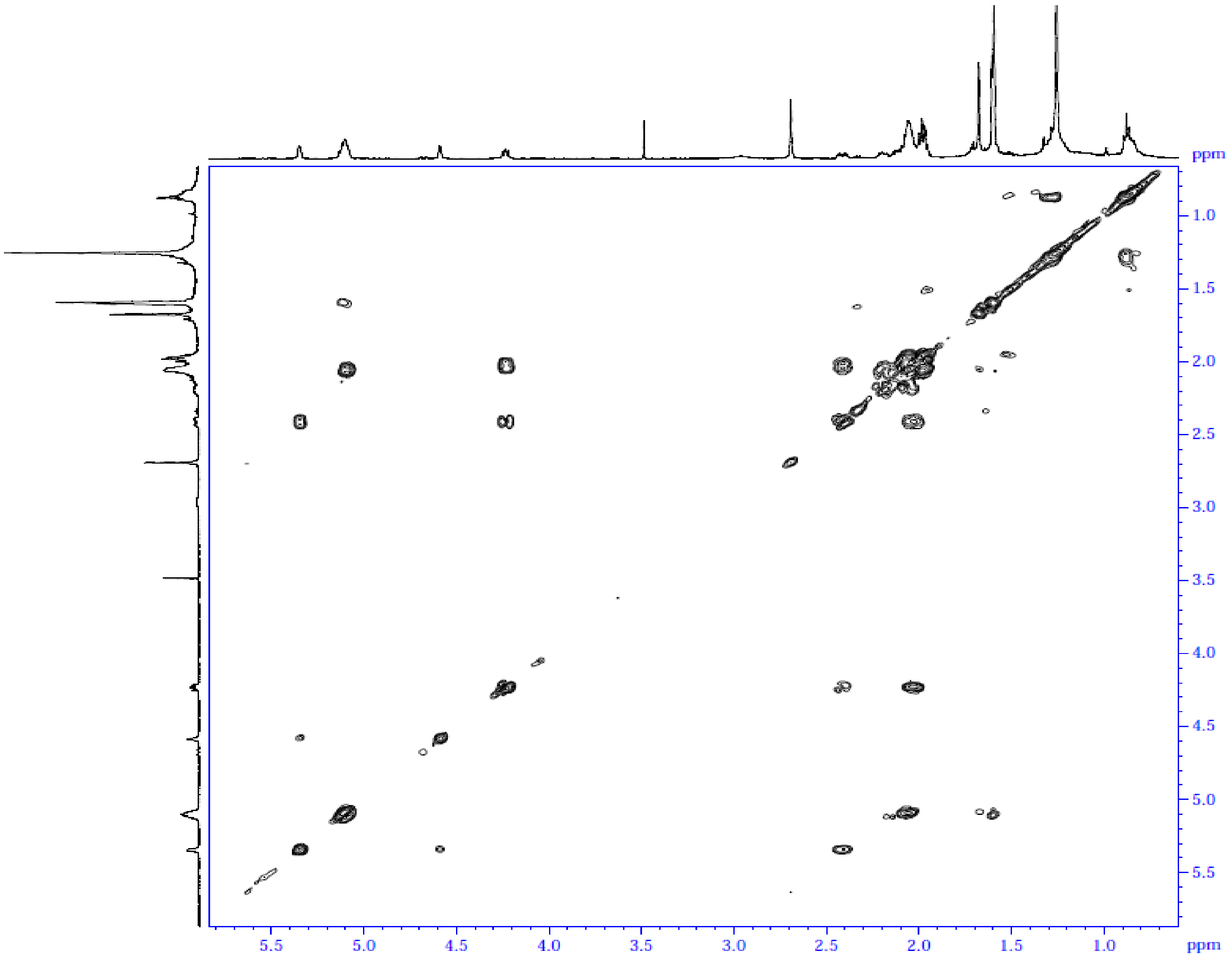

Figure S28. HMBC spectrum of compound **3** in $CDCl_3$.

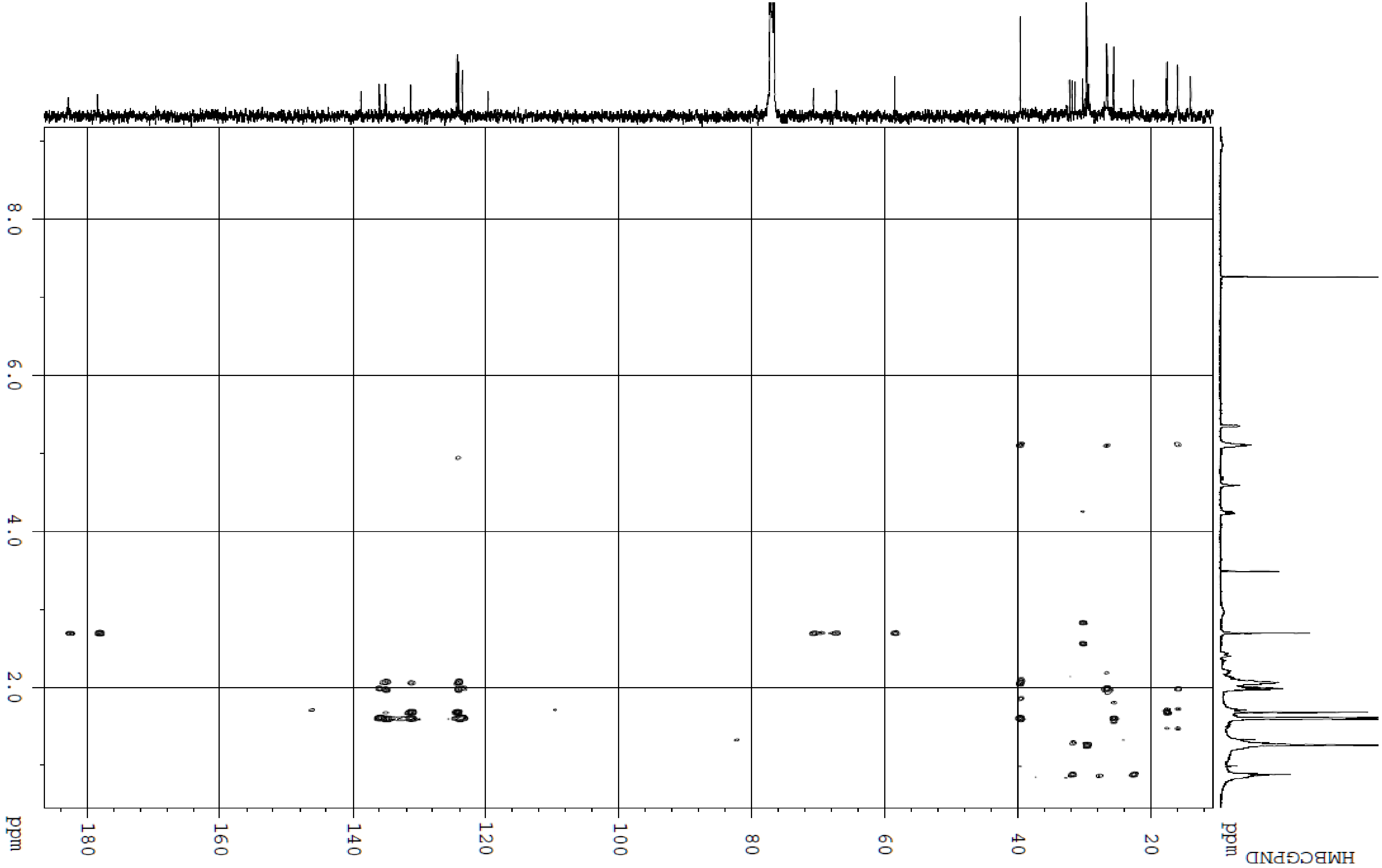

Figure S29. NOESY spectrum of compound **3** in $CDCl_3$.

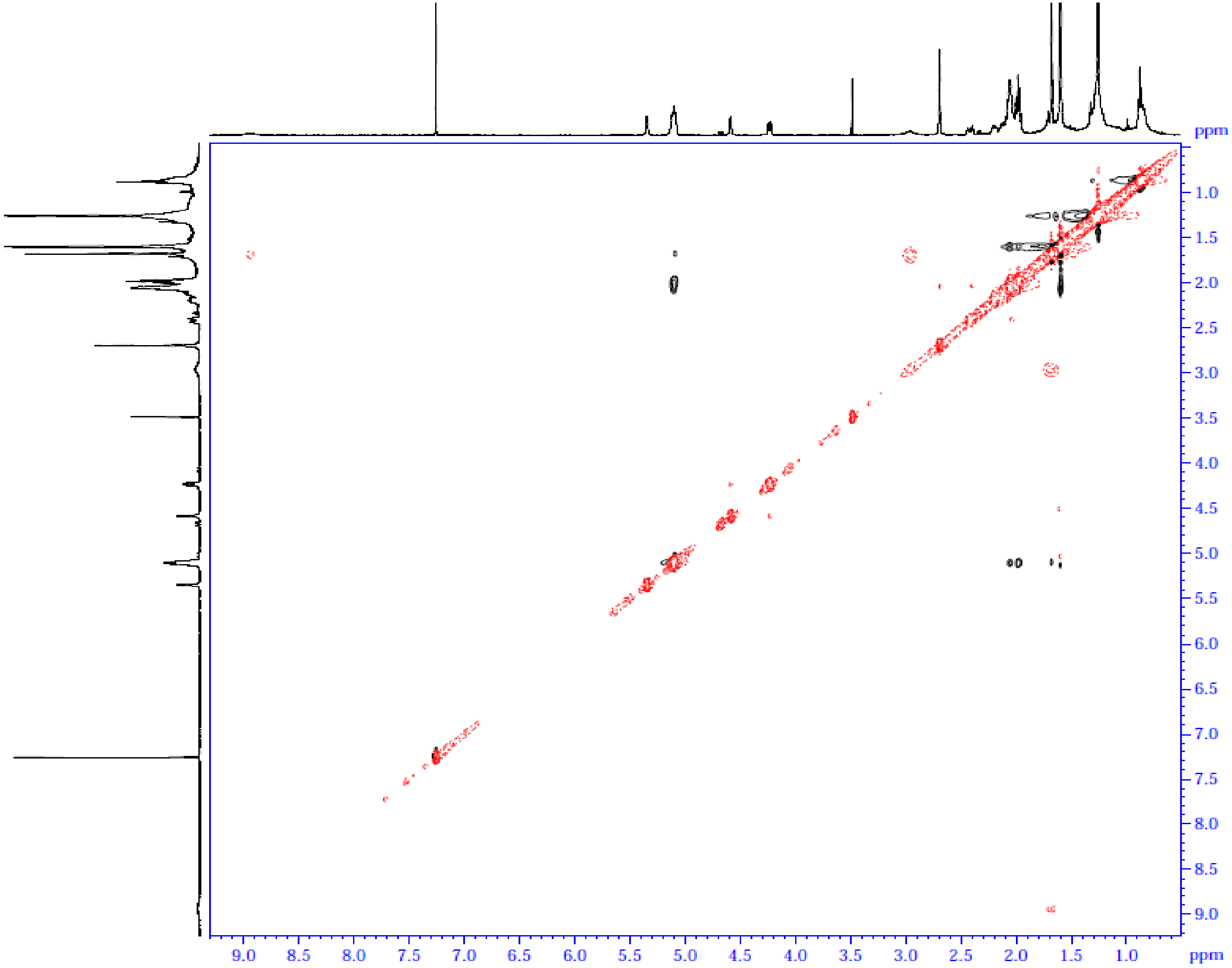

Figure S30. HRESIMS spectrum of compound **4**.

Elemental Composition Report

Page 1

Tolerance = 10.0 PPM / DBE: min = -1.5, max = 50.0
Selected filters: None

Monoisotopic Mass, Even Electron Ions
7 formula(e) evaluated with 1 results within limits (up to 50 closest results for each mass)
Elements Used:
C: 5-27 H: 10-50 O: 3-4 Na: 1-1

SIPI Q-Tof micro 04-Oct-2010,13:56:02
PSJ-B11 M.W=370 YA019 0.00000000
WQ10350H2 46 (1.589) AM (Cen,6, 80.00, Ar,5000.0,384.16,1.00); Sm (SG, 2x3.00); Cm (29:46) TOF MS ES+
9.00e4

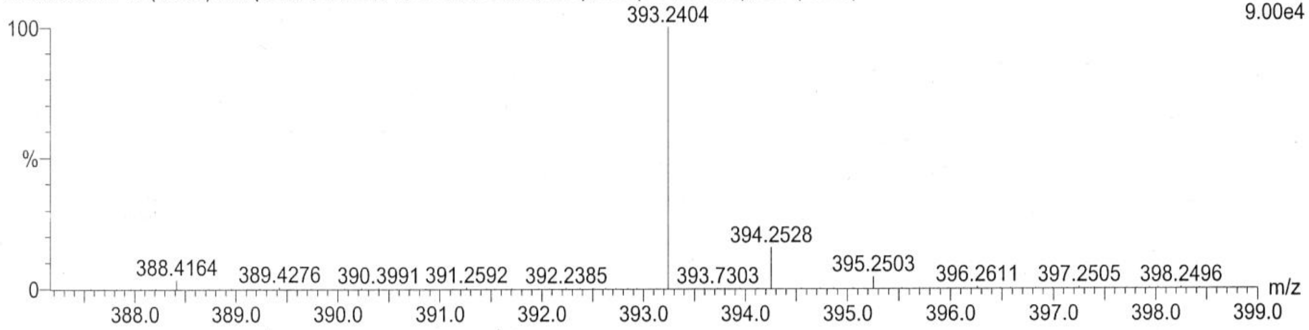

Minimum:	50.00				-1.5		
Maximum:	100.00		5.0	10.0	50.0		
Mass	RA	Calc. Mass	mDa	PPM	DBE	i-FIT	Formula
393.2404	100.00	393.2406	-0.2	-0.5	7.5	2754.3	C24 H34 O3 Na

Figure S31. IR spectrum of compound **4**.

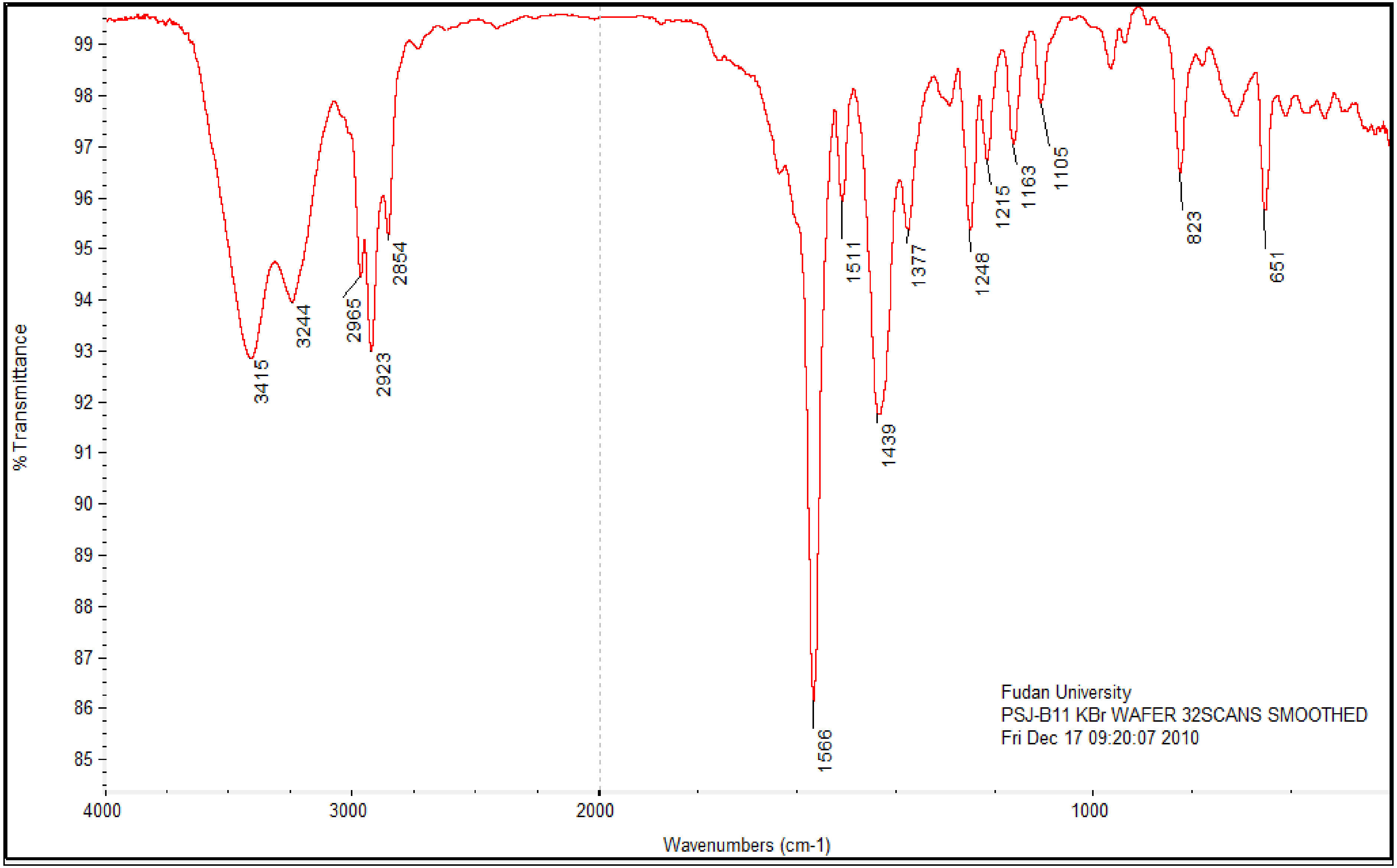

Figure S32. ^{1}H NMR spectrum of compound **4** in $CDCl_3$.

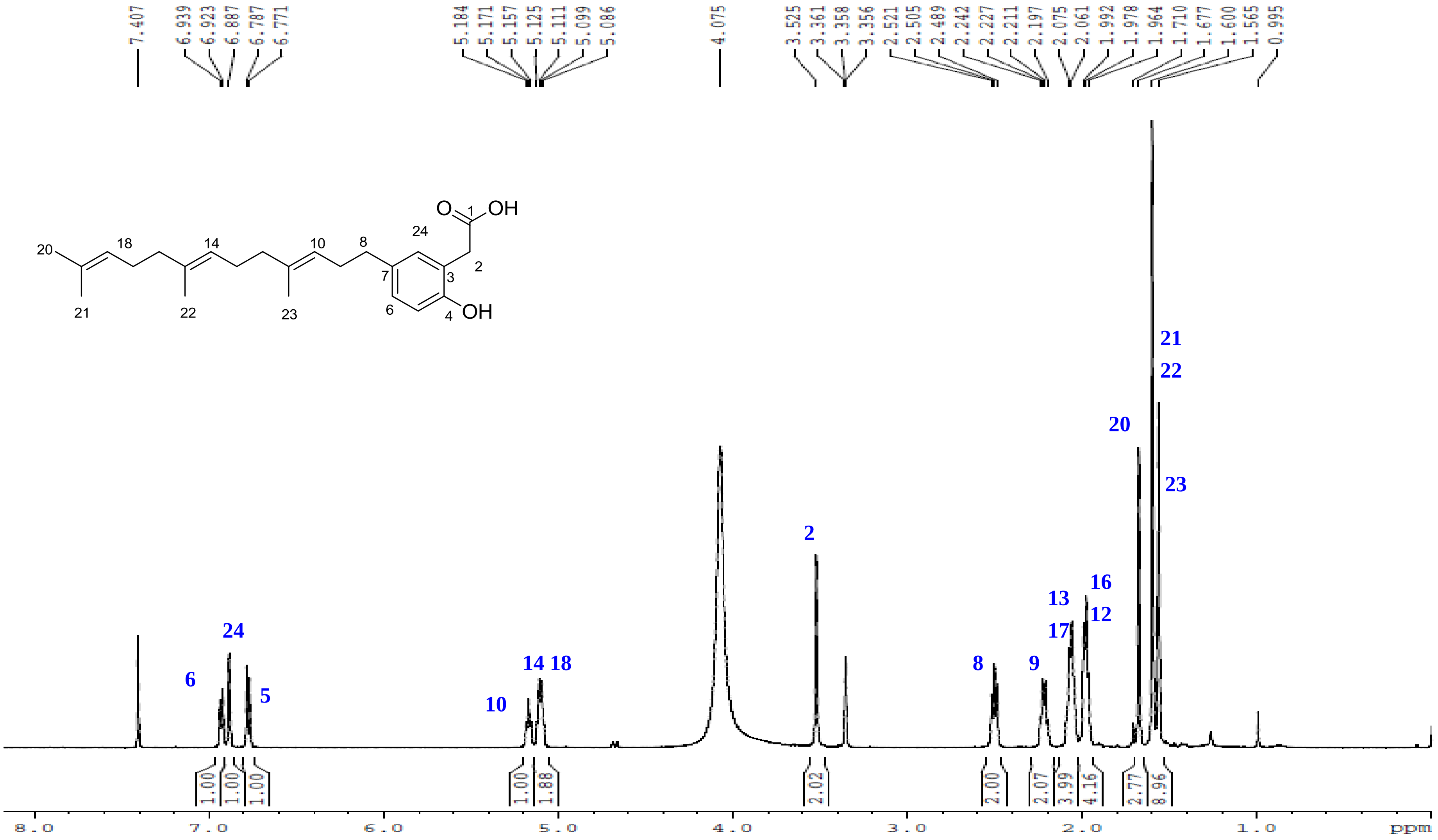

Figure S33. ^{13}C NMR spectrum of compound **4** in $CDCl_3$.

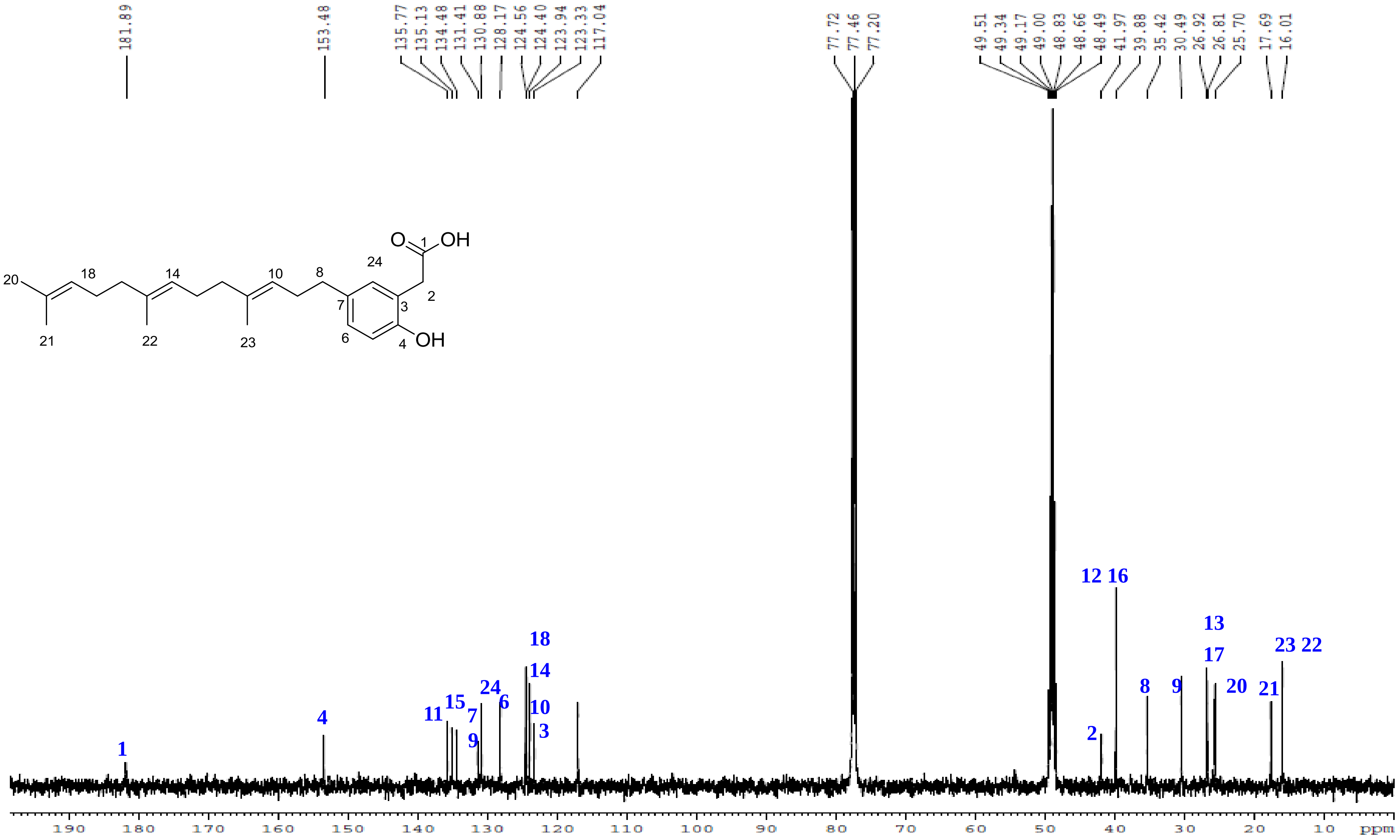

Figure S34. DEPT spectrum of compound **4** in $CDCl_3$.

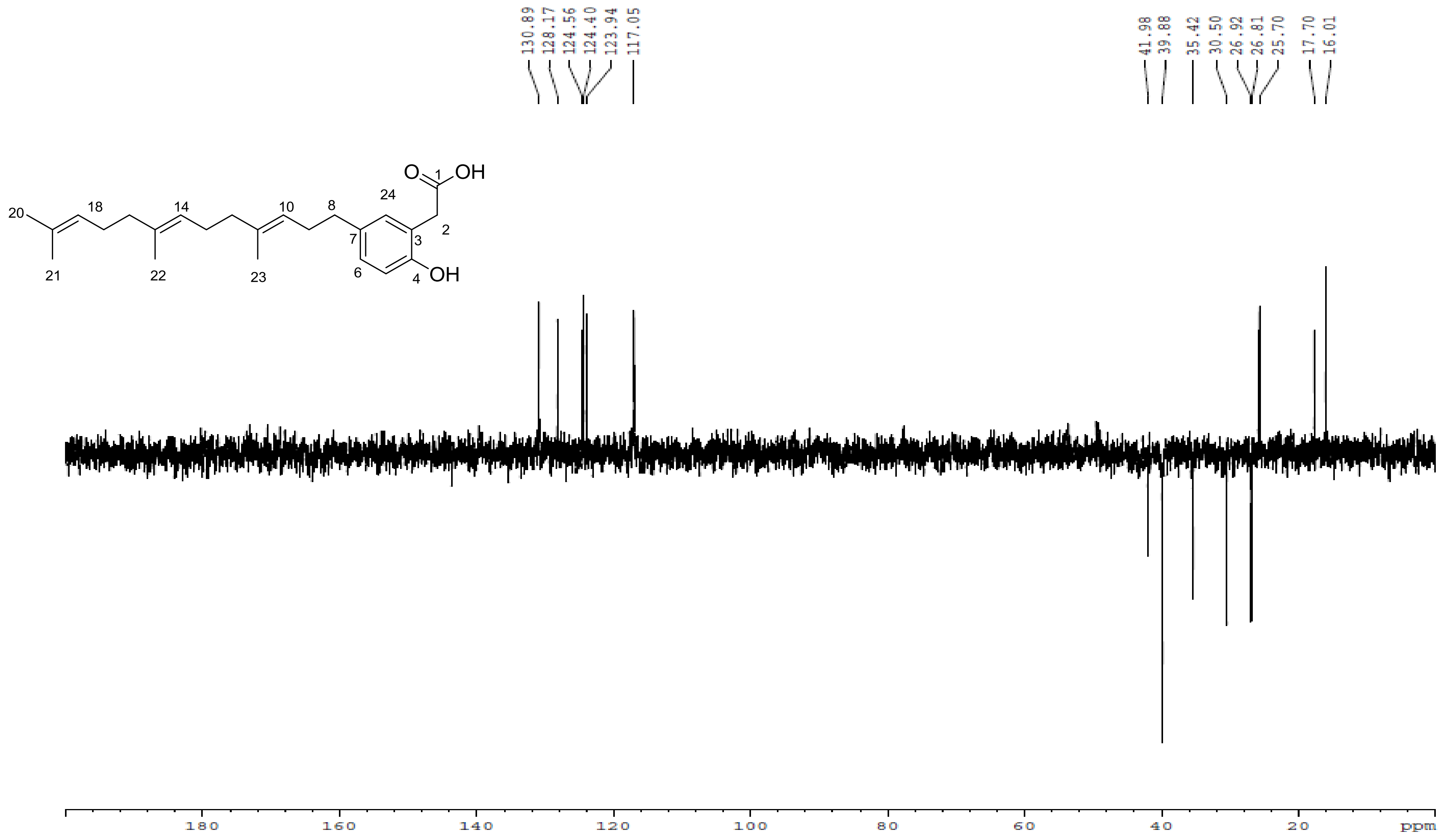

Figure S35. HSQC spectrum of compound **4** in $CDCl_3$.

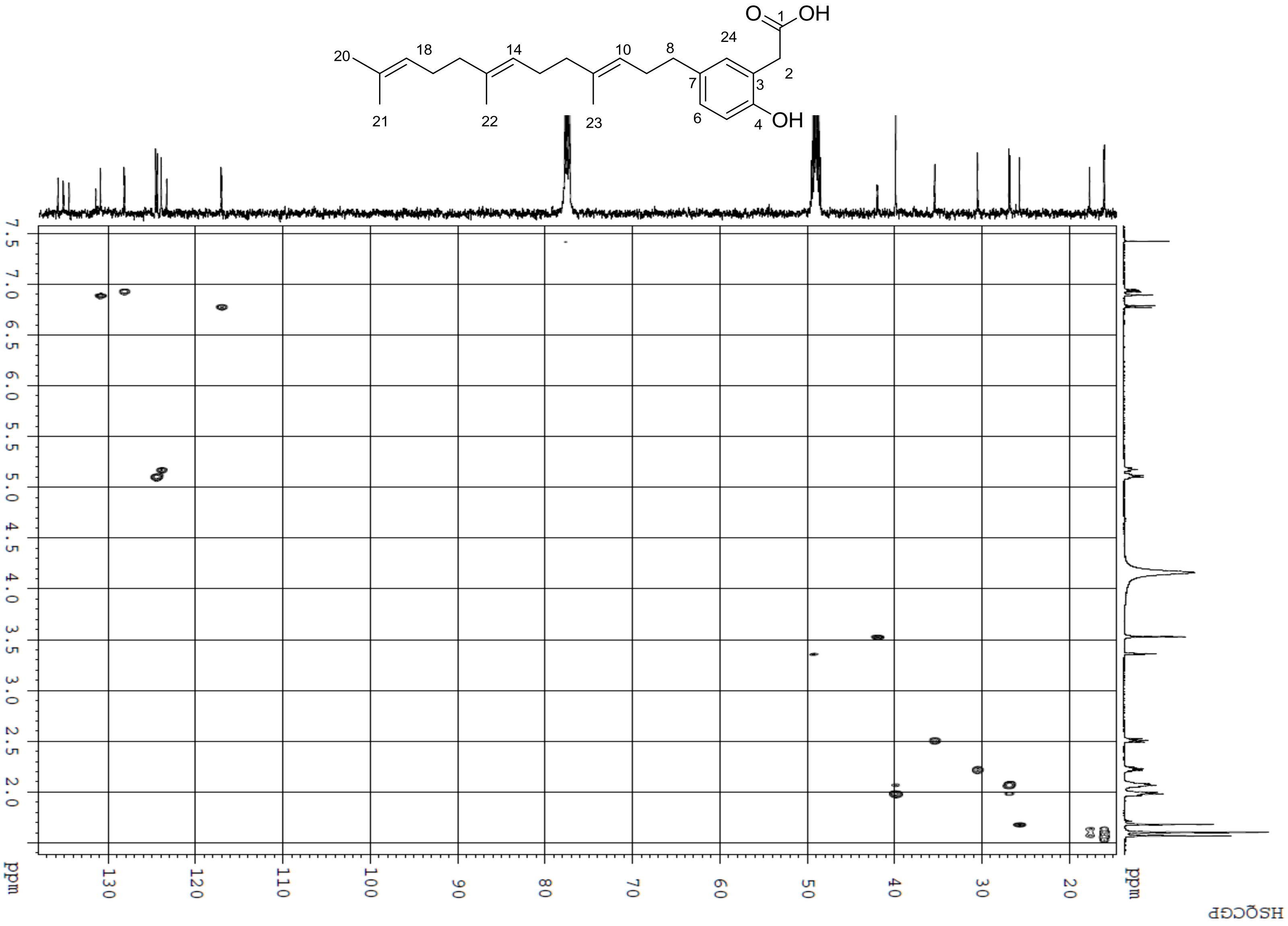

Figure S36. ^{1}H–^{1}H COSY spectrum of compound **4** in $CDCl_3$.

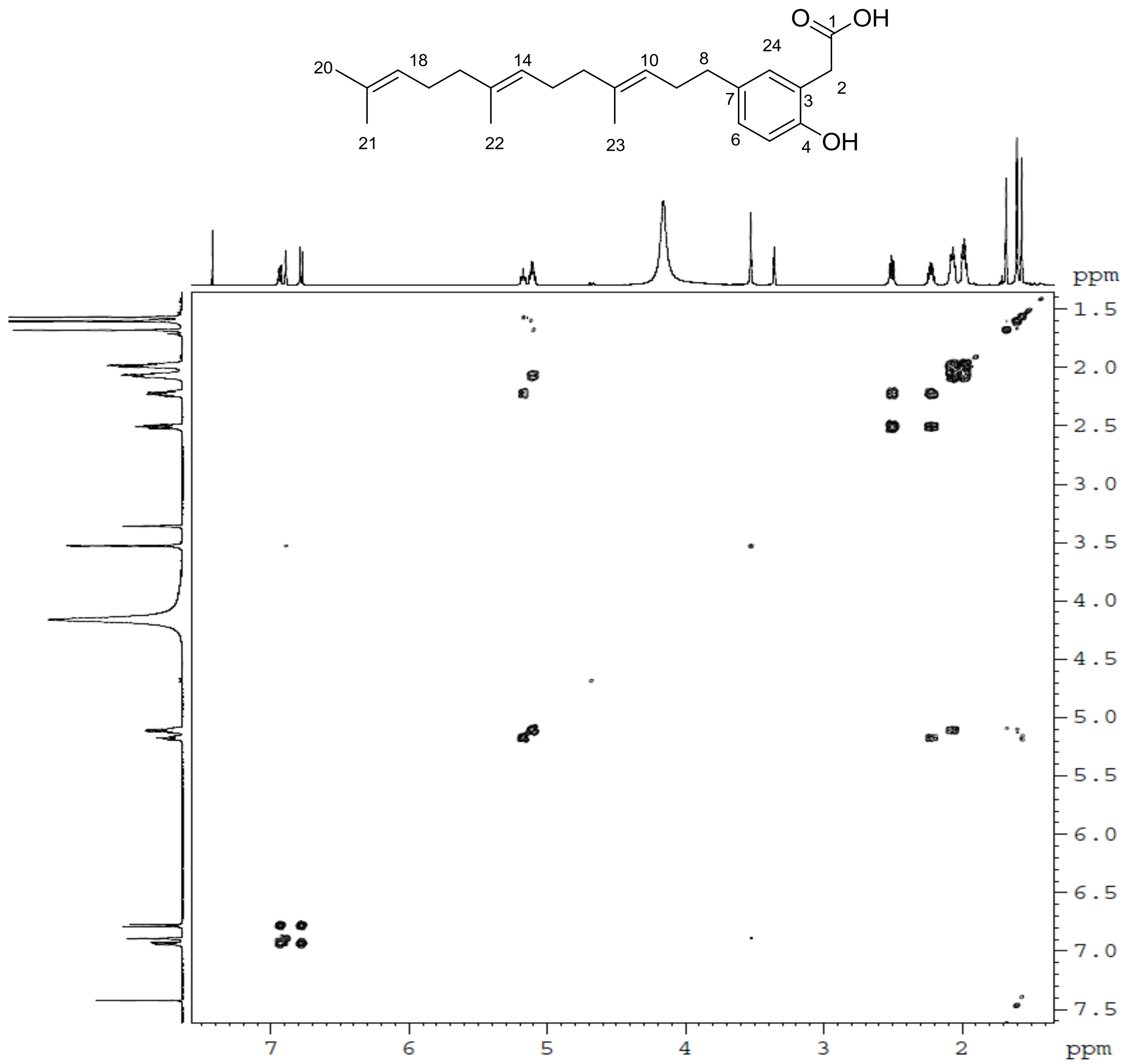

Figure S37. HMBC spectrum of compound **4** in $CDCl_3$.

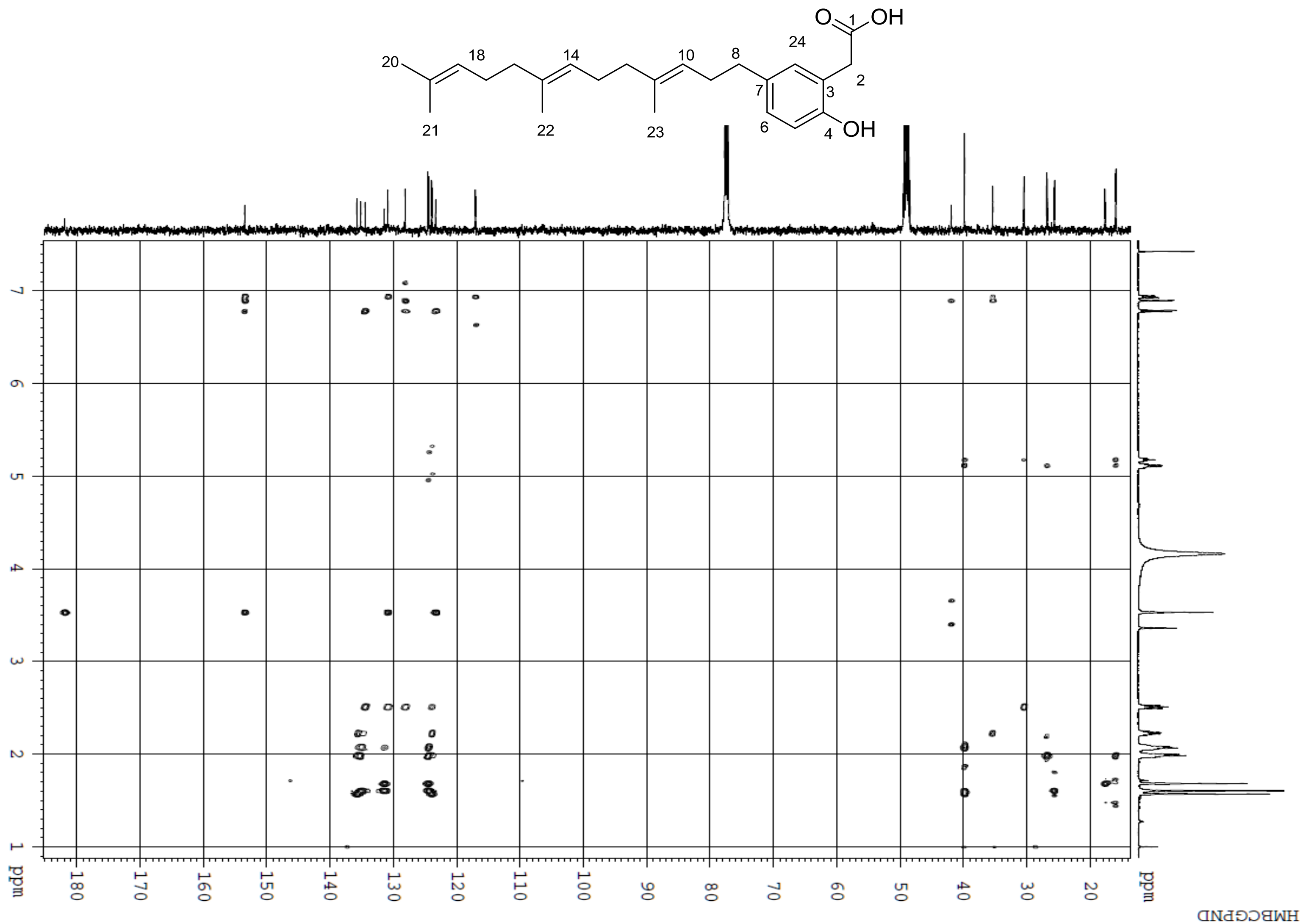

Figure S38. NOESY spectrum of compound **4** in $CDCl_3$.

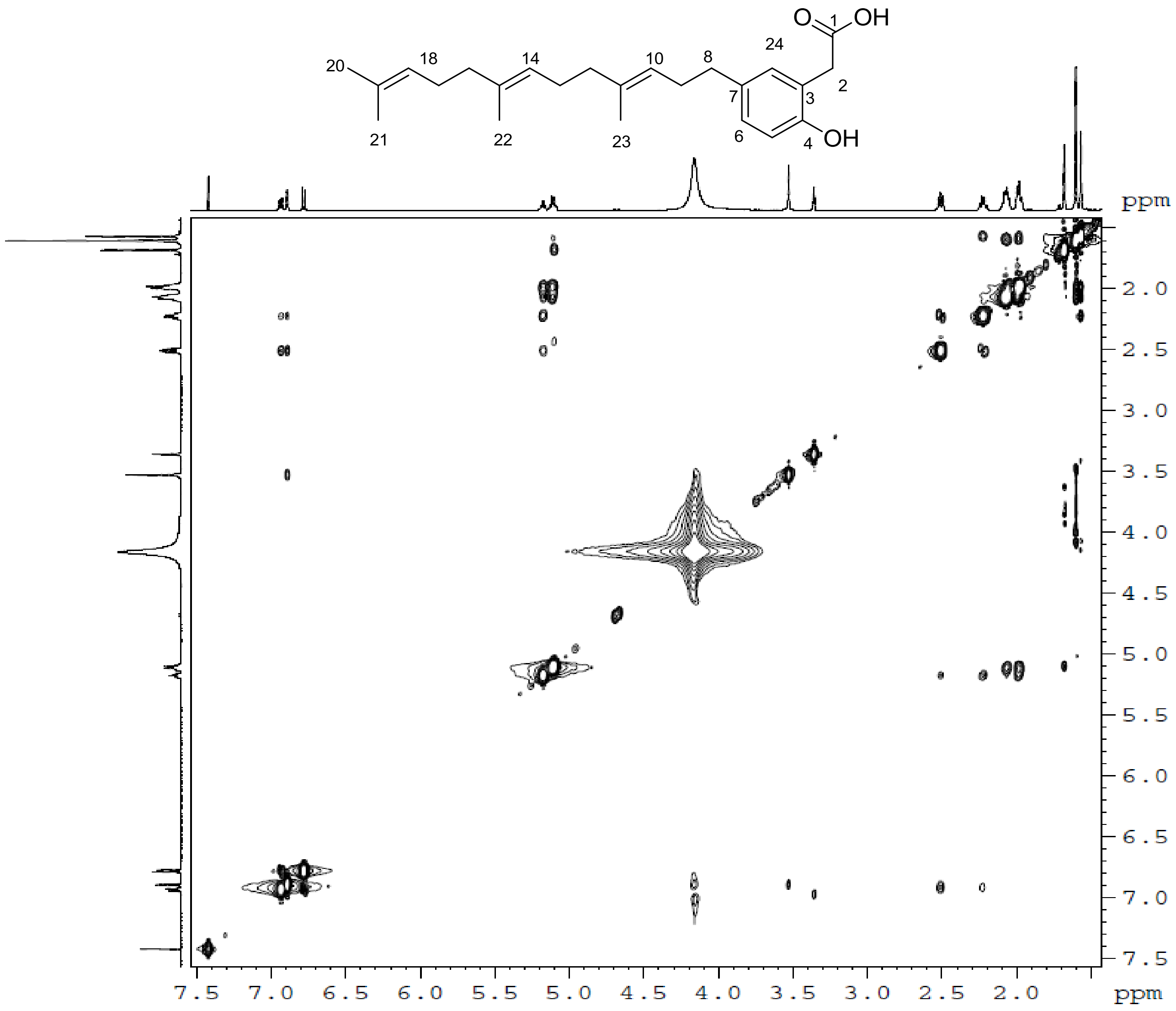

Figure S39. HRESIMS spectrum of compound **5**.

Elemental Composition Report

Page 1

Tolerance = 10.0 PPM / DBE: min = -1.5, max = 50.0
Selected filters: None

Monoisotopic Mass, Even Electron Ions
42 formula(e) evaluated with 1 results within limits (up to 50 closest results for each mass)
Elements Used:
C: 5-30 H: 5-45 N: 0-1 O: 1-6 Na: 1-1

SIPI Q-Tof micro 13-May-2010,13:10:28
PSJ-B8 M.W=369 YA019 0.00000000
WQ10182H1 63 (2.176) AM (Cen,6, 80.00, Ar,5000.0,385.24,0.70); Sm (SG, 2x3.00); Cm (49:66) TOF MS ES+
2.53e4

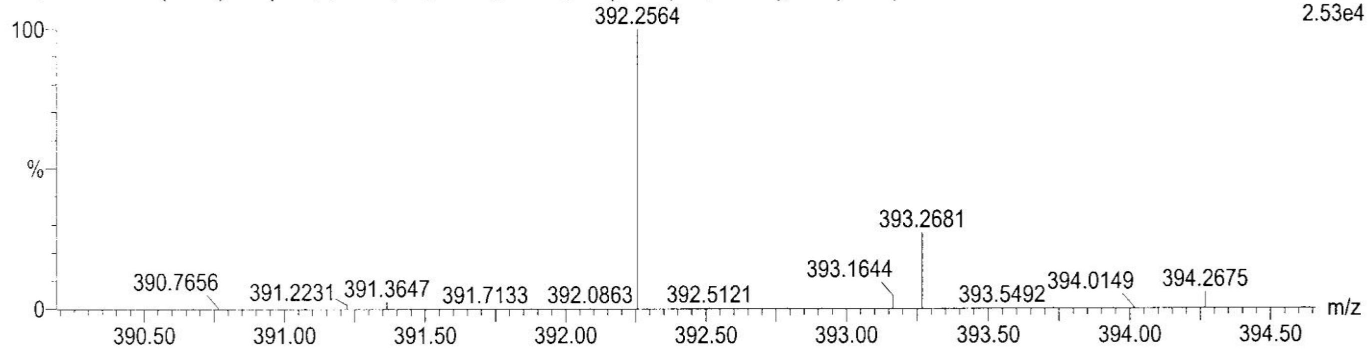

Minimum:	30.00				-1.5		
Maximum:	100.00		5.0	10.0	50.0		
Mass	RA	Calc. Mass	mDa	PPM	DBE	i-FIT	Formula
392.2564	100.00	392.2565	-0.1	-0.3	7.5	57.9	C24 H35 N O2 Na

Figure S40. IR spectrum of compound **5**.

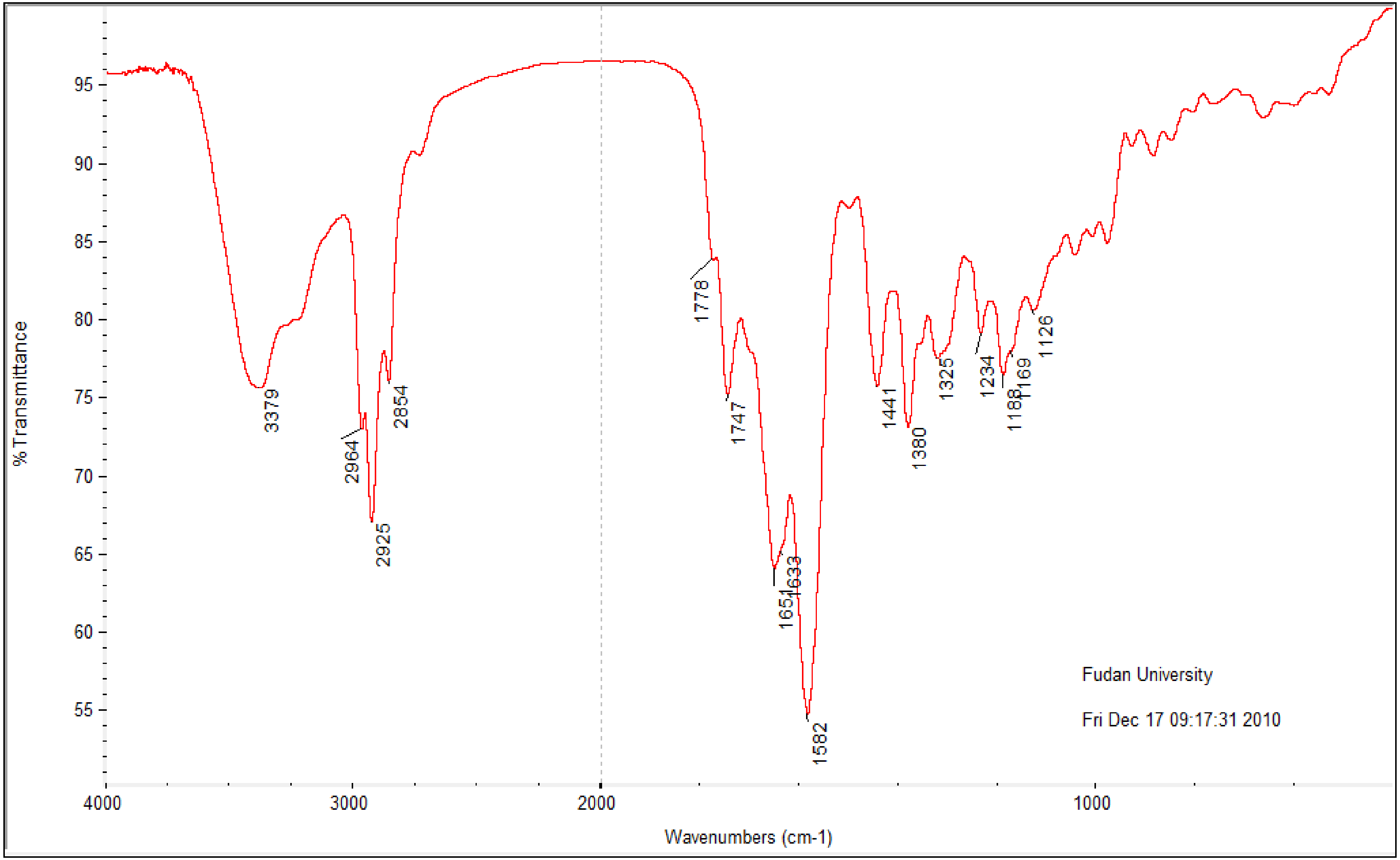

Figure S41. ^{1}H NMR spectrum of compound **5** in $CDCl_3$.

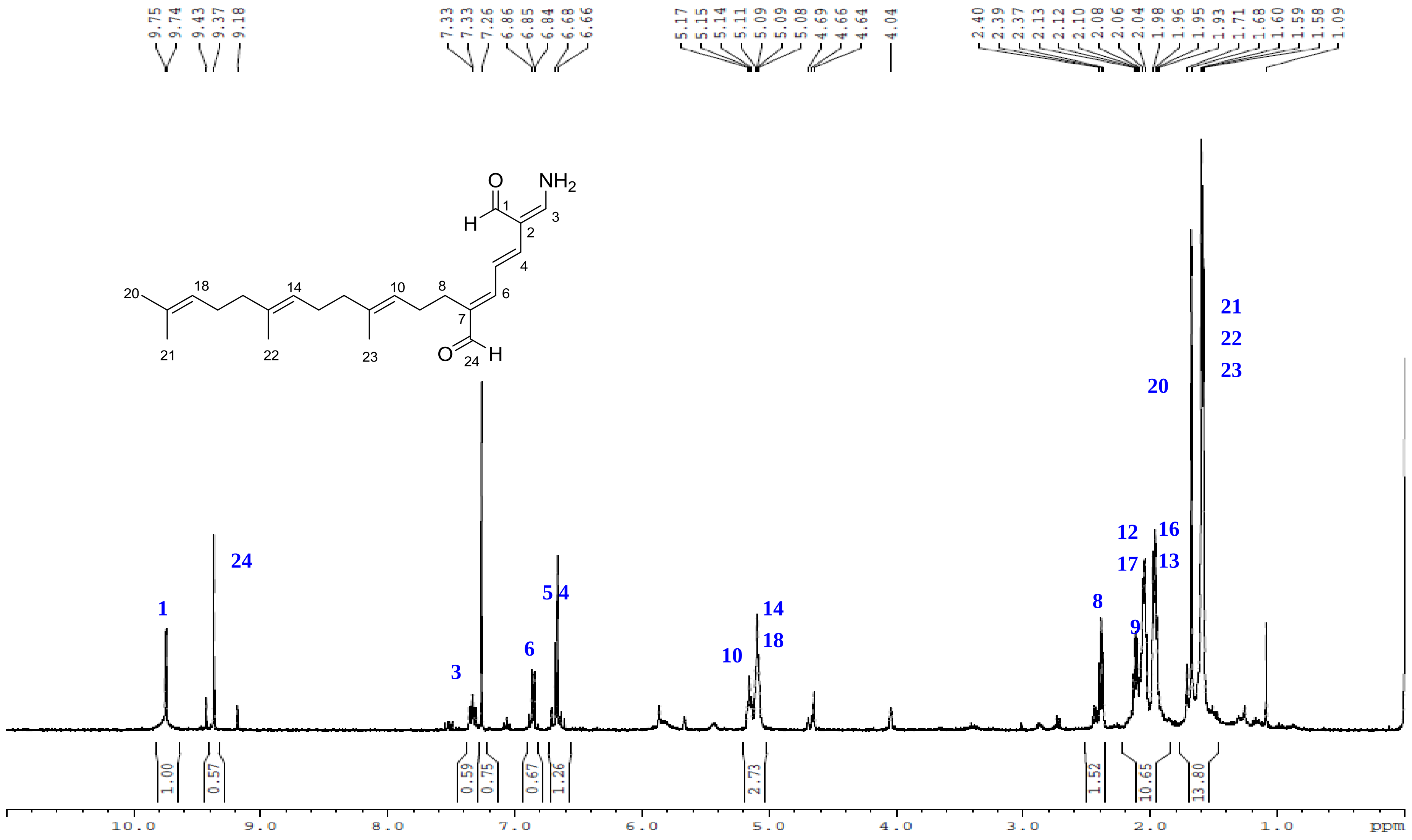

Figure S42. ^{1}H NMR spectrum of compound **5** in DMSO-d_6.

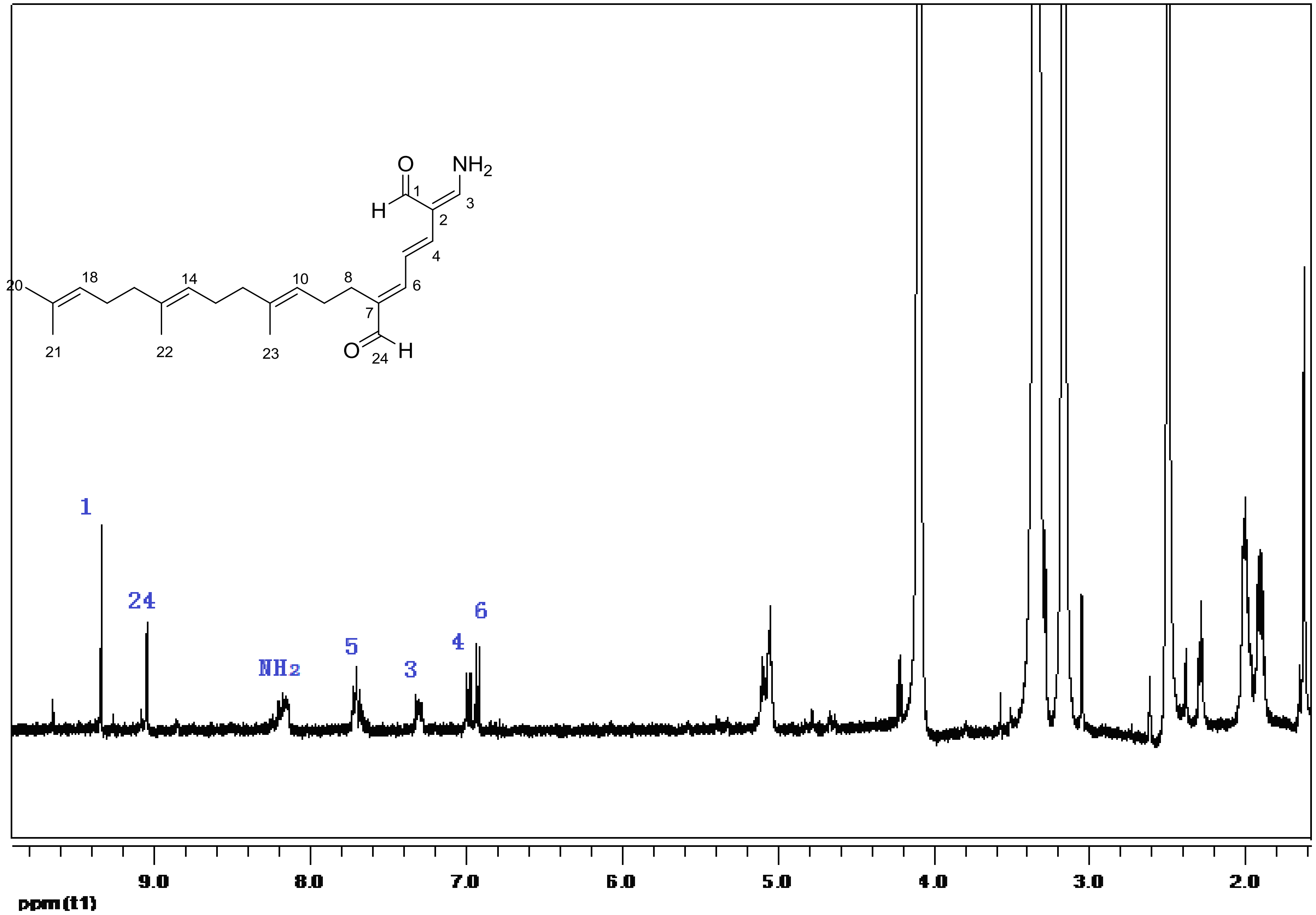

Figure S43. ^{13}C NMR spectrum of compound **5** in $CDCl_3$.

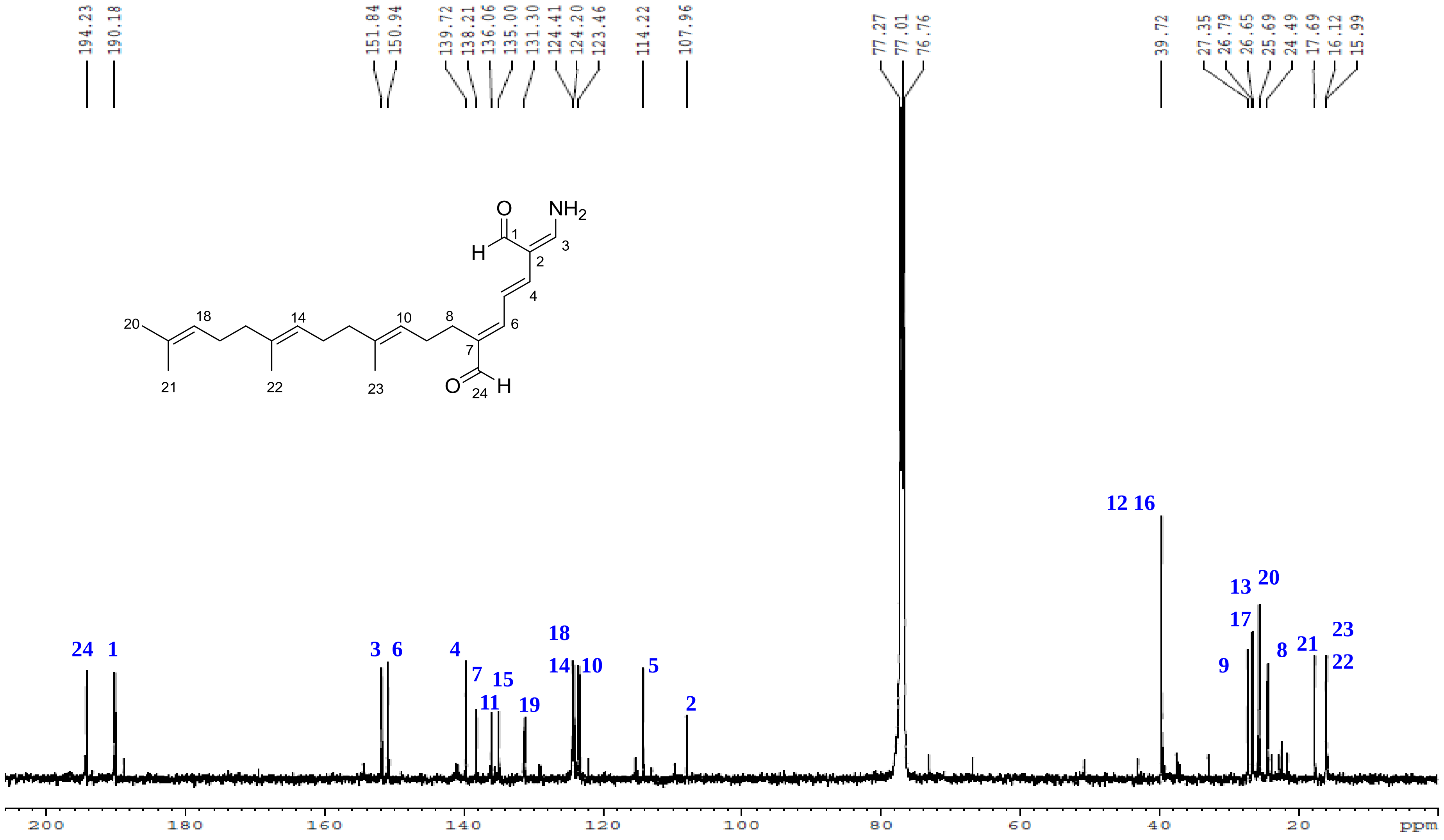

Figure S44. DEPT spectrum of compound **5** in $CDCl_3$.

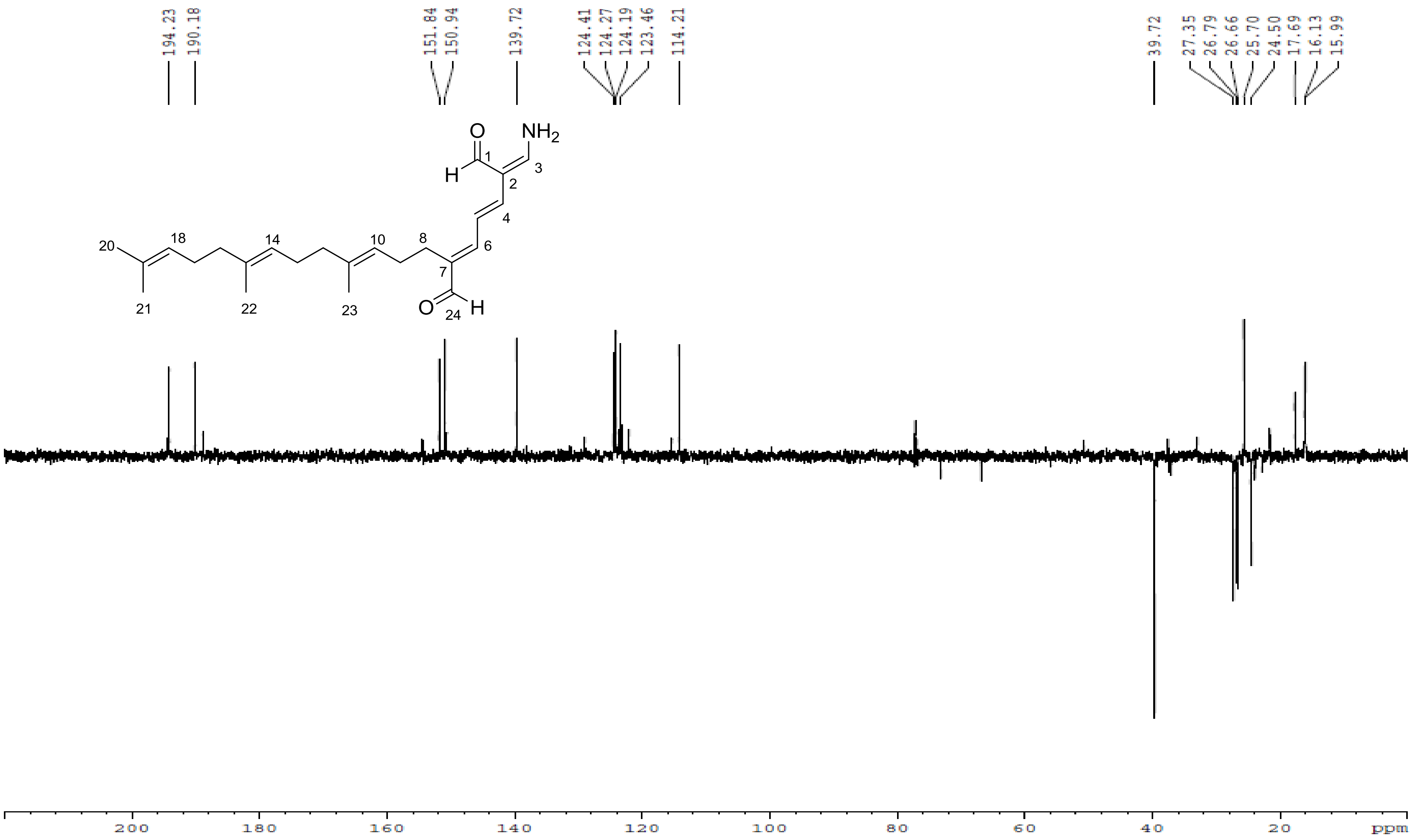

Figure S45. HSQC spectrum of compound **5** in $CDCl_3$.

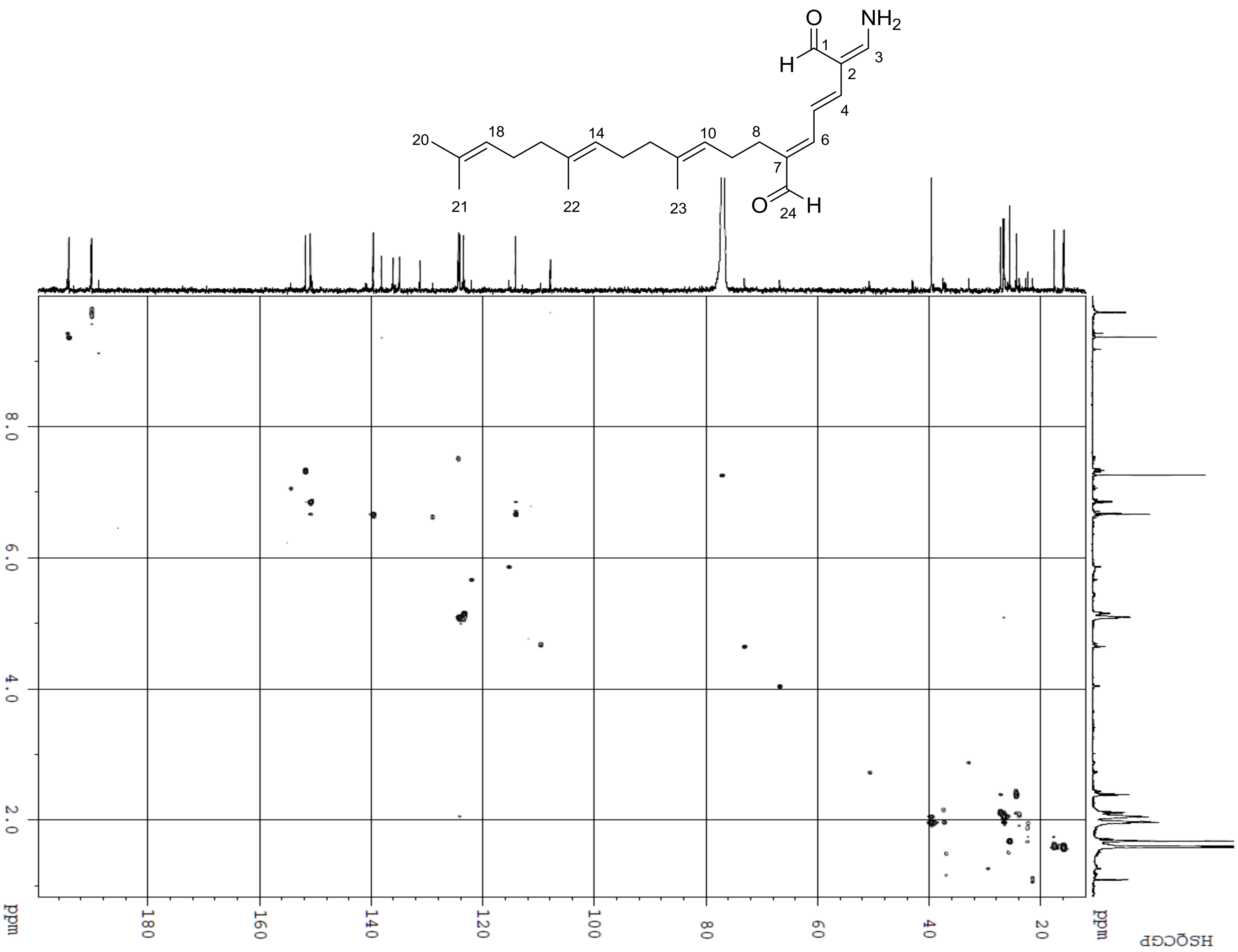

Figure S46. ^{1}H–^{1}H COSY spectrum of compound **5** in $CDCl_3$.

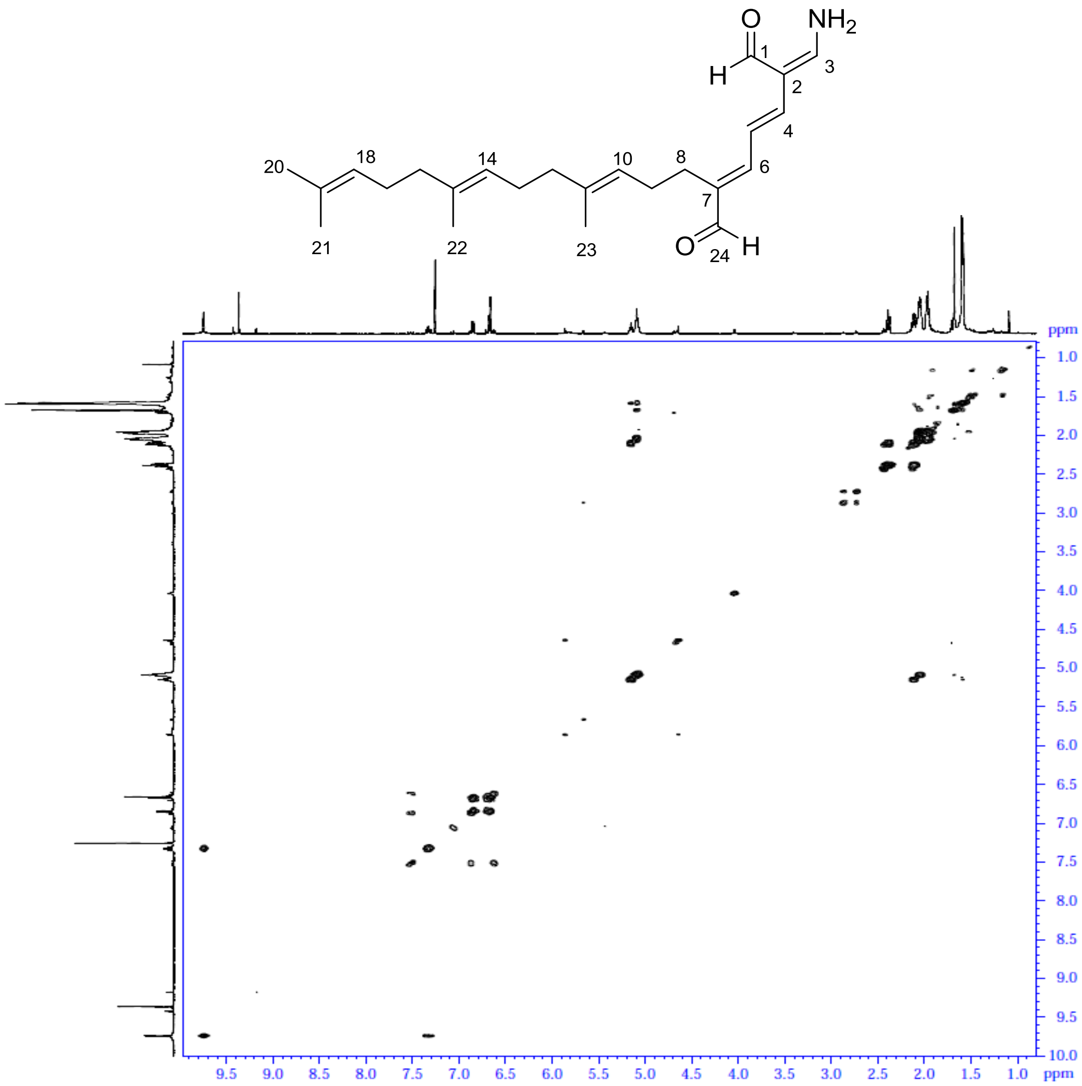

Figure S47. HMBC spectrum of compound **5** in $CDCl_3$.

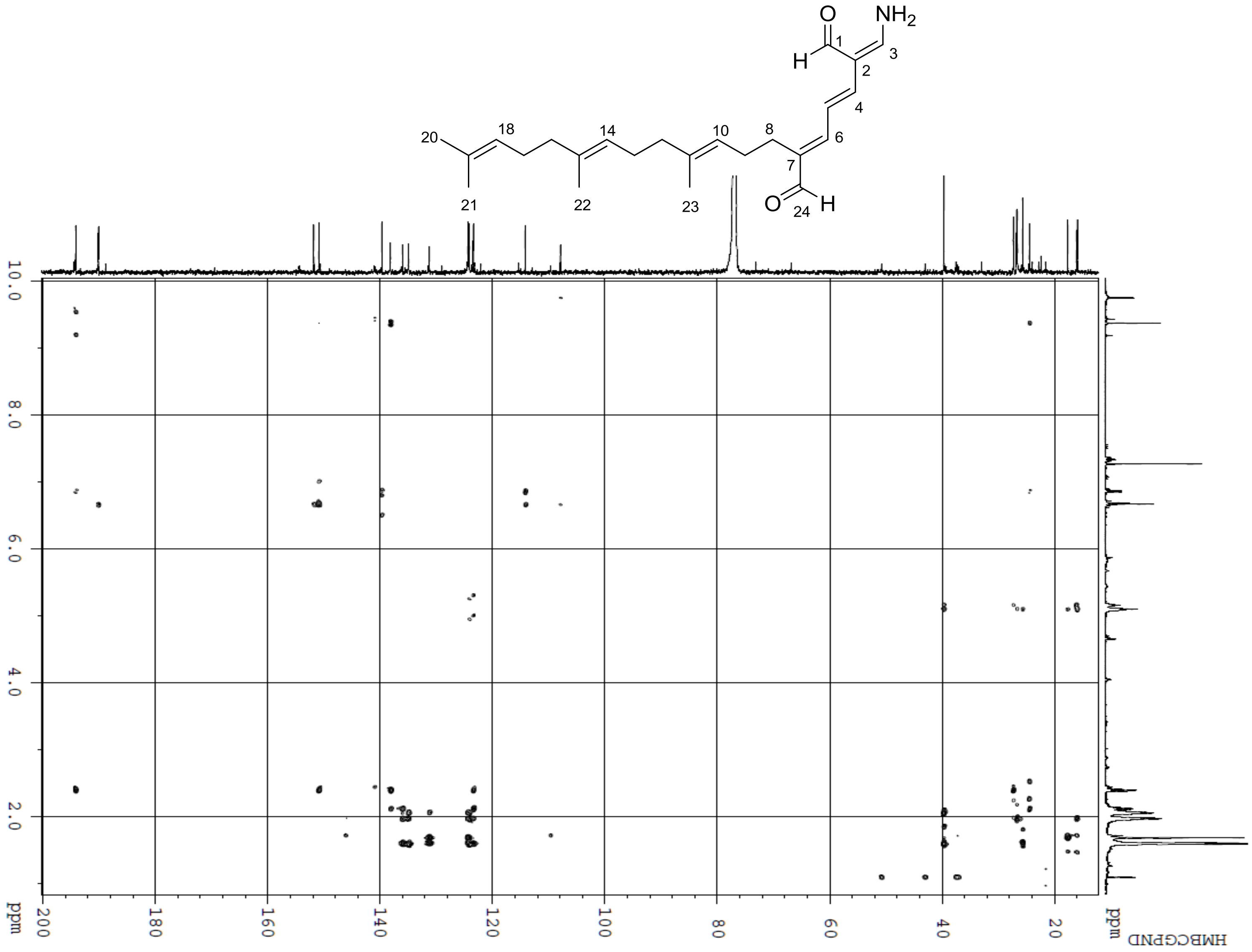

Figure S48. NOESY spectrum of compound **5** in $CDCl_3$.

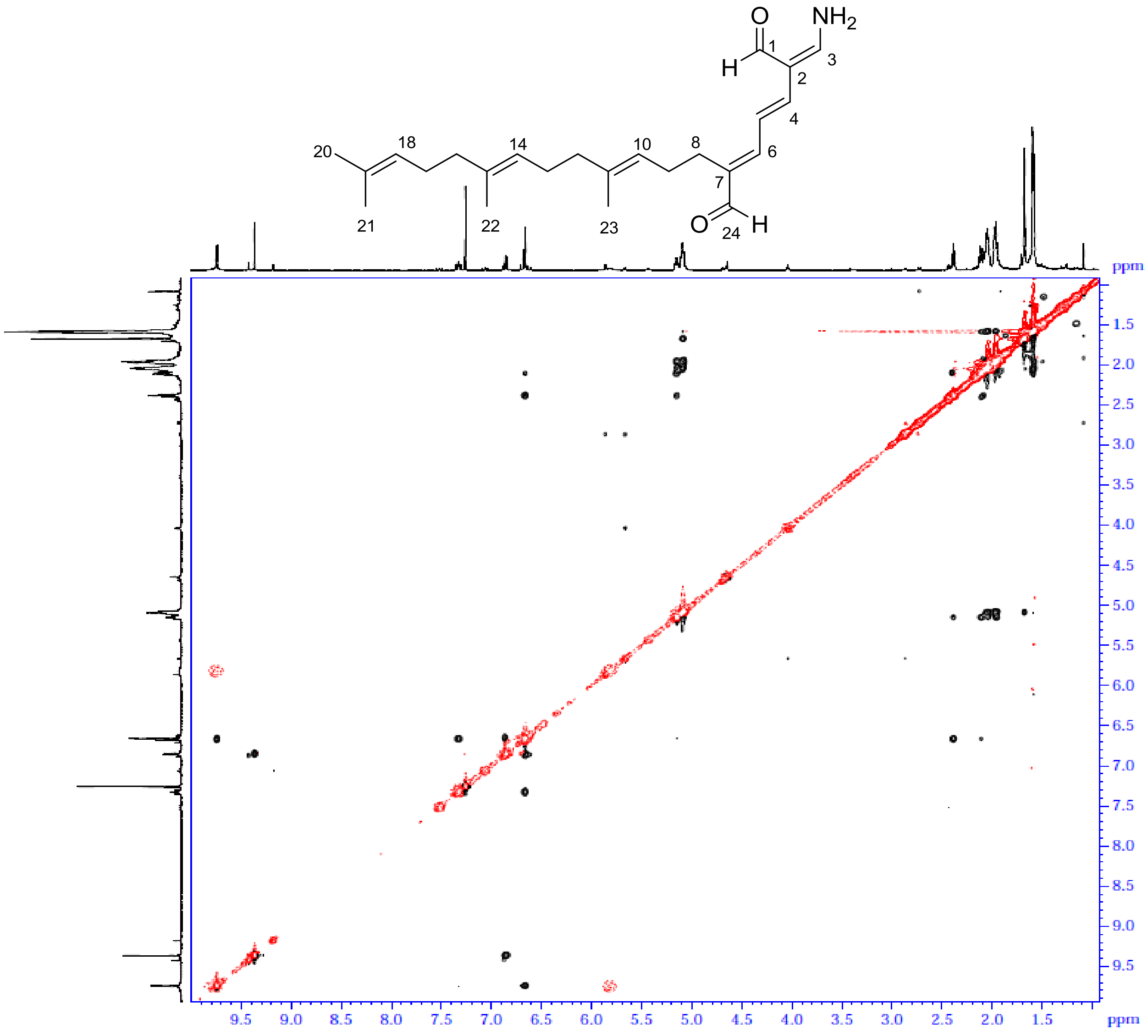

Figure S49. ^{1}H NMR data of MTPA esters (**2*S***, **2*R***, **3*S***, **3*R***).

^{1}H NMR data of **2*S*** (500 MHz, $CDCl_3$): δ 2.893 (1H, m, H-2a), 2.769 (1H, m, H-2b), 3.858 (1H, m, H-4), 2.313 (1H, m, H-5a), 2.163 (1H, m, H-5b), 5.462 (1H, br s, H-6).
^{1}H NMR data of **2*R*** (500 MHz, $CDCl_3$): δ 2.901 (1H, m, H-2a), 2.782 (1H, m, H-2b), 3.860 (1H, m, H-4), 2.231 (1H, m, H-5a), 2.143 (1H, m, H-5b), 5.457 (1H, br s, H-6).
^{1}H NMR data of **3*S*** (500 MHz, $CDCl_3$): δ 2.733 (1H, m, H-2), 4.141 (1H, m, H-4), 2.419 (1H, m, H-5a), 2.208 (1H, m, H-5b), 5.381 (1H, br s, H-6).
^{1}H NMR data of **3*R*** (500 MHz, $CDCl_3$): δ 2.735 (1H, m, H-2), 4.145 (1H, m, H-4), 2.385 (1H, m, H-5a), 2.194 (1H, m, H-5b), 5.363 (1H, br s, H-6).

References

1. Frisch, M.J.; Trucks, G.W.; Schlegel, H.B.; Scuseria, G.E.; Robb, M.A.; Cheeseman, J.R.; Montgomery, J.A., Jr.; Vreven, T.; Kudin, K.N.; Burant, J.C.; *et al. Gaussian 03*, revision D.01; Gaussian, Inc.: Wallingford, CT, USA, 2013.
2. Available online: http://www.gaussian.com (accessed on 11 December 2013).

© 2014 by the authors; licensee MDPI, Basel, Switzerland. This article is an open access article distributed under the terms and conditions of the Creative Commons Attribution license (http://creativecommons.org/licenses/by/3.0/).
